# Genome and Phenotype Microarray Analyses of *Rhodococcus* sp. BCP1 and *Rhodococcus opacus* R7: Genetic Determinants and Metabolic Abilities with Environmental Relevance

**DOI:** 10.1371/journal.pone.0139467

**Published:** 2015-10-01

**Authors:** Alessandro Orro, Martina Cappelletti, Pasqualina D’Ursi, Luciano Milanesi, Alessandra Di Canito, Jessica Zampolli, Elena Collina, Francesca Decorosi, Carlo Viti, Stefano Fedi, Alessandro Presentato, Davide Zannoni, Patrizia Di Gennaro

**Affiliations:** 1 Institute of Biomedical Technology, CNR, Segrate, Milano, Italy; 2 Department of Pharmacy and Biotechnology, University of Bologna, Bologna, Italy; 3 Department of Biotechnology and Biosciences, University of Milano-Bicocca, Milano, Italy; 4 Department of Earth and Environmental Sciences, University of Milano-Bicocca, Milano, Italy; 5 Department of Agrifood Production and Environmental Sciences, University of Firenze, Firenze, Italy; University Paris South, FRANCE

## Abstract

In this paper comparative genome and phenotype microarray analyses of *Rhodococcus* sp. BCP1 and *Rhodococcus opacus* R7 were performed. *Rhodococcus* sp. BCP1 was selected for its ability to grow on short-chain *n*-alkanes and *R*. *opacus* R7 was isolated for its ability to grow on naphthalene and on *o*-xylene. Results of genome comparison, including BCP1, R7, along with other *Rhodococcus* reference strains, showed that at least 30% of the genome of each strain presented unique sequences and only 50% of the predicted proteome was shared. To associate genomic features with metabolic capabilities of BCP1 and R7 strains, hundreds of different growth conditions were tested through Phenotype Microarray, by using Biolog plates and plates manually prepared with additional xenobiotic compounds. Around one-third of the surveyed carbon sources was utilized by both strains although R7 generally showed higher metabolic activity values compared to BCP1. Moreover, R7 showed broader range of nitrogen and sulphur sources. Phenotype Microarray data were combined with genomic analysis to genetically support the metabolic features of the two strains. The genome analysis allowed to identify some gene clusters involved in the metabolism of the main tested xenobiotic compounds. Results show that R7 contains multiple genes for the degradation of a large set of aromatic and PAHs compounds, while a lower variability in terms of genes predicted to be involved in aromatic degradation was found in BCP1. This genetic feature can be related to the strong genetic pressure exerted by the two different environment from which the two strains were isolated. According to this, in the BCP1 genome the *smo* gene cluster involved in the short-chain *n*-alkanes degradation, is included in one of the unique regions and it is not conserved in the *Rhodococcus* strains compared in this work. Data obtained underline the great potential of these two *Rhodococcus* spp. strains for biodegradation and environmental decontamination processes.

## Introduction

The *Rhodococcus* genus comprises of Gram-positive, non-motile, non-sporulating, aerobic bacteria, with a high G+C content and mycolic acid-containing cell wall. This genus was firstly proposed by Zopf and revised by Tsukamura [1] and Goodfellow and Alderson [2]; it nowadays contains nearly 50 recognized species (http://www.bacterio.net/rhodococcus.html) [3, 4]. Members of *Rhodoccocus* genus are widely distributed in soil, water and marine sediments [5]. Some of them have also evolved for pathogenicity in humans, animals (i.e. *R*. *equi*) [6] and plants (i.e. *R*. *fascians*) [7]. Thanks to their broad catabolic diversity and their tolerance to various environmental stress, *Rhodococcus* spp. play an important role in nutrient cycling and have potential applications in bioremediation, biotransformations, and biocatalysis [3]. Compounds that are metabolically transformed or degraded by these bacteria are aliphatic and aromatic hydrocarbons, oxygenates, halogenated compounds, including polychlorinated biphenyls, nitro-aromatics, heterocyclic compounds, nitriles and various herbicides [5]. *Rhodococcus* spp. are able to perform steroid modifications, enantio-selective synthesis, production of amides from nitriles, and to convert plant secondary metabolites found in soil and rhizosphere, such as alkaloids, terpenes, and sterols [8]. Various *Rhodococcus* strains are also efficient at removing sulphur from coal and petroleum products [9, 10].

In line with the immense catabolic diversity shown by the members of this genus, *Rhodococcus* spp. are characterized to possess large and complex genomes, which contain a multiplicity of catabolic genes, a high genetic redundancy of biosynthetic pathways and a sophisticated regulatory network [11]. Many of them also possess a variety of large linear plasmids and smaller circular plasmids that contribute to and also explain the immense repertoire of catabolic abilities [3]. Up to date, some *Rhodococcus* genomes have been sequenced with different level of completeness (http://www.ncbi.nlm.nih.gov/genome/Rhodococcus) [12]. The first genome completely sequenced belongs to *Rhodococcus jostii* RHA1 strain. This strain was isolated from lindane- contaminated soil for its exceptional ability to aerobically degrade polychlorinated biphenyls (PCBs) [13]. It was further described to utilize a wide range of aromatic compounds, carbohydrates, nitriles and steroids as sole carbon and energy sources. Analyses of the 9.7 Mb large genome of RHA1 provided the evidence of catabolic pathway redundancy and horizontal gene transfer events [14]. Recently, *Rhodococcus opacus* PD630 and B4 strains have been completely sequenced. Regarding these two strains, studies focused on the production and accumulation of energy-rich triacylglycerols in PD630 and on the tolerance to organic solvents in B4 [15, 16]. Compared to the increasing number of *Rhodococcus* spp. genomes that have been sequenced, very limited studies are available on their wide metabolic abilities and on their potentials for biodegradation activities related to genomic features. A high-throughput assessment of metabolic abilities is given by Phenotype Microarray system, which consists of 96-well plates provided with different conditions of growth (substrates, metabolites, pH conditions, toxic chemicals) for bacteria in order to generate phenotypic data [17]. This technology has the potential to accelerate the functional characterization of genes also deriving from whole-genome sequencing. This approach has been recently used to describe the respiration, viability and growth of several environmentally and clinically relevant bacteria species like *Mycobacterium*, *Pseudomonas*, *Cronobacter* and *Sinorhizobium* [18–21]. *R*. *jostii* RHA1 and *R*. *opacus* PD630 were also tested for the growth on a limited number of carbon sources using Phenotype Microarray system [16]. This work focuses on the analysis of metabolic capabilities and genetic features of *Rhodococcus* sp. BCP1 and *Rhodococcus opacus* R7 whose genomes have been recently sequenced [22, 23]. *R*. *opacus* R7 is a Gram-positive bacterium isolated from a polycyclic aromatic hydrocarbon contaminated soil for its ability to grow on naphthalene and *o*-xylene [24]. It was further described for its ability to utilize several long- and medium-chain *n*-alkanes [25]. R7 has one of the largest bacterial genomes (around 10.1 Mb) with a G+C content of 66.0% [23]. *Rhodococcus* sp. BCP1 was selected from an aerobic butane-utilizing consortium as the prevailing isolate able to co-metabolize chloroform, vinyl chloride, and trichloroethylene [26–28]. As BCP1 also catabolizes a wide range of aliphatic, alicyclic, and carboxylated alkanes, it represents a strain of considerable environmental and industrial interest [29, 22].

In the present paper, a comparative genome analysis was performed to investigate the genomic differences between *R*. *opacus* R7 and *Rhodococcus* sp. BCP1 and between these two strains and those completely sequenced (*R*. *jostii* RHA1, *R*. *opacus* PD630, *R*. *opacus* B4) and/or taxonomically correlated (*R*. *pyridinivorans* SB3094). Additionally, a phenotypic screening of R7 and BCP1 strains was conducted using Phenotype Microarray with commercially available microtiter plates (pre-loaded substrates) and with microtiter plates manually prepared with additional organic/xenobiotic compounds. Phenotype microarray data were combined with genomic analysis to genetically support the metabolic features of these two bacterial strains with environmental and industrial relevance.

## Materials and Methods

### Genome sequencing and annotation

The genome sequencing of the two *Rhodococcus opacus* R7 (CIP107348) and *Rhodococcus* sp. BCP1 (DSM44980) strains was performed using 454 sequencing technology (Roche GS FLX Titanium). *R*. *opacus* R7 genome sequencing resulted in one shotgun library made of 312,384 sequence reads and one paired-end library of 380,920 sequence reads. All the reads were assembled into 223 contigs by using Newbler 2.6, with an *N*_50_ length of 184,729 bp and an average genome coverage of 17X. Considering *Rhodococcus* sp. BCP1, the total numbers of sequence reads were 668,686 from one shotgun library and 353,744 from one paired-end library (8-kb inserts). All the reads were assembled into 123 contigs by using Newbler 2.6, with an N_50_ length of 237,787 bp and an average genome coverage of 65X. The preliminary annotation was performed by using the RAST (Rapid Annotation using Subsystem Technology) server [30]. NCBI pipeline was used to annotate genome sequences and to make manual curation. The whole-genome shotgun sequencing projects have been deposited at DDBJ/EMBL/GenBank under the accession numbers CP008947, CP008948, CP008949, CP008950, CP008951, and CP008952 for *R*. *opacus* R7 and AVAE01000000 for *Rhodococcus* sp. BCP1; the Accession Numbers of the main genes discussed in this paper are reported in Table 1.

**Table 1:** Accession Number of the main genes of *R*. *opacus* R7 and *Rhodococcus* sp. BCP1 strains discussed in this paper.

Gene	Homologous protein	Function	Accession Number *R*. *opacus* R7	Accession Number *Rhodococcus* sp. BCP1
*alkB-alkB1*	AlkB	Alkane monooxygenase	AIA09965.1	ADR72654.1
*alkB2*	AlkB2	Alkane monooxygenase	-	KDE11615.1
*rubA*	RubA	Rubredoxin	AIA09966.1	ADR72655.1
*rubB*	RubB	Rubredoxin	AIA09967.1	ADR72656.1
*rubred*	RubRed	Rubredoxin reductase	AIA09968.1	ADR72657.1
*prmA*	PrmA	Methane monooxygenase component A alpha chain	AII03499.1	KDE11344.1
*prmC*	PrmC	Methane monooxygenase component C	AII03498.1	KDE11343.1
*prmB*	PrmB	Methane monooxygenase component A beta chain	AII03497.1	KDE11342.1
*prmD*	PrmD	Methane monooxygenase regulatory protein	AII03496.1	KDE11341.1
*akbA1a*	AkbA1a	Ethylbenzene dioxygenase large subunit	AII11493.1	KDE09919.1
*akbA2a*	AkbA2a	Ethylbenzene dioxygenase small subunit	AII11492.1	KDE09920.1
*akbA3*	AkbA3	Ethylbenzene dioxygenase ferredoxin	CP008952.1	-
*akbA4*	AkbA4	Ferredoxin reductase	AII11490.1	KDE12339.1
*akbB*	AkbB	Dihydrodiol dehydrogenase	AII11489.1	KDE09922.1
*akbC*	AkbC	2,3-Dihydroxybiphenyl 1,2-dioxygenase	AII11058.1	KDE14642.1
*akbD*	AkbD	2-Hydroxy-6-oxo-6-phenylhexa-2,4-dienoate hydrolase	AII11051.1	KDE14641.1
*akbE*	AkbE	2-Hydroxypenta-2,4-dienoate hydratase	AII11050.1	KDE14625.1
*akbF*	AkbF	4-Hydroxy-2-oxovalerate aldolase	AII11049.1	-
*dszA1*	DszA1	Dibenzothiophene desulfurization enzyme	AII08556.1	KDE15059.1
*dszA2*	DszA2	Dibenzothiophene desulfurization enzyme	AII03608.1	KDE15059.1
*dszB*	DszB	Possible ABC sulfonate transporter	AII06125.1	KDE15056.1
*dszC1*	DszC1	Probable dibenzothiophene desulfurization enzyme	AII08748.1	KDE11236.1
*dszC2*	DszC2	Probable dibenzothiophene desulfurization enzyme	AII08273.1	KDE11237.1
*rub1bis*	Rub1bis	Rubredoxin	DQ846881	-
*narR1*	NarR1	Regulator of GntR family	ABH01023.1	KDE09916.1
*narR2*	NarR2	XylR-like regulator protein	ABH01024.1	KDE09917.1
*rub1-rub*	Rub1-Rub	Rubredoxin	ABH01026.1	KDE09915.1
*rub2*	Rub2	Rubredoxin	ABH01027.1	-
*orf7*	Orf7	Sterol-binding domain protein- unknown	ABH01028.1	KDE09918.1
*narAa*	NarAa	Naphthalene dioxygenase large subunit	ABH01029.1	KDE09919.1
*narAb*	NarAb	Naphthalene dioxygenase small subunit	ABH01030.1	KDE09920.1
*narB*	NarB	Cis-naphthalene dihydrodiol dehydrogenase	ABH01031.1	KDE09922.1
*genC*	GenC	Salicylate hydroxylase	AII11448.1; AII10777.1	-
*genB*	GenB	Salicylate CoA syntethase	AII11449.1; AII10778.1	-
*genA*	GenA	Salicylate CoA ligase	AII11450.1; AII10779.1	-
*genH*	GenH	Gentisate dioxygenase	AII11451.1; AII10780.1	KDE14391.1
*genI*	GenI	3-Maleylpyruvate Isomerase	AII11452.1; AII10781.1	KDE14392.1
*genL*	GenL	Unknown function	AII11453.1	KDE14393.1
*bphAa*	BphAa	Biphenyl-2,3-dioxygenase α subunit	AII11493.1	KDE09919.1
*bphAb*	BphAb	Biphenyl-2,3-dioxygenase β subunit	AII11492.1	KDE09920.1
*bphAc*	BphAc	Biphenyl-2,3-dioxygenase, ferredoxin component	AII08472.1	KDE10172.1
*bphAd*	BphAd	Biphenyl-2,3-dioxygenase, reductase	AII11490.1	KDE10578.1
*bphB*	BphB	Cis-2,3-dihydrobiphenyl-2,3- diol dehydrogenase	AII11489.1	KDE09922.1
*bphC*	BphC	2,3-Dihydroxybiphenyl- 1,2-dioxygenase	AII11058.1	KDE14642.1
*akbD*	AkbD	2-Hydroxy-6-oxohepta-2,4- dienoate hydrolase	AII11051.1	KDE11753.1
*bphE*	BphE	2-Oxopent-4-enoate hydratase	AII03622.1	KDE14453.1
*bphF*	BphF	4-Hydroxy-2-oxovalerate aldolase	AII03620.1	KDE14451.1
*bphG*	BphG	Acetaldehyde dehydrogenase	AII03621.1	KDE14452.1
*badI*	BadI	Naphthoate synthase	AII08541.1	KDE15145.1
*badH1*	BadH1	2-Hydroxycyclohexanecarboxyl-CoA dehydrogenase	AII08542.1	KDE15144.1
*badH2*	BadH2	2-Hydroxycyclohexanecarboxyl-CoA dehydrogenase	-	KDE12227.1
*aliA*	AliA	Long-chain-fatty-acid-CoA ligase	AII08543.1	KDE15143.1
*badJ*	BadJ	Acyl-CoA dehydrogenase	AII08544.1	KDE15141.1
*pobA*	PobA	*p*-Hydroxybezoate hydroxylase	AII08627.1	KDE11135.1
*catA1*	CatA1	Catechol 1,2 dioxygenase	AII08813.1	KDE10959.1
*catB1*	CatB1	Muconate cycloisomerase	AII08814.1	KDE10958.1
*catC*	CatC	Muconolactone isomerase	AII08815.1	KDE10957.1
*catA2*	CatA2	Catechol 1,2 dioxygenase	CP008947.1	-
*catB2*	CatB2	Muconate cycloisomerase	AII05696.1	-
*pcaI*	PcaI	Succinyl-CoA 3-ketoacid-coenzyme A transferase subunit B	AII09804.1	KDE10925.1
*pcaJ*	PcaJ	Succinyl-CoA 3-ketoacid-coenzyme A transferase subunit A	AII09803.1	KDE10926.1
*pcaH*	PcaH	Protocatechuate 3,4-dioxygenase beta chain	AII09802.1	KDE10927.1
*pcaG*	PcaG	Protocatechuate 3,4-dioxygenase alpha chain	AII09801.1	KDE10928.1
*pcaB*	PcaB	3-Carboxy-cis,cis-muconate cycloisomerase	AII09800.1	KDE10929.1
*pcaL*	PcaL	3-Oxoadipate enol-lactone hydrolase	AII09799.1	KDE10930.1
*pcaF*	PcaF	Acetyl-CoA acetyltransferase	AII09797.1	KDE10932.1
*paaG*	PaaG	Phenylacetate-CoA oxygenase, PaaG subunit	AII08339.1	-
*paaH*	PaaH	Phenylacetate-CoA oxygenase, PaaH subunit	AII08340.1	-
*paaI*	PaaI	Phenylacetate-CoA oxygenase, PaaI subunit	AII08341.1	-
*paaJ*	PaaJ	Phenylacetate-CoA oxygenase, PaaJ subunit	AII08342.1	-
*paaK*	PaaK	Phenylacetate-CoA oxygenase-reductase, PaaK subunit	AII08343.1	KDE12334.1
*paaF*	PaaF	Phenylacetate-coenzyme A ligase PaaF	AII08344.1	-
*paaE*	PaaE	Acetyl-CoA acetyltransferase	AII08335.1	KDE15146.1
*paaA*	PaaA	Enoyl-CoA hydratase	AII08336.1	-
*paaC*	PaaC	3-Hydroxyacyl-CoA dehydrogenase	AII08337.1	KDE15147.1
*paaB*	PaaB	Enoyl-CoA hydratase	AII08338.1	-
*paaZ*	PaaZ	Aldehyde dehydrogenase	AII08334.1	-
*paaD*	PaaD	Phenylacetic acid degradation protein, thioesterase	AII08332.1	-
*paaL*	PaaL	Acetate permease ActP (cation-acetate symporter)	AII08348.1	KDE10910.1
*hmgA*	HmgA	Homogentisate 1,2-dioxygenase	AII08874.1	KDE10817.1
*fahA*	FahA	Fumarylacetoacetase	AII08876.1	KDE10815.1
*mai*	Mai	Enoyl-CoA hydratase-isomerase	AII08877.1	KDE11332.1
*orf1*	Orf1	Long-chain-fatty-acid-CoA ligase	AII08879.1	KDE10810.1

### Whole-genome alignments

The whole genomes of *Rhodococcus* sp. BCP1 and *R*. *opacus* R7 have been compared with the genomes of *R*. *opacus* PD630, *R*. *opacus* B4 and *R*. *jostii* RHA1 and *R*. *pyridinivorans* SB3094, to find out similar and conserved regions between them and to highlight genes and potential unique functions for each genome under analysis.

For a global alignment and visualization of the comparison of all six genomes, the Mauve tool (2.3 Version) has been used [31] that interactively shows the relative positions of sequence regions (colored blocks) that are found in more than one genome.

A more accurate analysis has been performed using the Last program [32] to obtain local pairwise all-*vs*-all alignments of all chromosome and plasmid sequences of the six genomes. With a post-process overlapping all the regions, the common regions are subtracted from the dataset to obtain a final list of regions that can be considered unique for each genome (present in one genome and not aligned in none of the other five). Only unique regions longer than 300 bp are considered. The genomic diversity in this group of bacteria is also represented in the diagram of Venn. This type of diagram represents the number of predicted protein-coding genes unique or shared amongst the genomes under investigation.

### Metabolic map reconstruction

The preliminary approach to reconstruct metabolic capabilities of *R*. *opacus* R7 and *Rhodococcus* sp. BCP1 was to build a metabolic map using Pathway Tools software version 18.5. It is a comprehensive systems biology software environment for management, analysis, and visualization of integrated collections of genome, pathway, and regulatory data. It supports creation, curation, dissemination and Web-publishing of organism specific databases, called Pathway/Genome Databases (PGDBs), that integrates many types of data. It performs computational inferences, including prediction of metabolic pathways [33]. This software takes as input an annotated genome and compares the encoded proteins with the proteins in the MetaCyc and EcoCyc databases and maps them in metabolic pathways from BioCyc database. MetaCyc consists of a set of 2260 pathways from 2600 different organisms and their constituent enzymes, EcoCyc describes the genome and the biochemical machinery of *Escherichia coli* K-12 MG1655 and BioCyc consists of a collection of 5500 Pathway/Genome Databases (PGDBs). If the enzymes are found in the genome, the pathway is scored as present. The deduced metabolic network tends to have more pathways than actually present in the bacterium, but it supports well as a foundation element for inference [34].

An accurate comparative analysis of the molecular functions has been performed starting from the annotations computed with RAST in which each ORF function was annotated in a manually curated hierarchical taxonomy based on categories, subcategories, subsystems and roles. Categories contain more specific subcategories and subsystems which represent the actual function (a particular cellular basal metabolism or regulation mechanism) of the gene in which it plays a specifc role.

The enzymes of the subsystems represented in the RAST annotation have been mapped to the pathways of the Pathway Tools database. These pathways and the corresponding set of reactions present in the two genomes are compared in order to find differences between them and to quantify, for each pathway, how many components (enzymes) can be found in the genome annotations.

Pathway Tools and RAST have a different annotation system that does not permit to have a complete description of the pathway in terms of presence of their components (reactions or enzymes) in the genome. For this reason the results of the two annotation systems have been integrated together.

### Phylogenomic analyses

In order to compare the two R7 and BCP1 genomes with other *Rhodococcus* genus members, a phylogenetic analysis of 28 different strains was performed. The dataset contains bacteria belonging to the following species: *erythropolis* (2), *rhodochrous* (2), *opacus* (4) (including R7), *wratislaviensis* (2), *jostii* (2), *rhodnii* (1), *pyridinivorans* (2), *ruber* (2) and other less representative species (see results for more details).

We built the concatemer sequences of four marker genes of the 28 strains, that were previously identified as conserved and informative for the bacteria classification: *16S rRNA* gene, *secY* gene, *rpoC* gene and *rpsA* gene [35]. The sequences were aligned with the Muscle sequence alignment program [36] using default parameters and the phylogenetic tree was built with PhyML program [37] that iteratively search for the optimal tree with a Nearest Neighbor Interchange algorithm evaluating Maximum Likelihood probability at each step starting from 5 random generated trees.

### Phenotype Microarray Assays on 811 Compounds

Phenotype Microarray was conducted using Biolog microplates, a tetrazolium-based growth assay developed by Biolog Incorporated (Biolog, Inc., Hayward, CA). R7 and BCP1 strains were assayed on microplates PM1 to PM4, PM9, PM10, PM11 to PM20 testing 811 different substrates such as several carbon sources, nitrogen sources, sulphur and phosphorous sources, different concentrations of ions and osmolites, a wide variety of antibiotics, antiseptics, heavy metals, other inhibitors, and pH stress. Phenotype Microarray technology uses the irreversible reduction of tetrazolium violet to formazan as a reporter of active metabolism [38]. The reduction of the dye causes the formation of a purple colour that is recorded by a charge-coupled-device camera every 15 min and provides quantitative and kinetic information about the response of the microbial cells in PM plates. All the procedures were performed as indicated by the manufacturer. Strains were grown at 30°C on BUG agar (Biolog), and then, each strain was picked with a sterile cotton swab from the agar surface and suspended in 1 mL of sterile mineral medium. Each strain suspension was added to 15 mL of physiological solution until cell density of 79% transmittance (T) was reached on a Biolog turbidimeter [20]. The culture medium was prepared for each type of PM plate: a mineral medium without carbon source was prepared for PM 1, 2 and for PM from 11 to 20; for PM 3 a mineral medium without N source; two kinds of media were prepared for PM 4, one without sulphur source and another without phosphorus source. Inocula for PM9 and PM10 were prepared as described by Viti et al., 2007. After the addition of 1% dye G (vol/vol) to the suspension, 100 μl of the mixture was inoculated into each well of the microplates. All PM microplates were incubated at 30°C in an OmniLog reader, and they were monitored automatically every 15 min. Reads were recorded for 72 h and data were analyzed using OminoLog PM software (release OM_PM_109M), which generated a time course curve for tetrazolium color developed. Each strain was analyzed at least in duplicate and the results were checked for consistency [19]. The growth was also evaluated in each well using optical density measurements at 590 nm (OD_590_) after 72 hours.

### Phenotype Microarray on organic/xenobiotic compounds

The metabolic abilities of R7 and BCP1 strains were also assayed on 41 organic/xenobiotic compounds added as the only carbon and energy source, such as aliphatic hydrocarbons, polycyclic aromatic hydrocarbons, aromatic and compounds belonging to the BTEX group, and naphthenic acids. Strains were grown at 30°C on BUG agar (Biolog), and then, each strain was picked with a sterile cotton swab from the agar surface and suspended in 1 mL of sterile mineral medium. Each strain suspension was added to 15 mL of mineral medium without carbon source until cell density of 79% transmittance was reached on a Biolog turbidimeter. 1% dye G (vol/vol) was added to each suspension before inoculation, while the substrate was supplied to the inoculated wells with a minimum of two wells of distance. The plates were incubated at 30°C in an OmniLog reader and were monitored automatically every 15 min. Readings were recorded for 72 h and the data were analyzed using OminoLog PM software (release OM_PM_109M). Each strain was analyzed at least in duplicate and the results were checked for consistency. The growth was also evaluated using OD_590_ after 72 hours.

### Statistical analysis on Phenotype Microarrays

Activity curves from Phenotype Microarray experiments for both *Rhodococcus* sp. BCP1 and *R*. *opacus* R7 have been analyzed. A clustering analysis has been performed with the DuctApe program [39] to group together the curves using nine characteristic curve parameters and three sigmoid-like models. Errors in the fitting confirmed that a direct calculation of activity area in this case is more accurate that a model-estimated activity based on not completely developed growth curves.

Activity values are reported for all the substrates into the standard Biolog Plate PM (1, 2, 3, 4, 9,10, 11, 12, 13, 14, 15, 16, 17, 18, 19, 20) and for the custom set of xenobiotics as described before. In this analysis the activities are scattered in a 2D plot; in the same plot three diagonal lines representing the mean (μ_A_), standard deviation (σ_A_) and double standard deviation (2σ_A_) of the difference activity (ΔA) of the two genomes are overlapped. In this way it is easy to highlight regions of specificity for the two genomes on the tails of the gaussian distribution: *R*. *opacus* R7 specific compounds are in regions where ΔA <-σ_A_ and *Rhodococcus* sp. BCP1 specific compounds are on the other extreme points: ΔA > +σ_A_.

For both *R*. *opacus* R7 and *Rhodococcus* sp. BCP1 strains the PM activities obtained with different compounds were associated with enzymes (EC code) gathered from the Kyoto Encyclopedia of Genes and Genomes (KEGG) database [40, 41]. Enzyme not available are labeled as unknown.

The activities reported in the heat maps are normalized in a scale from low activity (red color) to high activity (green color).

### Identification of genetic aspects for xenobiotic degradation pathways

The degradation pathways of some xenobiotic compounds and putative gene clusters were predicted in *R*. *opacus* R7 and *Rhodococcus* sp. BCP1 strains by manual alignment analyses of gene clusters previously identified or extracted from sequences of reference strains reported in databases. The identified gene clusters were partially described and compared with those from other *Rhodococcus* strains reported in literature. The NCBI pipeline and RAST server were used for genome predictions and comparisons.

## Results

### Genome features of *Rhodococcus opacus* R7 and *Rhodococcus* sp. BCP1 strains

The genomes of *Rhodococcus opacus* R7 and *Rhodococcus* sp. BCP1 strains were completely sequenced [22, 23]. R7 genome was arranged in a chromosome of 8,466,345 bp and in 5 plasmids (pDG1 of 656,443 bp, pDG2 of 426,388 bp, pDG3 of 352,342 bp, pDG4 of 191,359 bp, and pDG5 of 25,175 bp), giving a total genome size of 10.1 Mb with a G-C content of 66.0%. RAST analysis of R7 genome sequence identified a total of 9,602 open reading frames (ORFs) and 62 RNAs genes (9 rRNAs and 53 tRNAs). *Rhodococcus* sp. BCP1 genome consisted of one chromosome of 6,008,321 and two plasmids (pBMC1 and pBMC2 of 120,373 bp and 103,29 bp, respectively), giving a total genome size of 6.2 Mb with a G-C content of 70.4%. A total of 6,206 open reading frames (ORFs) and 58 RNAs genes (8 rRNAs and 50 tRNAs) were annotated.

By RAST analysis, the annotated ORFs of *R*. *opacus* R7 and *Rhodococcus* sp. BCP1 were classified into categories as reported in Table 2. As a result of this classification, the number of R7 ORFs was double than BCP1 for each category. Moreover, 457 and 425 subsystems were found in R7 and in BCP1 strains, respectively. In R7, subsystem categories representing the metabolism of carbohydrates, amino acids and cofactors, vitamins, prosthetic groups, or pigments, are the most abundant, and they account for 1,236, 995, or 665 proteins, respectively. A total of 745 ORFs are involved in metabolism of fatty acids, lipids, and isoprenoids, while 267 ORFs participate in metabolism of aromatic compounds. A total of 129 oxygenases/hydroxylases amongst the 134 annotated are predicted to catalyze the oxidation of organic compounds with industrial and environmental relevance, such as linear alkanes, cyclic ketones, aromatic compounds (e.g., benzoate, catechol, gentisate, salycilate, and byphenil), aminopolycarboxilic acids, nitroalkanes, and phenylalkanoic acids. Forty-five ORFs encode cytochrome P450 monooxygenases that catalyze regio- and stereospecific oxidation of a vast number of substrates. In BCP1 subsystem categories representing the metabolism of carbohydrates, amino acids and cofactors, vitamins, prosthetic groups, or pigments are the most abundant, and they account for 623, 565, or 429 proteins, respectively. In BCP1, 415 ORFs are involved in the metabolism of fatty acids, lipids, and isoprenoids, while 135 ORFs participate in the metabolism of aromatic compounds. Seventy-three oxygenases/hydroxylases amongst the 134 annotated genes are predicted to catalyze the oxidation of organic compounds with industrial and environmental relevance, such as linear alkanes, cyclic ketones, aromatic compounds (e.g., benzoate, catechol, gentisate, salicylate), aminopolycarboxylic acids, nitroalkanes, and phenylalkanoic acids. Twenty-six ORFs encode cytochrome P450 monooxygenases that catalyze regio- and stereospecific oxidation of a large number of substrates.

**Table 2:** Number of functions in each of the RAST category.

Category	*R*. *opacus* R7	*Rhodococcus* sp. BCP1
**Cofactors, Vitamins, Prosthetic Groups, Pigments**	665	429
**Cell Wall and Capsule**	111	80
**Virulence, Disease and Defense**	124	104
**Potassium metabolism**	17	24
**Miscellaneous**	115	81
**Phages, Prophages,Transposable elements, Plasmids**	0	7
**Membrane Transport**	108	92
**Iron acquisition and metabolism**	16	16
**RNA metabolism**	106	87
**Nucleosides and Nucleotides**	156	102
**Protein Metabolism**	257	230
**Cell Division and Cell Cycle**	35	27
**Motility and Chemotaxis**	5	4
**Regulation and Cell signaling**	75	51
**Secondary Metabolism**	13	7
**DNA metabolism**	103	124
**Fatty Acids, Lipids and Isoprenoids**	745	415
**Nitrogen Metabolism**	71	38
**Dormancy and Sporulation**	3	3
**Respiration**	221	158
**Stress Response**	162	138
**Metabolism of Aromatic Compounds**	267	128
**Amino Acids and Derivatives**	995	565
**Sulphur Metabolism**	126	58
**Phosphorus Metabolism**	35	32
**Carbohydrates**	1236	623
**Total**	**5767**	**3623**

### Metabolic network reconstruction

The metabolic network reconstruction of *R*. *opacus* R7 and *Rhodococcus* sp. BCP1 genomes was performed using pathway tools software 18.5. The metabolic reconstruction resulted in a model containing 1735 metabolic reactions for both R7 and BCP1 strains. As a result, 322 (R7) and 274 (BCP1) pathways, 1836 (R7) and 1660 (BCP1) enzymatic reactions, 10 (R7) and 8 (BCP1) transport reactions, 2023 (R7) and 1436 (BCP1) enzymes, 13 (R7) and 9 (BCP1) transporters were assigned by this software analysis. The identified pathways were divided in classes, as described in Table 3 and categorized into the RAST categories (Table 2). RAST annotation assigned 26 functional categories for both genomes, 457 subsystems and 6089 functional roles for *R*. *opacus* R7 and 425 subsystems and 3703 functional roles for *Rhodococcus* sp. BCP1. The number of functions and their distribution reflected the differences in size of the two genomes (10 Mb for *R*. *opacus* R7 and 6 Mb for *Rhodococcus* sp. BCP1).

**Table 3:** Numbers of metabolic pathways deriving from metabolic reconstruction of *R*. *opacus* R7 and *Rhodococcus* sp. BCP1 genomes.

Pathway Class	Number of Pathways (R7)	Number of Pathways (BCP1)
**activation/inactivation/interconversion**	3	2
**biosynthesis**	220	217
**degradation/utilization/assimilation**	171	115
**detoxification**	5	4
**generation of precursor metabolites and energy**	25	21
**metabolic clusters**	8	8
**superpathways**	82	72
**TOT**	**322**	**274**

Although the integration of the information of pathway tools and RAST allowed to obtain an improved pathway reconstruction for both the genomes, only around 50% of the pathways have all their enzymes represented in the annotation. This percentage increases up to 70% when pathways, represented by all the enzymes except one, are considered.

### Whole-genome comparison

Whole genome sequence comparison of *R*. *opacus* R7 and *Rhodococcus* sp. BCP1 with a set of four other reference genomes (*R*. *jostii* RHA1, *R*. *opacus* PD630, *R*. *opacus* B4, *R*. *pyridinivorans* SB3094) was performed using the Mauve program, in the Mauve 2.3 Version and using the Last Software (S1 Fig). Aligned segments of pair of genomes have been collected in a dataset, filtered to remove regions <300 bp in length and grouped by genome. Similarity score between one reference genome and each other genome has been calculated considering the total amount of bases shared between the reference genome and each other genome under analysis.

Results show that *Rhodococcus* sp. BCP1 and *R*. *opacus* R7 share a total of 81% (BCP1 as reference) or 52% (R7 as reference) of similarity, calculated on all chromosomes and plasmids (Table 4). Considering the genome comparison amongst the *R*. *opacus* members, they share a range of similarity of 70%-90% percentage. A lower percentage of similarity resulted from the comparison of either *R*. *pyridinivorans* SB3094 or *Rhodococcus* sp. BCP1 with each *R*. *opacus* strain (around 50% and 40%, respectively). The analysis of relative positions of the sequences shared amongst the genomes (chromosome and plasmids) shows that they mainly do not maintain the same order (Table 5).

**Table 4:** Similarity scores for *Rhodococcus* genomes under analysis. Values in the matrix represent the percent of bases shared in regions longer than 300 bp.

	*R*. sp. BCP1	*R*. *opacus* R7	*R*. *jostii* RHA1	*R*. *opacus* PD630	*R*. *opacus* B4	*R*. *pyridinivorans* SB3094
***R*. sp. BCP1**	-	81.27%	81.09%	65.43%	73.66%	65.07%
***R*. *opacus* R7**	51.67%	-	83.74%	71.39%	74.69%	44.67%
***R*. *jostii* RHA1**	50.90%	82.79%	-	68.61%	73.80%	44.48%
***R*. *opacus* PD630**	55.27%	91.54%	89.30%	-	80.25%	48.78%
***R*. *opacus* B4**	56.26%	89.91%	88.17%	73.15%	-	48.93%
***R*. *pyridinivorans* SB3094**	71.52%	74.36%	74.98%	60.36%	70.00%	-

**Table 5:** Numbers of similar regions that maintain the same order in each pair of genomes respect the total number of similar regions in R7 and BCP1 strains.

	*R*. sp. BCP1	*R*. *opacus* R7	*R*. *jostii* RHA1	*R*. *opacus* PD630	*R*. *opacus* B4	*R*. *pyridinivorans* SB3094
***R*. sp. BCP1**	-	286/25919 (1.10%)	475/23532 (2.02%)	400/17911 (2.23%)	481/22904 (2.10%)	642/12397 (5.18%)
***R*. *opacus* R7**	-	-	123/40390 (0.30%)	121/30862 (0.39%)	129/39122 (0.33%)	261/21603 (1.21%)
***R*. *jostii* RHA1**	-	-	-	220/26720 (0.82%)	233/33364 (0.70%)	553/20422 (2.71%)
***R*. *opacus* PD630**	-	-	-	-	195/25465 (0.77%)	462/15651 (2.95%)
***R*. *opacus* B4**	-	-	-	-	-	546/19708 (2.77%)
***R*. *pyridinivorans* SB3094**	-	-	-	-	-	-

**Fig 1:**
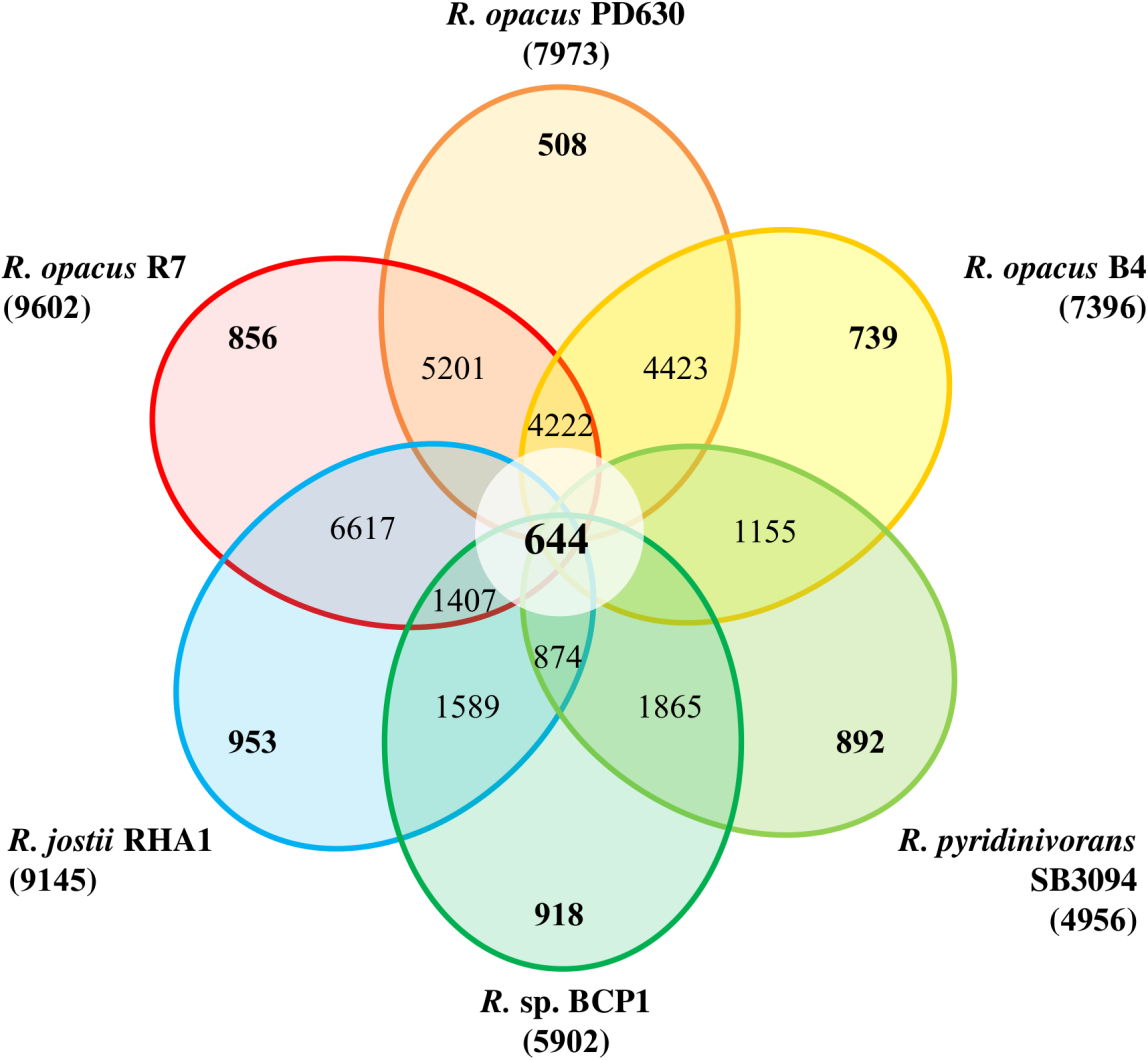
Venn Diagram. Genomic comparison of *R*. *opacus* R7 and *Rhodococcus* sp. BCP1 with other *Rhodococcus* strains, including *R*. *jostii* RHA1, *R*. *opacus* PD630, *R*. *opacus* B4, *R*. *pyridinivorans* SB4094. Each strain is represented by a colored oval. Number of predicted protein coding genes (CDSs) shared by all strains (i.e., the core genome) is in the centre. Overlapping regions show the number of CDSs conserved only within the specified genomes. Numbers in non-overlapping portions of each oval show the number of CDSs unique to each strain. The total number of protein coding genes within each genome is listed below the strain name.

The percentage of the regions of each *Rhodococcus* strain shared amongst all the genomes under analysis, ranged between 37% and 50% values, indicating a high level of genetic variability amongst *Rhodococcus* genus strains. The genomic diversity in this group of bacteria is represented in the diagram of Venn shown in Fig 1. The core genome identified for the *Rhodococcus* strains under analysis contains 644 predicted protein coding genes, that represent around the 50% of the predicted proteome of each strain. The most shared regions identified in *Rhodococcus* sp. BCP1 and *R*. *opacus* R7 are located on the chromosomes; this result suggests that these regions could be characteristic of the *Rhodococcus* genus.

### Unique and non-unique genomes features

Genomic comparison of *R*. *opacus* R7 and *Rhodococcus* sp. BCP1 with four strains belonging to *Rhodococcus* genus allowed to identify unique genomic regions in their genomes (Table 6) (S1 Table). The genomic divergence was investigated in the two genomes and in the complete and well characterized sequenced genomes of *R*. *jostii* RHA1, *R*. *opacus* PD630, *R*. *opacus* B4 and *R*. *pyridinivorans* SB3094. The comparison showed that they are highly conserved except for the unique regions (longer than 300 bp); the majority of them including annotated genes.

**Table 6:** Unique regions larger than 4 kb identified in R7 and BCP1 strains.

	*R*. *opacus* R7			*Rhodococcus* sp. BCP1		
Region n.	Predicted key functions	Size (bp)	Unique gene number	Predicted key functions	Size (bp)	Unique gene number
1	Amino acids biosynthesis	4373	3	Carotenoid biosynthesis	4962	5
2	Aromatic compounds degradation	5323	4	Unknown function	7992	7
3	Transcriptional regulators	4004	4	Amino acids metabolism	7102	5
4	Phage region	5208	2	Cell division	6351	6
5	Unknown function	8845	2	Cell division	12430	10
6	Unknown function	7095	6	Metal transport	4332	4
7	Phage region	6060	6	Unknown function	4523	4
8	Polysaccharides biosynthesis	7648	3	Cell wall biogenesis	6427	3
9	Amino acids biosynthesis	4256	2	Carbon and energy storage	4593	3
10	Unknown function	4289	2	Fatty acid metabolism	4596	4
11	Unknown function	5176	4	Unknown function	5575	5
12	Amino Acids biosynthesis	4051	3	Mobile element	12922	11
13	Phosphonate metabolism	4131	4	Aromatic compound degradation; Fatty acid degradation	4047	4
14	Unknown function	5775	4	Cell wall biogenesis; O-antigen	18068	17
15	,Monosaccharides metabolism	5707	3	Phospholipid biosynthesis	6067	7
16	Fatty acids metabolism	4195	2	Lactate utilization	4416	2
17	Antibiotic resistance; Folate Biosynthesis	4702	6	Amino acids metabolism; Fatty Acid metabolism; Phage region	30497	44
18	Monosaccharides metabolism	7569	6	Phage region	6871	12
19	Aromatic compounds degradation	4104	7	Organic acids metabolism	4815	2
20	Amino acids biosynthesis	4662	4	Fatty acids biosynthesis; DNA replication	6586	4
21	Membrane transport systems	7072	5	Mobile element	5229	6
22	Amino acids biosynthesis	4935	4	Peptidoglycan Biosynthesis	9655	4
23	Unknown function	6723	6	Mobile element	16590	7
24	Unknown function	6764	4	DNA/RNA topology; ATP/GTP binding protein	7210	3
25	Phage region	20558	16	Lipoprotein biosynthesis	4899	3
26	DNA recombination/repair	5651	2	Fatty acids biosyntehsis	4437	5
27	Unknown function	7525	1	Phage region	9451	7
28	Unknown function	6665	9	Mobile element	5618	2
29	Carbohydrates Metabolism	4823	5	PAH Hydrocarbons degradation	4556	3
30	Phage region	14443	16	Unknown function	5551	6
31	Antibiotic resistance	4233	4			
32	ABC Transporters	6368	5			
33	Amino acids biosynthesis	4221	4			
34	Unknown function	4522	4			
35	Amino acids metabolism	5411	4			
36	Aliphatic hydrocarbon degradation	5491	3			
37	Flavonoid metabolism	4180	4			
38	Carbohydrates metabolism; Aromatic compounds degradation	5293	7			
39	Unknown function	6728	9			
40	Aliphatic hydrocarbon degradation	4132	1			
41	Unknown function	4476	6			
42	Stress response	4006	4			
43	Unknown function	9977	13			
44	Unknown function	4324	1			
45	Oxidoreductase	4899	6			
46	Mobile element	4390	1			
47	Serine/threonine protein kinase	7470	10			
48	Unknown function	7688	4			
49	Unknown function; Phage region	8499	9			

The six strains have different amount of unique regions in the entire genomes as reported in Table 7. In particular, *Rhodococcus* sp. BCP1 showed the highest content of unique regions (16.3%) followed by *R*. *jostii* RHA1 (11.41%) and *R*. *opacus* R7 (11.32%). Within the unique regions, a percentage of 30% and 40% of the annotated genes were predicted to code for hypothetical proteins in BCP1 and R7, respectively. Moreover, around 30% of these regions was found to be non-coding sequence. Genes encoding several enzymatic classes predicted to be involved in the organic compounds metabolism were identified in unique regions in *R*. *opacus* R7 and *Rhodococcus* sp. BCP1, including oxygenases, hydroxylases, dehydrogenases, hydrolases, oxidoreductases, ligases, isomerases, aldolases, ion-transporting ATPases and cytochromes P450 (S2 Table). The presence of these genes suggests different or specific metabolic capabilities of these *Rhodococcus* strains. In particular, considering the oxygenase/hydroxylase class, BCP1 unique regions contain a whole gene cluster encoding the soluble di-iron monooxygenase (*smo*) involved in the short-chain *n*-alkanes metabolism [28], two cyclohexanone monooxygenases, one cytochrome P450, and several oxygenases involved in the aromatic compound metabolism (e.g. 2,3-dihydroxyphenyl 1,2-dioxygenase, 4-hydroxyphenyl acetate 3-monooxygenase, catechol 1,2-dioxygenase and biphenyl dioxygenase beta subunit). Conversely, in *R*. *opacus* R7 unique regions, two alkanal monooxygenases and one alkane-1-monooxygenase (annotated in RAST as alkanal monooxygenase), one cyclohexanone monooxygenase, and one pentachlorophenol monooxygenase were found. Other genes encoding several enzymatic classes are also located in unique regions such as kinases, peptidases, reductases, permeases, synthetases, esterases, cyclases, transcriptional regulators and membrane proteins. Interestingly, the presence of mobile elements (transposases, phage and mobile element proteins) was found, suggesting the occurrence of recombination events. Other important features of aerobic microorganisms able to degrade a wide range of organic compounds are the abilities to counteract stress conditions. Concerning this aspect, several genes coding for proteins involved in resistance to oxidative stress, metals and antibiotics were found in these regions (Table 6). The unique regions contained some heavy metal-resistance genes (i.e.lead-cadmium-zinc-mercury transporting ATPases, cobalt-zinc-cadmium resistance protein, metal-binding enzymes), stress-resistence genes (i.e.catalases, universal stress proteins, chaperons), and antibiotic-resistance genes (i.e.drug/metabolites transporters, beta-lactamase related proteins, penicillin-binding proteins).

**Table 7:** Uniqueness characteristics of the six compared *Rhodococcus* spp. strains.

Uniqueness characteristics	*R*. *opacus* R7	*R*. sp. BCP1	*R*. *jostii* RHA1	*R*. *opacus* PD630	*R*. *opacus* B4	*R*. *pyridinivorans* SB3094
**Lenght of unique regions (bp)**	1,145,011	1,015,869	1,106,630	650,025	912,915	545,841
**Unique regions (%)**	11.32	16.3	11.41	7.1	10.33	10.44
**ORFs in unique regions**	1202	1266	1247	575	1058	1197
**ORFs of hypothetical proteins in unique regions (%)**	39.77	30.88	48.44	42.78	41.30	46.45
**Not Annotated (%)**	29.03	29.46	24.78	32.52	27.32	27.57

### Taxonomic classification of *Rhodococcus opacus* R7 and *Rhodoccocus* sp. BCP1

**Fig 2:**
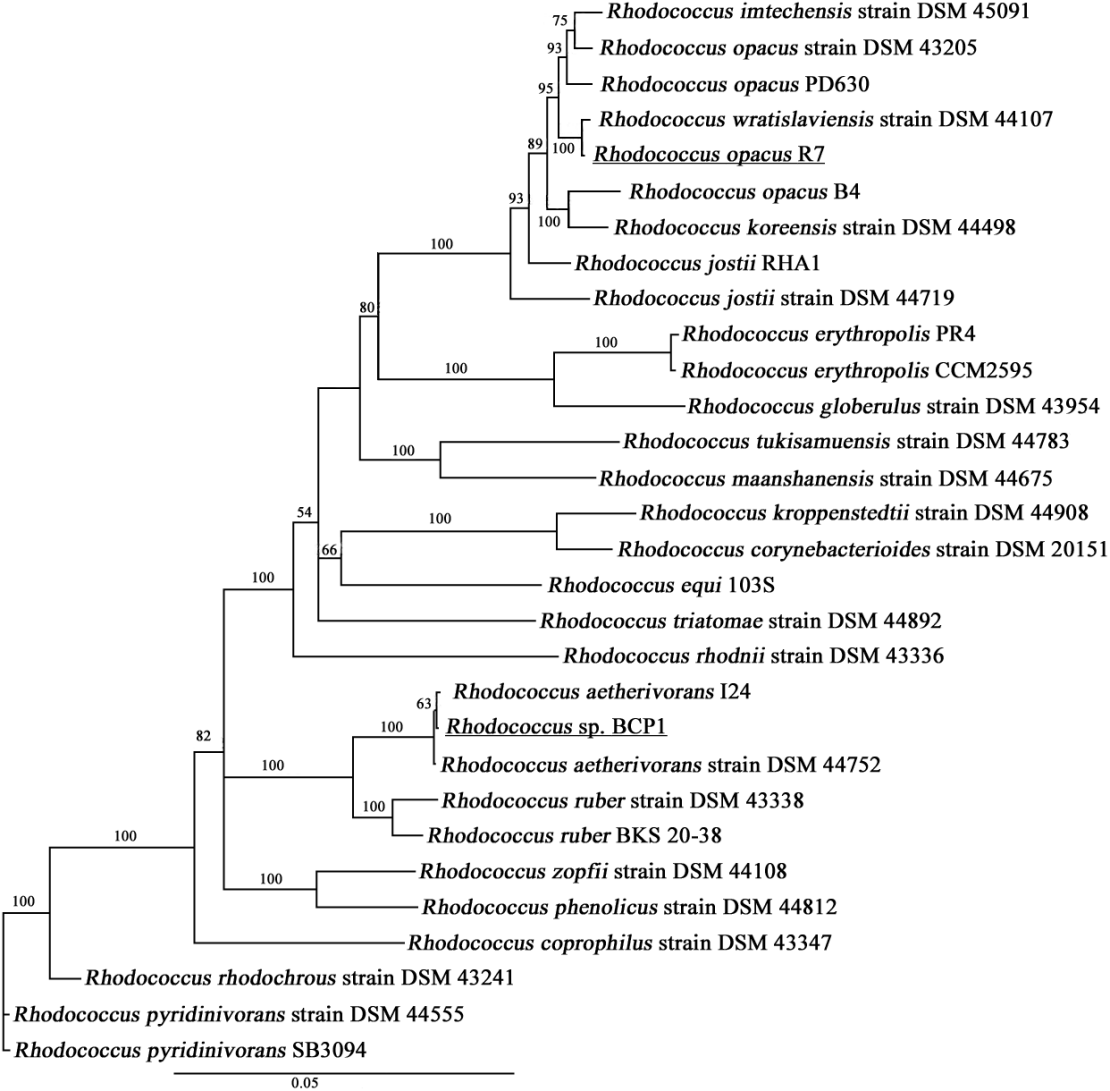
PhylogeneticTree. Phylogenetic analysis of *R*. *opacus* R7 and *Rhodococcus* sp. BCP1 based on sequence alignments with reference strains of *Rhodococcus* genus. The tree was constructed based on concatemer sequences of four marker genes of the 28 strains: *16S rRNA* gene, *secY* gene, *rpoC* gene and *rpsA* gene.

To develop a framework for resolving the phylogeny of the two *Rhodococcus* strains, we constructed a multi-locus sequence analysis (MLSA) maximum likelihood (ML) tree based on four marker genes (*16SrRNA*, *secY*, *rpoC* and *rpsA*) that were previously identified as conserved and informative for the bacteria classification [35]. The phylogenetic tree based on sequence alignments with reference strains of *Rhodococcus* genus show the distinctly defined phylogenetic positions of *Rhodococcus* sp. BCP1 and *R*. *opacus* R7 (Fig 2). This is the first taxonomical study of *R*. *opacus* R7 that correlates R7 strain to *Rhodococcus opacus* and *Rhodococcus wratislaviensis* species in a clade that also includes *R*. *jostii* RHA1. *Rhodococcus* sp. BCP1 clusters with *R*. *aetherivorans* strains in a clade including also *R*. *ruber* species. These results on BCP1 are in line with previous phylogenetic analyses based on *Rhodococcus* 16S rDNA genes and 1-alkane monooxygenase (AlkB) protein sequences reported by Táncsics et al., 2015 [42]. In conclusion, *Rhodococcus* sp. BCP1 can be taxonomically related to *R*. *aetherivorans* species. The importance of this taxonomic definition is related to the fact that BCP1 strain genome is the first complete genome available in database for *R*. *aetherivorans* species.

### Phenotype Microarray analysis of nutritional metabolism by *Rhodococcus opacus* R7 and *Rhodococcus* sp. BCP1

In order to acquire a global understanding of the metabolic capabilities of *Rhodococcus* sp. BCP1 and *R*. *opacus* R7 cellular processes, we used phenotype microarrays (PM) analysis. Using PM plates 1 to 4, 379 different compounds were tested, including 190 different carbon (C) sources, 95 nitrogen (N) sources, 59 phosphorus (P) sources, 35 sulphur (S) sources. By means of the PM plates 9 to 20, the tolerance/sensitivity to different osmolytes, pH conditions and other chemicals (i.e. antibiotics, metals, preservatives etc.) was assayed. In this work, we shed light on the metabolic features of the two strains. In some cases, these metabolic properties were related to the genetic traits present in their genomes [22, 23].

**Fig 3:**
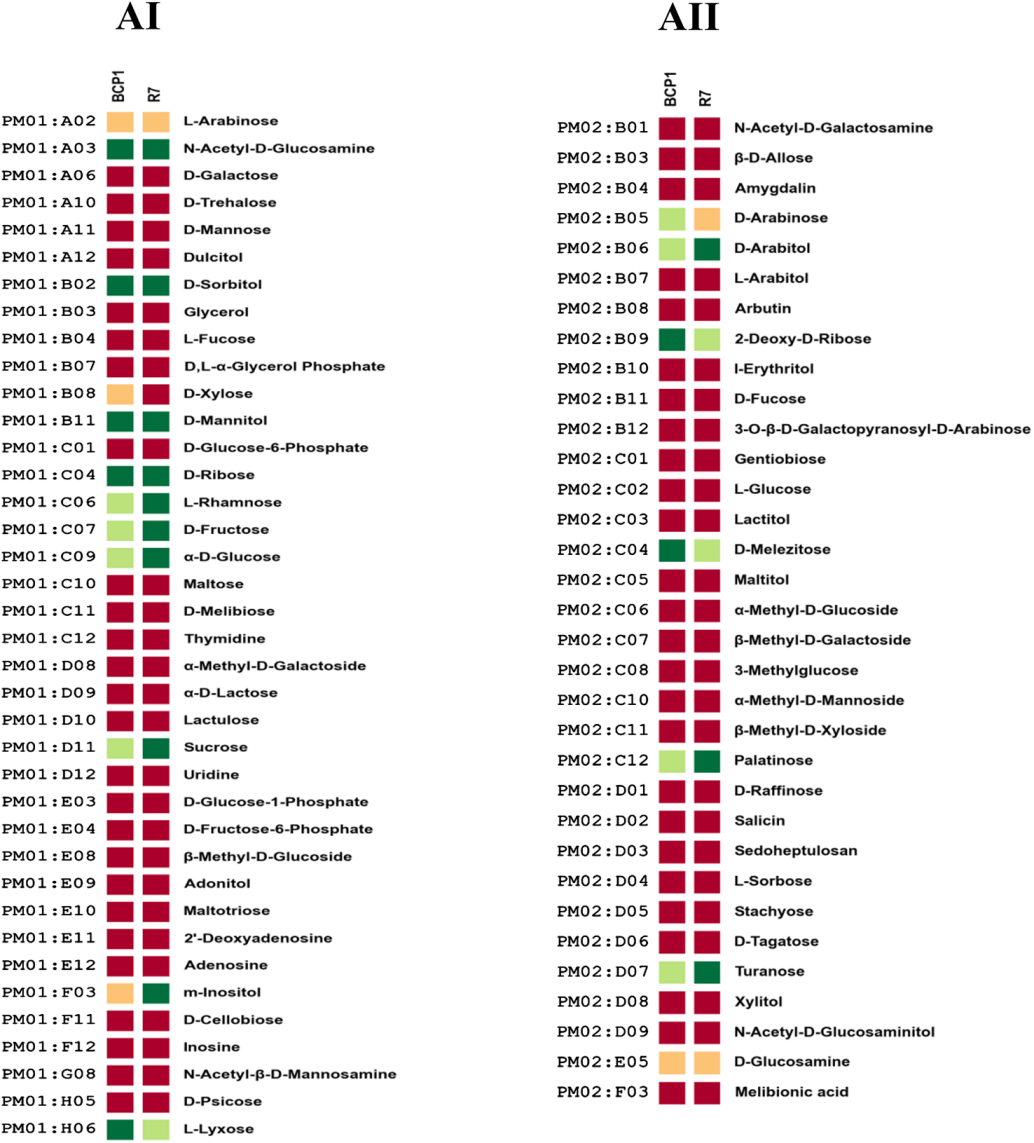
Phenotype Microarray PM with different carbohydrates as carbon sources. Metabolic differences among *R*. *opacus* R7 and *Rhodococcus* sp. BCP1 in presence of carbohydrates (AI, AII). Based on activity values of phenotype microarray analysis, threshold values were established for every plates. Determined thresholds were high (green), upper middle (light green), lower middle (orange) and low (red) for high, upper middle, lower middle and low activity, respectively.

**Fig 4:**
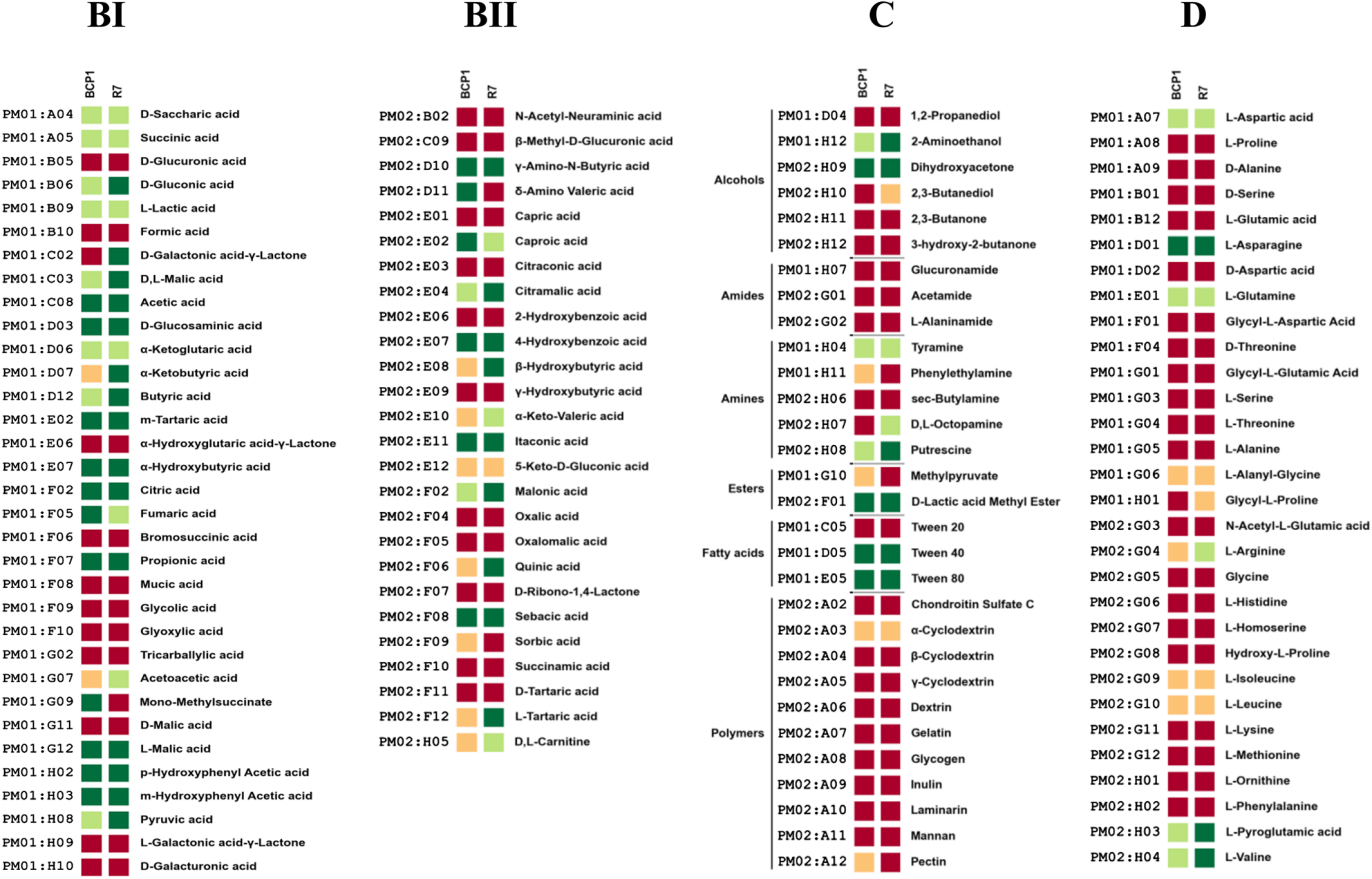
Phenotype Microarray PM with different carboxylic acids-alcohols, amines, amides, esters, fatty acids and polymers-amino acids as carbon sources. Metabolic differences among *R*. *opacus* R7 and *Rhodococcus* sp. BCP1 in presence of carboxylic acids (BI, BII), of alcohols, amines, amides, esters, fatty acids, polymers (C), and amino acids (D). Based on activity values of phenotype microarray analysis, threshold values were established for every plates. Determined thresholds were high (green), upper middle (light green), lower middle (orange) and low (red) for high, upper middle, lower middle and low activity, respectively.

The 190 carbon sources were divided in different chemical categories, including carbohydrates, carboxylic acids, nitrogen containing compounds, alcohols, amides, amines, esters, fatty acids, polymers (S3 Table). R7 and BCP1 strains were metabolically active on the following monosaccharides: α-D-glucose, D-fructose, D-ribose, L-rhamnose, L-lyxose and 2-deoxy-D-ribose. Both the strains could oxidize the three disaccharides composed by glucose and fructose monomers (sucrose, palatinose and turanose) as well as the trisaccharide D-melezitose (formed by D-turanose and D-glucose). They showed high metabolic activity on the sugar alcohols D-sorbitol, D-mannitol and D-arabitol. Amongst the carbon sources utilized, R7 generally showed higher activities compared to BCP1 (69% of the carbon sources utilized by R7 gave high activity level *vs* 41% for BCP1) (Fig 3 Panel AI, AII). Both the strains could utilize a wide range of carboxylic acids (Fig 4 Panel BI, BII). Several short-chain carboxylate compounds (C2-C6) were oxidized by R7 and BCP1 strains including acetic and propionic acids as well as other common intermediates in central metabolism such as pyruvic, succinic, and citric acids. They both oxidized and grew on gluconic acid that is degraded by the pentose phosphate pathway. Carboxylate metabolism also included the C10 dicarboxylate sebacic acid whose metabolism involves the β-oxidation pathway. β-oxidation could also explain the activity on Tween 40 and Tween 80 compounds by BCP1 and R7 (Fig 4 Panel C). Tween 20 did not support growth of the two *Rhodococcus* spp. probably because of the toxicity resulting from the hydrolytic process of this compound [16]. Interestingly, the utilization of Tween compounds is considered diagnostic of the substrate specificity of bacteria toward the use of hydrocarbons as a source of carbon and energy [43]. The complexity of fatty acids β-oxidation pathway present in BCP1 and R7 genomes is consistent with the number of genes coding for each of the oxidation steps (S4 Table).

By RAST annotation analysis, the genes predicted to be involved in central metabolic pathways for carbohydrates and organic acids utilization were more than double for R7 compared to BCP1 (Table 2) (S5 Table). Some of the metabolic differences between the two strains in terms of activity level can be therefore related to the presence of genes in R7 coding for specific functions that lack in BCP1 genome. In R7 genome, genes coding for L-rhamnose ABC-transporter components (EC 3.6.3.25), a L-rhamnose mutarotase (EC 5.1.3.322), a L-rhamnose isomerase (EC 5.3.1.14), a L-arabinose isomerase (EC 5.3.1.4), a L-rhamnose 1-phosphate aldolase (EC 4.1.2.-), a rhamnulokinase (EC 2.7.1.5) and a LacI family transcriptional regulator (EC 3.6.3.17) are clustered in one genomic region and can internalize, metabolize and regulate the rhamnose utilization in this strain. Three 2-dehydro-3-deoxygluconate kinases (EC 2.7.1.45) can phosphorylate and activate the 2-dehydro-3-deoxy-D-gluconate that can be converted to D-glyceraldehyde-3-phosphate to be introduced in glycolysis pathway. The activity of a 2-dehydro-3-deoxygluconate kinase can also be associated to a gluconate dehydratase (EC 4.2.1.39) for the conversion of gluconic acid and its derivatives (such as D-glucono-1,5-lactone and 2-2-dehydro-3-deoxy-D-gluconate) into intermediates of the Entner–Doudoroff pathway. A sucrose-6-phosphate hydrolase (EC 3.2.1.26) that is able to break the sucrose 6-phosphate in α-D-glucose-6-phosphate and β-D-fructose is clustered with genes coding for several sugar-transport ABC transporters and one gene coding for an α-glucosidase (EC 3.2.1.20) that hydrolyses (1–4)-α-glucosidic linkages releasing D-glucose. Two ribokinases in R7 (EC 2.7.1.15) are predicited to catalyze the transfer of a phosphate to ribose or to 2-deoxy-D-ribose. Finally, a N-acetylglucosamine phosphotransferase (PTS) system (EC 2.7.1.69) and a N-acetylglucosamine-6-phosphate deacetylase (EC 3.5.1.25) can be related to the higher metabolic acitivity shown by R7 on N-acetylglucosamine.

Both R7 and BCP1 strains show metabolic activity on aromatic and hydroxylated aromatic compounds such as *p*-hydroxy benzoic acid, *m- p-* hydroxyphenyl acetic acid. R7 genome analysis evidenced a gene coding for a *p*-hydroxybenzoate hydroxylase (EC 1.14.13.2) located in a chromosomal region showing the same genetic organization of a chromosomal region of *R*. *jostii* RHA1. This gene is flanked by genes coding for a benzoate transporter, two transcriptional regulators belonging to IclR family and TetR family, and 1-alkane monooxygenase (AlkB) gene cluster. The *p*-hydroxybenzoate hydroxylase oxidizes the aromatic ring to form protocatechuate. All the genes coding for enzymes involved in protocatechuate metabolism are present in R7. In BCP1 the gene coding for the *p*-hydroxybenzoate hydroxylase is present but is located in a region not conserved amongst bacteria and not including a benzoate transporter gene. An IclR transcriptional regulator is also associated to this gene in BCP1. R7 and BCP1 genomes present six and four genes respectively coding for *p*-hydroxyphenylacetate 3-monooxygenase (EC 1.14.14.9); only R7 possesses also a gene coding for the *p*-hydroxyphenylacetate 3-monooxygenase reductase component associated with genes coding for an aromatic ring oxygenase and a phenylacetate-CoA ligase. Amongst the amino acids, L-aspartic acid, L-asparagine, L-glutamine, L-pyroglutamic acid and L-valine could be utilized as carbon sources by both strains; while only R7 showed high metabolic activity on L-arginine (Fig 4 Panel D). The amines putrescine and tyramine could be utilized by R7 and BCP1 for growth. Both the R7 and BCP1 strains showed a slight activity on the polymer α-ciclodextrin (Fig 4 Panel C). This feature can be correlated with the presence of five and two glucoamylases (EC 3.2.1.3) in BCP1 and R7 genomes, respectively. Four out of the five glucoamylases genes of BCP1 did not show any similarity with genes in *R jostii* RHA1. In R7, both the glucoamylase genes presented flanking regions homologous to those found in RHA1. Both R7 and BCP1 strains could not utilize any amide (glucuronamide, acetamide, L-alaninamide) as carbon source.

**Fig 5:**
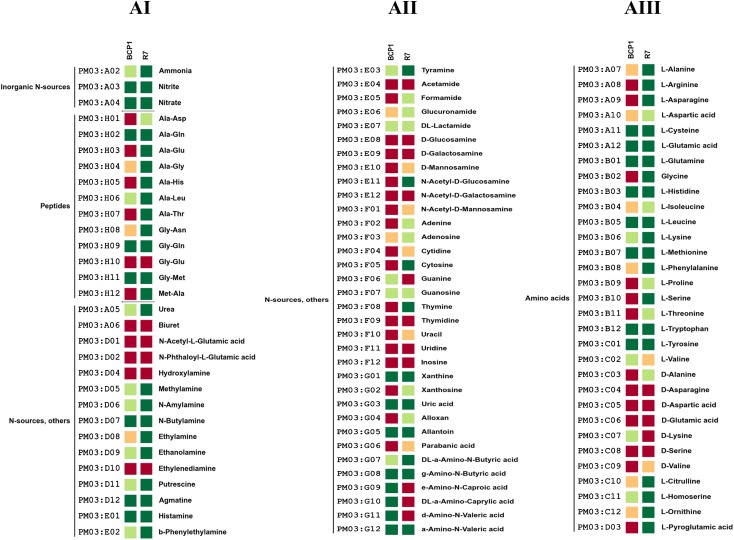
Phenotype Microarray PM with different Nitrogen sources. Metabolic differences among *R*. *opacus* R7 and *Rhodococcus* sp. BCP1 in presence of different Nitrogen sources (AI, AII, AIII). Based on activity values of phenotype microarray analysis, threshold values were established for every plates. Determined thresholds were high (green), upper middle (light green), lower middle (orange) and low (red) for high, upper middle, lower middle and low activity, respectively.

Using the PM3 plate, the two *Rhodococcus* strains were tested for their ability to grow on 95 different nitrogen sources. R7 was able to utilize 74 out of 95 nitrogen sources, while BCP1 could grow only on 52 of the tested nitrogen compounds (Fig 5 Panel AI, AII, AIII). In particular, amongst the utilized N sources, R7 and BCP1 showed high metabolic activity on 50 and 24 compounds, respectively. Twenty-six N sources were specific for R7 strain growth, while 4 nitrogen sources were used by BCP1 and did not support the growth of R7. The nitrogen sources selective for BCP1 growth were D-lysine, DL-α-amino-caprylic acid, δ-amino-N-valeric acid, ε-amino-N-caproic acid. Interestingly, δ-amino-N-valeric acid was also a carbon source utilized by BCP1 but not by R7, suggesting the presence in BCP1 of transport and/or metabolic system specific for the utilization of this amino fatty acid compound. RAST annotation reported a number of genes predicted to be involved in amino acid metabolism that was double in R7 compared to BCP1 (Table 2). However, a similar number of genes predicted for nitrate and nitrite utilization and ammonia assimilation was annotated in BCP1 and R7 genomes (29 and 33 genes, respectively) (S5 Table). The higher activity values shown by BCP1 compared to R7 on ε-amino-N-caproic acid, D,L-α-amino-caprylic acid and δ-amino-N-valeric acid cannot be related to any genetic trait. However, some of the metabolic features of R7, not observed with BCP1, could be linked to the following genetic aspects. i) Considering the asparagine/aspartate metabolism, R7 has an asparaginase (EC 3.5.1.1) that converts asparagine in aspartate and an aspartate-ammonia lyase (EC 4.3.1.1) that transforms the aspartate in fumarate and ammonia that can be used as nitrogen source; the genes coding for the asparaginase and the aspartate-ammonia lyase are clustered together with a L-asparagine permease that is missing in BCP1 genome; both BCP1 and R7 have the asparagine synthetase (EC 6.3.5.4) that produces asparagine by ligating a NH_3_ group to aspartate using ATP; BCP1 genome lacks of an aspartic acid transporter. ii) Considering the arginine metabolism, R7 has genes coding for an arginase (EC 3.5.3.1) and an urease (EC 3.5.1.5) that convert arginine in urea and this in carbon dioxide and ammonia, respectively; R7 has also proton/glutamate aspartate symport protein that can mediate the import of glutamate and aspartate. iii) Considering the glutamine metabolism, the significantly higher activity of R7 on L-glutamine compared to BCP1 (at least double of BCP1 value) can be correlated with the presence of a L-glutaminase (EC 3.5.1.2) that hydrolyses L-glutamine in glutamate and ammonia. iv) considering the tyrosine metabolism, R7 has a tyramine:oxygen oxidoreductase (EC 1.4.-) which converts the tyramine that is included in tyrosine metabolism into 4-hydroxyphenylacetaldehyde and ammonia. This enzyme could also be related to the higher ability of R7 to utilize tyramine as carbon source.

**Fig 6:**
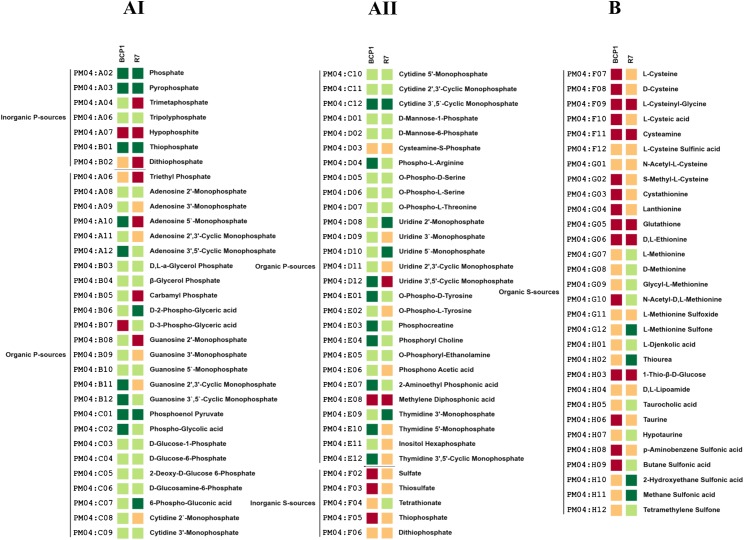
Phenotype Microarray PM with different Phosphorous and Sulphur Sources. Metabolic differences among *R*. *opacus* R7 and *Rhodococcus* sp. BCP1 in presence of different Phosphorous Sources (AI, AII) and Sulphur sources (B). Based on activity values of phenotype microarray analysis, threshold values were established for every plates. Determined thresholds were high (green), upper middle (light green), lower middle (orange) and low (red) for high, upper middle, lower middle and low activity, respectively.

R7 and BCP1 showed metabolic activity on most of the compounds tested in PM4 (80 and 90%, respectively) indicating a high versatility in phosphorous sources utilization (Fig 6 Panel AI, AII). A great difference was observed in sulphur sources assimilation capacity between BCP1 and R7. Indeed, BCP1 could utilize only 6 sulphur sources out of the 35 tested compounds in PM4 while R7 could assimilate 32 of these compounds (Fig 6 Panel B). This difference can be associated with the great difference between the two strains in terms of genes predicted to be involved in sulphur metabolism (S5 Table).

R7 showed more resistance to low pH compared to BCP1, particularly in presence of several amino acids. Both BCP1 and R7 showed wide resistance to osmolytes. The differences between the two strains regard: i) the higher resistance of R7 to increasing concentration of NaCl, sodium formate and sodium lactate; ii) the higher resistance of BCP1 to increasing concentration of urea (S2 and S3 Figs).

Using the PM11-PM20 plates, the two *Rhodococcus* strains were assayed for their sensitivity to 240 chemical agents (S4, S5 and S6 Figs) tested at four different concentrations. Both the *Rhodococcus* strains were resistant to the presence of several antibiotics (S4 Fig Panel AI, AII, AIII). In particular, they could grow in the presence of all the tested concentrations of the antibiotics belonging to quinolone, fluoroquinolone, cephalosporin, sulfonamide, aminocoumarin (novobiocin) classes. Amongst the tested β-lactam and glycopeptide antibiotics, only oxacillin and vancomycin, significantly inhibited the growth of both the two strains. Both R7 and BCP1 presented seven β-lactamase (EC 3.5.2.6) resistance genes in their genomes. Conversely, BCP1 and R7 showed general sensitivity to macrolide antibiotic class (i.e. erythromycin, josamycin and spiramycin) and to rifamycin group (i.e. rifampicin and rifamycin). For the tetracycline group, only minocycline was toxic for BCP1 and R7 strains. Only BCP1 genome presented one gene coding for a tetracycline resistance protein. Amongst the aminoglycoside antibiotic, both R7 and BCP1 were resistant to all the concentrations of kanamycin, spectinomycin, geneticin and dihydrostreptomycin. The two *Rhodococcus* spp. strains also showed high resistance to amikacin, gentamycin, sisomicin, tobramycin and hygromycin (S4 Fig Panel AI). R7 did not grow on any concentration of paromomycin while BCP1 was tolerant to the lowest concentration of this aminoglycoside antibiotic. Amongst the other tested chemicals (preservatives, drugs etc.) (S5 and S6 Figs Panel AI, AII and Panel AIII, AIV), R7 and BCP1 were in general resistant to the amino acid hydroxamates (protein synthesis inhibitors) and to the nucleic acid analogs. Amongst the quaternary sodium salts, R7 and BCP1 were resistant only to dequalinium. Other compounds such as ionophores (CCCP and FCCP) and the respiration inhibitor iodonitrotetrazolium chloride, inhibited the growth of the two strains even at the lowest concentration. Some of these phenotypic features can be attributed to the presence in their genomes of a high number of genes coding for multidrug resistance proteins and transport systems (20 in R7 and 16 in BCP1). Discrimination between BCP1 and R7 was shown in few cases. In particular, a higher tolerance was shown by R7 compared to BCP1 in the presence of the phenol derivative pentachlorophenol, the antifungal compound patulin and the β-lactam penicillin G. Conversely, BCP1 was more resistant than R7 to hexachlorophene (membrane electron transport inhibitor). Both BCP1 and R7 showed a general tolerance to metal salts even at the highest concentrations. Exceptions include: i) the low/medium tolerance shown by both strains to sodium *m*-periodate, antimony (III) chloride and sodium caprylate (growth observed at the lowest concentration); ii) the sensitivity to vanadate sodium salts (sodium metavanadate and sodium orthovanadate) that inhibited the growth of the two *Rhodococcus* spp. at all the tested concentrations. Thallium acetate was the only metal that had a different effect on the two strains as it inhibited R7 growth at all the concentrations while BCP1 showed tolerance to two out of the four concentrations of this metal salt (S6 Fig Panel B).

### Phenotype Microarray on organic/xenobiotic compounds by *Rhodococcus opacus* R7 and *Rhodococcus* sp. BCP1

**Fig 7:**
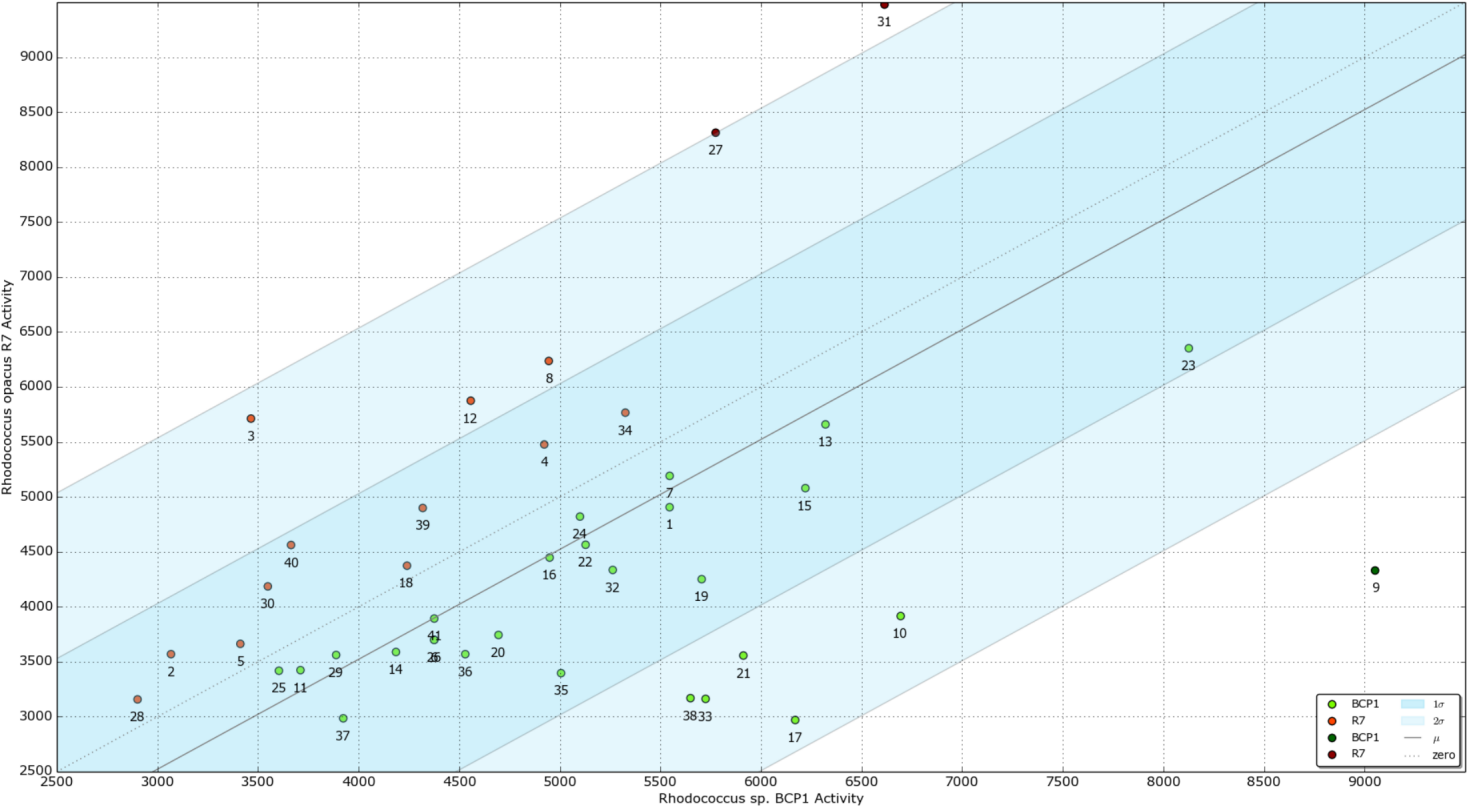
Phenotype Microarray PM with xenobiotic compounds (Scatter Plot). A three diagonal lines represented the mean (μ_A_), standard deviation (σ_A_) and double standard deviation (2σ_A_) of the difference activity (ΔA) of the two genomes overlapped. It highlighted regions of specificity for the genomes of the two strains on the tails of the gaussian distribution: *R*. *opacus* R7 specific compounds are in regions where ΔA <-σ_A_ (red points) and *Rhodococcus* sp. BCP1 specific compounds are on the other extreme points: ΔA > +σ_A_ (green points). Numbers in the plot represent the tested chemicals reported in the **Table 8**.

*R*. *opacus* R7 and *Rhodococcus* sp. BCP1 metabolic activity was tested on 41 organic/xenobiotic compounds supplied as sole carbon and energy source. The tested substrates belong to four chemical categories including: 1) aliphatic hydrocarbons and cycloalkanes, 2) BTEX and other aromatic compounds, 3) polycyclic aromatics (PAHs), 4) naphthenic acids and other carboxylic acids. Phenotype Microarray analysis was conducted following both the tetrazolium-based metabolic activity and the growth measured as OD_590_ increment over 72 h. Both strains were able to grow on several hydrocarbons and their putative metabolic intermediates (Fig 7) (S6 Table). Based on the growth results in presence of several linear- and cycloalkanes, *Rhodococcus* sp. BCP1 showed a general high activity on all *n*-alkanes tested ranging from *n*-hexane to *n*-hexatriacontane; while *R*. *opacus* R7 had a preferential activity on *n*-dodecane, *n*-tetradecane, *n*-hexadecane and *n*-eicosane. These data confirmed previous works on BCP1 and R7 strains reported by Cappelletti et al., 2011 and by Zampolli et al., 2014 [29, 25]. Moreover, results showed that R7 strain was not able to grow in presence of odd-alkanes, except for *n*-heptadecane. Both the two strains evidenced activity on cyclohexane and cyclohexanone; however these substrates were preferentially utilized by BCP1 strain. In addition, high level of activity was revealed for both R7 and BCP1 strains on fuel oil, a mixture of different alkanes, including prystane and phytane.

Growth analyses on aromatic hydrocarbons showed that both *Rhodococcus* strains had the same capacity to degrade common aromatic compounds as toluene and ethylbenzene. In accordance with results reported by Di Gennaro et al., 2010 [24], R7 has the ability to grow only on the *ortho* xylene isomer, while BCP1 showed the capability to utilize preferentially the *meta-* and *para-* isomers. In presence of dibenzothiophene (DBT) and of 2-hydroxybiphenyl, R7 showed the highest activity.

Another class of substrates tested on *Rhodococcus* spp. strains are polycyclic aromatic hydrocarbons (PAHs) and their metabolic intermediates. Generally PAHs are metabolized by two main reactions: the peripheral one transforming a large number of polycyclic aromatic derivatives into central intermediates; and the ring cleavage reaction of intermediates to products that are subjected to the TCA cycle. Phenotype Microarray results showed that R7 preferentially degraded PAHs compounds compared to BCP1, as for example in the case of anthracene. It was also investigated the ability of the two *Rhodococcus* strains to grow on putative PAH degradation intermediates. Results showed a good level of activity on gentisic acid, whereas a lower level was reported on salicylic acid. These experiments confirmed data from a previous work on R7 strain [24], and they suggest the identification of hypothetical intermediates of naphthalene pathway in BCP1 strain.

In presence of different naphthenic acids both strains reported a significant response with a higher activity for *Rhodococcus* sp. BCP1 on the cyclopentanecarboxylic acid (CPCA) and cyclohexanecarboxylic acid (CHCA). On the contrary, both strains responded in a similar manner to different acids that could be possible intermediates of different metabolic pathways, such as 4-phenylbutyric acid, hexanoic acids and 1,4-cyclohexane dicarboxylic acid.

The correlation between the phenotype showed by each strain and the hypothetical genotype was analysed using an adjustment of Ductape software. The enzyme functions involved in the metabolism of 19 substrates out of the 41 tested were predicted for BCP1 and for R7 strains by the software (Table 8). The majority of unidentified enzymes were related to the metabolism of *n*-alkanes and naphthenic acids. Indeed, *n*-alkanes and naphthenic acids were not associated to any known enzymes because of lacking in the KEGG’s annotation system. Conversely, the metabolism of cycloalkanes and their possible metabolic intermediates was associated to the activity of monooxygenases and deyhdrogenases. For instance, the ability to utilize cyclohexanone was correlated to the activity of a cyclohexanone dehydrogenase (EC 1.3.99.14) and a cyclohexanone monooxygenase (EC 1.14.13.22).

**Table 8:** List of the xenobiotic compounds tested in phenotype microarray analysis and EC the numbers of the enzymes identified in R7 and BCP1 genomes and predicted to be involved in their metabolisms by DuctApe software.

Index	Chemical	EC numbers for *R*. *opacus* R7 and *Rhodococcus* sp. BCP1
1	1,4-Cyclohexane dicarboxylic acid	unknown
2	1-Adamantanecarboxylic acid	unknown
3	2-Hydroxybiphenyl	1.14.13.44; 3.13.1.3
4	4-Phenylbutyric acid	unknown
5	5,6,7,8-Tetrahydro-2-naphthoic acid	unknown
6	Benzene	1.14.12.3; 1.14.13.-
7	Cyclohexane	1.14.15.-
8	Cyclohexanecarboxylic acid	6.2.1.-
9	Cyclohexanone	1.1.1.90; 1.1.1.245; 1.3.99.14; 1.4.3.12; 1.14.13.22; 4.1.3.35
10	Cyclopentanecarboxylic acid	unknown
11	Decane	unknown
12	Dodecane	unknown
13	Eicosane	unknown
14	Ethylbenzene	1.14.12.12; 1.14.12.-; 1.17.99.2; 1.17.-.-
15	FuelOil	unknown
16	Heptadecane	4.1.99.5
17	Heptane	unknown
18	Hexadecane	unknown
19	Hexatriacontane	unknown
20	Naphthalene	1.14.12.12; 1.14.13.-; 1.14.14.1
21	Nonane	unknown
22	Octacosane	unknown
23	Tetracosane	unknown
24	Tetradecane	unknown
25	Toluene	1.14.12.11; 1.14.13.-; 1.14.15.-; 4.1.99.11
26	Tridecane	unknown
27	Anthracene	1.14.12.12; 1.14.-.-
28	Cyclohexane butyric acid	unknown
29	Cyclohexaneacetic acid	unknown
30	Decanoic acid	3.1.2.21
31	Dibenzothiophene	unknown
32	Gentisic acid	1.2.1.29; 1.2.3.1; 1.13.11.4; 1.14.13.24; 1.14.13.172; 4.1.1.62
33	Hexane	unknown
34	Hexanoic acid	3.5.1.39
35	*m*-Xylene	1.14.13.-; 1.14.15.-
36	Methyl-cyclohexanecarboxylic acid	6.2.1.-
37	*o*-Xylene	1.14.13.-; 1.14.15.-
38	*p*-Xylene	1.14.13.-; 1.14.15.-
39	Phenanthrene	1.13.11.-; 1.14.13.-
40	Salicylic acid	1.2.1.65; 1.2.1.-; 1.14.13.1; 1.14.13.172; 1.14.13.-; 2.1.1.274; 3.1.1.55; 3.7.1.8; 4.1.1.91; 4.1.1.-; 4.2.99.21
41	Trans-1,2-cyclohexane dicarboxylic acid	unknown

The enzyme families predicted to be implicated in BTEX metabolism by R7 and BCP1 strains were: monooxygenases (EC 1.14.13- and 1.14.15-) involved in different hydrocarbon degradations; benzene/toluene dioxygenases (EC 1.14.12.3 and EC 1.14.12.11) and several putative enzymes involved in ethylbenzene metabolism, like the naphthalene dioxygenase (EC 1.14.12.12), the ethylbenzene hydroxylase (EC 1.17.99.2) and a putative dehydrogenase (EC 1.17-).

Concerning the activity on PAHs, amongst the 129 oxygenases/hydroxylases annotated for *R*. *opacus* R7 by RAST, 61 enzymes were predicted to be involved in mono- and polycyclic aromatic hydrocarbon metabolism. In BCP1, 73 oxygenases/hydroxylases amongst the 134 annotated genes, are predicted to be related to the oxidation of these organic compounds. Within PAHs, the analysis by Ductape of Phenotype Microarray showed anthracene and naphthalene to be metabolized by the same enzyme, a naphthalene dioxygenase (EC 1.14.12.12). Conversely the metabolism of phenanthrene was correlated to a PAH dioxygenase (EC 1.13.11.-). The use of gentisic acid was correlated to the gentisate 1,2-dioxygenase (EC 1.13.11.4); while the use of salicylic acid was related to salicylate 5-hydroxylase (EC 1.14.13.172). Amongst the naphthenic acids, the only substrate associated to an enzymatic class was the cyclohexanecarboxylic acid (CHCA), related to the large group of CoA synthetases (EC 6.2.1.1). Regarding this enzyme class, acetate and longer chain carboxylic acids can be converted to-CoA derivatives by CoA synthetases (EC 6.2.1.1) [16].

### Genetic aspects related to the great potential for organic/xenobiotic degradation in *Rhodococcus opacus* R7 and *Rhodococcus* sp. BCP1

Based on the genome sequences of *R*. *opacus* R7 and *Rhodococcus* sp. BCP1 and their annotations, functional gene clusters involved in the degradation of different organic/xenobiotic compounds were investigated. Four chemical categories of compounds were considered including: 1) aliphatic hydrocarbons and cycloalkanes; 2) BTEX and aromatic compounds; 3) polycyclic aromatic compounds (PAHs); 4) naphthenic acids and other carboxylic acids. The gene-associated functions of the catabolic clusters were predicted on the basis of the NCBI genomic database, the Rapid Annotation using Subsystem Technology (RAST server), the Kyoto Encyclopedia of Genes and Genomes (KEGG) pathway database and manual curation.

#### Aliphatic hydrocarbons and cycloalkanes degradation

**Fig 8:**
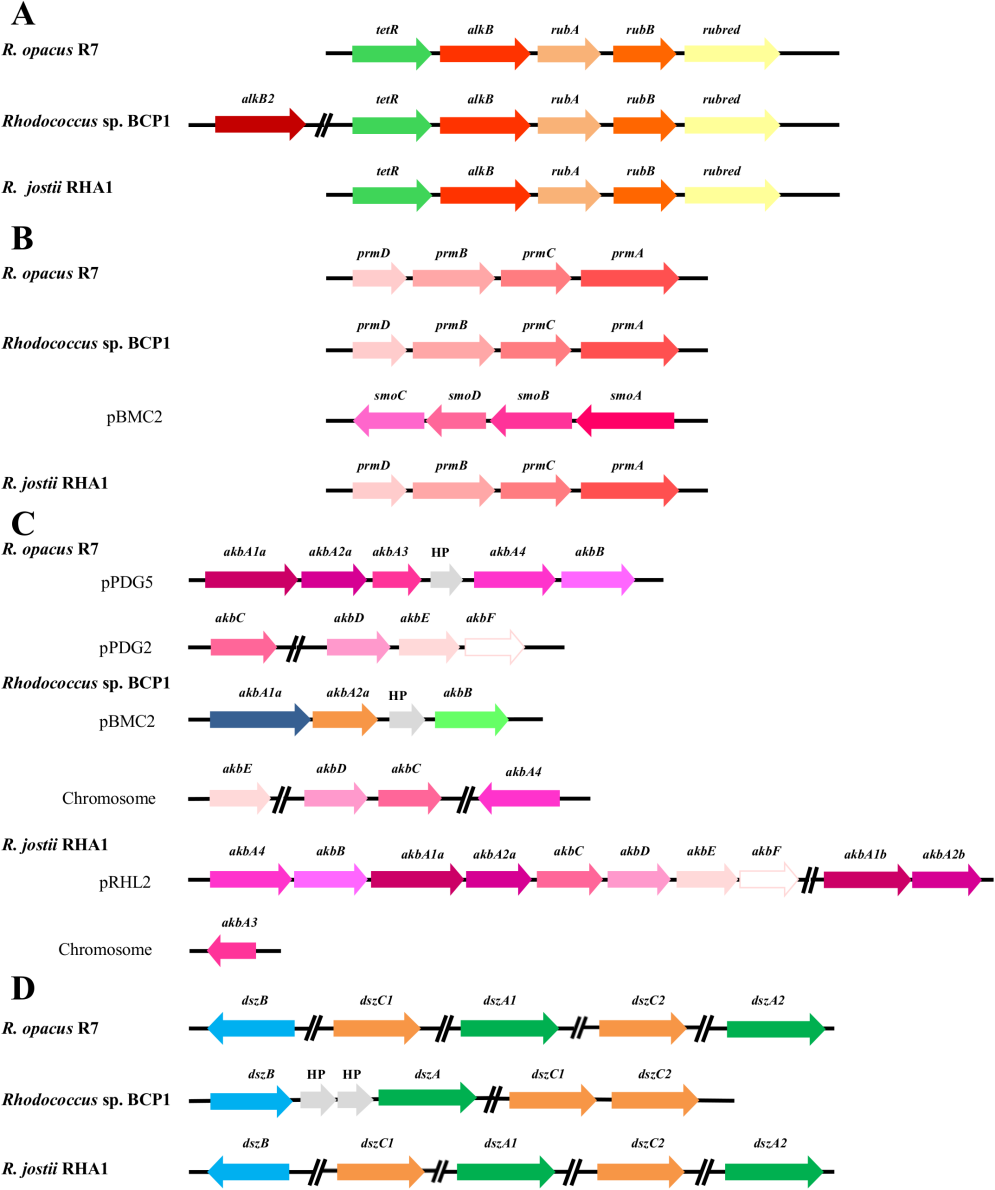
Comparison of *gene clusters* from R7 and BCP1 genomes correlated to aliphatic and aromatic hydrocarbon degradations. Comparative organization of genetic determinants for the tested aliphatic and aromatic hydrocarbons in *R*. *opacus* R7 and *Rhodococcus* sp. BCP1 with *R*. *jostii* RHA1 as reference strain. Predicted genes (listed in **S7, S8, S9, S10 Tables**) and their orientation are shown by arrow. (A) *alk* gene cluster; (B) *prm* and *smo* gene cluster (*smo* gene cluster was organized in *smoA* encoding a methane monooxygenase component A, *smoB* encoding a methane monooxygenase component B, *smoD* and *smoC* encoding a methane monooxygenase regulatory protein and a methane monooxygenase component C, respectively); (C) *akb* gene cluster; (D) *dsz* gene cluster. When not specified, it means that genes were located on chromosome. Genes with unknown or hypothetical functions were reported as HP. Double slash indicates a distances between two genes more than 1 kb within the same plasmid or chromosome.

The presence of catabolic genes involved in the growth on short-, medium- and long-chain *n*-alkanes was previously investigated in both *R*. *opacus* R7 and *Rhodococcus* sp. BCP1 strains [25, 28, 29]. Compared with these studies, the presence of only one copy of the *alkB* gene was confirmed in *R*. *opacus* R7, while in *Rhodococcus* sp. BCP1, two copies of this gene (*alkB1* and *alkB2)* were found within the chromosome (Fig 8 Panel A). One *alkB* gene in each strain was organized in a cluster associated in an operon with four consecutive coding sequences (CDSs): *alkB* coding for an alkane monooxygenase, *rubA* and *rubB* coding for two rubredoxins, and *rubred* coding for a rubredoxin reductase, and, in addition, the *tetR* gene coding for a regulator. In BCP1 genome, an additional 1161-bp *alkB* gene (*alkB2*) was identified. This gene was not organized in operon with *rubA/B* and *rubred* genes but was associated with genes coding for a putative esterase and a long-chain-fatty-acid-CoA ligase (EC 6.2.1.3), involved in the fatty acid β-oxidation. The *alkB2* gene product showed a limited similarity (57% amino acid identity) with *alkB1* gene product of BCP1. The gene products of *alkB1* cluster of BCP1 showed high similarity (S7 Table) with those coded by *alk*B cluster of R7 and with the ones of the most known bacteria belonging to the *Rhodococcus* genus such as *R*. *jostii* RAH1 or *R*. *opacus* PD630 [14, 16].

Two *prm* gene clusters were identified and analyzed in BCP1 (Fig 8 Panel B). The first *prm* gene cluster, constituted by the *prmA*,*C*,*B*,*D* genes, was found in the chromosome and it has been proven to be specially involved in the degradation of propane and butane; the second cluster, also named *smo* gene cluster (soluble di-iron monooxygenase) (*smoA*,*B*,*D*,*C* genes), was found in the pBMC2 plasmid and it was described to be involved in the growth of BCP1 on a wide range of short-chain *n*-alkanes [28]. The comparison of the BCP1 *prm* gene cluster with R7 genome sequences allowed the identification of a *prm*-like gene cluster in R7 chromosome with a percentage of amino acid identities with the corresponding genic products of 90% (S8 Table). Despite of the high sequence similarity between the two *prm* clusters, no growth of R7 in presence of gaseous *n*-alkanes was observed [25].

R7 and BCP1 strains showed also a considerable number of genes coding for P450 monooxygenases, 23 and 18 predicted coding sequences, respectively. We can not exclude that these putative enzymes are also involved in the aliphatic hydrocarbon degradation.

#### Aromatic hydrocarbons degradation

Both R7 and BCP1 strains are able to grow on different aromatic hydrocarbons, including BTEX compounds. The majority of the *Rhodococcus* genomes sequenced, as in the case of *R*. *jostii* RHA1, were characterized for the ability to grow on BTEX and in some cases also for their genetic organization [14, 44]. In *Rhodococcus jostii* RHA1, the *akb* gene cluster was identified as the cluster involved in the metabolism of alkyl-benzenes. It contains two genes (designated *akbA1a* and *akbA1b*, approximately 6 kb apart from each other) encoding an oxygenase component large subunit, each one followed by genes (designated *akbA2a* and *akbA2b*) encoding a small subunit oxygenase component. Moreover, the *akb* cluster consists of a reductase (AkbA4), a ferredoxin (AkbA3) component, an *akbB gene* and *akbCDEF* genes coding for the lower pathway enzymes of the ring cleavage. Comparing the *akb* gene cluster of RHA1 with R7, we found the same gene cluster divided in two parts and each part was allocated on two plasmids (Fig 8 Panel C): *akbA1a*, *akbA2a*, *akbA3*, *akbA4*, *akbB* genes were found on the pPDG5 plasmid; while *akbCDEF* genes, putatively coding for a complete *meta*-cleavage pathway, constituted by a *meta*-cleavage dioxygenase (AkbC), a *meta*-cleavage hydrolase product (AkbD), a hydratase (AkbE), and an aldolase (AkbF), were identified on the pPDG2 plasmid with high amino acid identity (S9 Table). An homologous sequence of *akb* cluster was also found on the chromosome and on the pPDG4 plasmid, but they showed a protein identity around 35%.

RAST analysis of BCP1 genome showed that coding sequences homologous to the *akb* genes were found in pBMC2 plasmid: *akbA1a* was found similar to a gene annotate as a large subunit *nah*/*bph* dioxygenase with an amino acid identity of 36%, *akbA2a* similar to a gene annotated as a biphenyl dioxygenase beta subunit (EC 1.14.12.18) with an amino acid identity of 36% and *akbB* similar to a gene annotated as a 2,3-dihydroxy-2,3-dihydro-phenylpropionate dehydrogenase (EC 1.3.1.-) with 47% protein identity.

These data indicated a redundancy of genes involved in the metabolism of the aromatic compounds in R7 strain, probably due to its isolation from a contaminated polycyclic aromatic hydrocarbons soil. The presence of multiple copies in several plasmids can derive from transposition events and duplication of the same genes. These data are confirmed by the presence on the pPDG5 plasmid of five mobile elements and transposases upstream the *akb* genes. In BCP1 only one copy of a putative cluster involved in the degradation of aromatic hydrocarbons was found.

In presence of dibenzothiophene (DBT) R7 strain showed also the highest activity of growth and the presence of putative genes involved in this metabolism was investigated. Some other *Rhodococcus* strains, including RHA1, are able to utilize dibenzothiophene as a sole source of sulphur due to the expression of *dsz* operon, which encodes three proteins, DszA, B and C. DszC catalyses the stepwise *S*-oxidation of DBT, first to dibenzothiophene 5-oxide (DBTO) and then to dibenzothiophene 5,5-dioxide (DBTO_2_); DszA catalyses the conversion of DBTO to 2-(2’-hydroxyphenyl) benzene sulphinate (HBPSi^-^) and DszB catalyses the desulphation of HBPSi^-^ to give HBP and sulphonate [45]. A *dsz* gene cluster was found in R7 and BCP1 genomes (Fig 8 Panel D). Comparing the protein sequence of DszA, two different oxygenases (*dszA1 and dszA2*) were identified in R7 chromosome with amino acid identity of 98% and 97% (S10 Table). Sequence analysis revealed also two *dszC* genes (*dszC1 and dszC2*) predicted to code for dibenzothiophene desulphurization enzymes, and not so far we found only one copy of the *dszB* gene encoding an ABC sulfonate transporter protein.

In BCP1 chromosome four coding sequences were identified as homologous to the *dsz* sequences of R7 genome. As in the R7 strain, two *dszC* genes (*dszC1 and dszC2*) were found located close to each other with a percentage reported in S10 Table; while only one *dszA* gene and one *dszB* gene copy were found clustered together on the chromosome. These preliminary indications suggest that R7 and BCP1 strains could have genes involved in the DBT degradation.

#### Polycyclic aromatic hydrocarbons degradation

**Fig 9:**
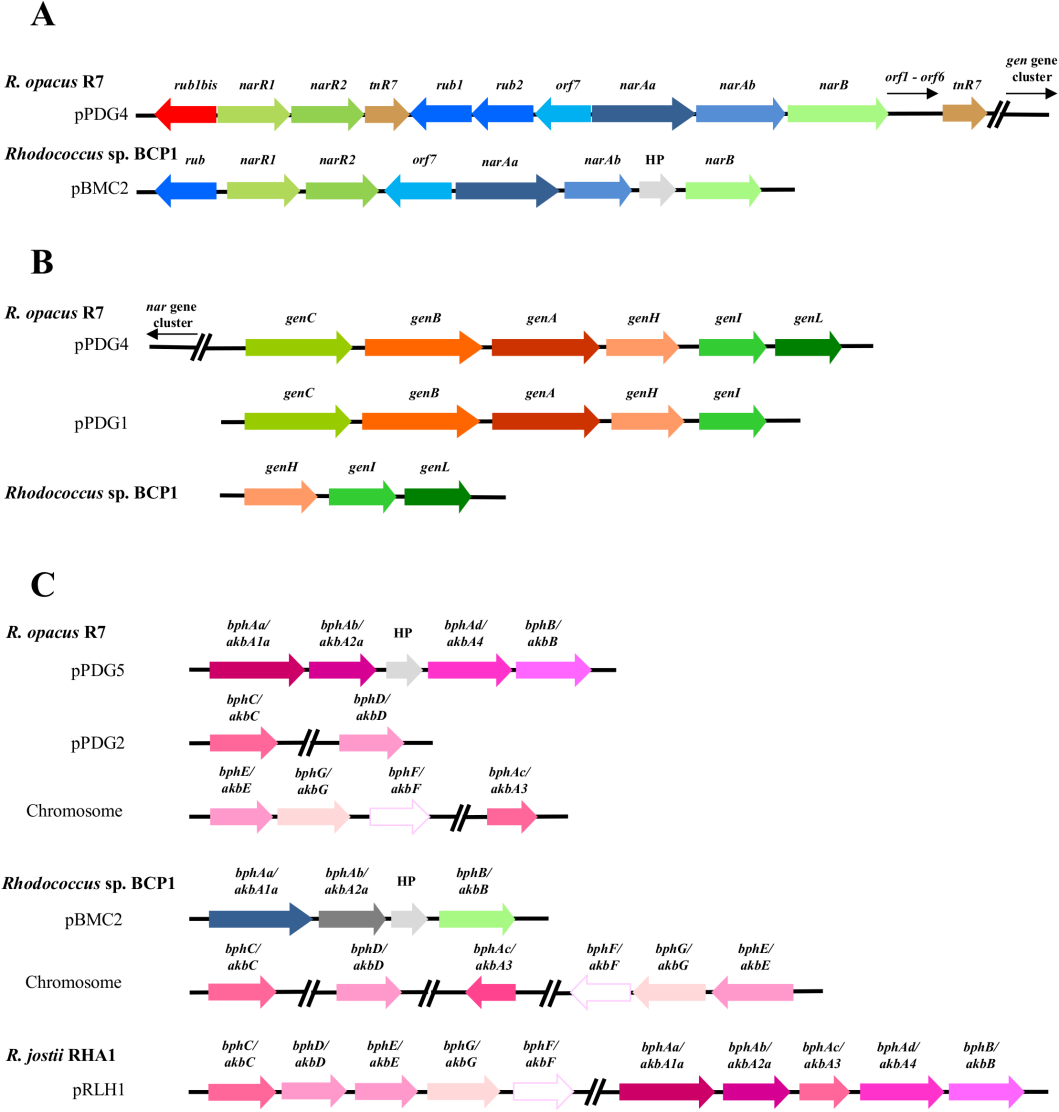
Comparison of *gene clusters* from R7 and BCP1 genomes correlated to polycyclic aromatic hydrocarbon degradations. Comparative organization of genetic determinants for naphthalene and biphenyl (as reference compounds of PAH and putative intermediates) in *R*. *opacus* R7 and *Rhodococcus* sp. BCP1 with *R*. *jostii* RHA1 as reference strain. Predicted genes (listed in **S11, S12 and S13 Tables**) and their orientation are shown by arrow. (A) *nar* gene cluster; (B) *gen* gene cluster; (C) *bph* gene cluster organization. When not specified, it means that genes were located on chromosome. Genes with unknown or hypothetical functions were reported as HP. Double slash indicates a distances between two genes more than 1 kb within the same plasmid or chromosome.

In a previous work, genes involved in naphthalene (*nar* gene cluster) and salicylate (*gen* gene cluster) degradation were found in *R*. *opacus* R7 [24]. The whole genome sequence analysis pointed out that *nar* gene cluster is located in pPDG4 plasmid in R7 strain and that the same cluster was found in pBMC2 in *Rhodococcus* sp. BCP1 (Fig 9 Panel A); this genomic region identified in pBMC2 is the same identified as *akb* gene cluster in BCP1. In S11 Table, protein identities are reported. The whole genome sequence analysis revealed the presence of *orf7* within the cluster, as in R7 strain, while any of the other six CDSs identified in R7 were found in BCP1 strain. An homologous *nar* gene cluster was not found in the reference strain *R*. *jostii* RHA1. The lower naphthalene catabolic pathway was previously investigated in R7 and it was hypothesized that the *gen* gene cluster was located far from the *nar* region as no amplification of the middle region was obtained. Genome sequence analysis confirmed these data (Fig 9 Panel B). Moreover, two copies of the gene clusters involved in naphthalene lower pathway were found in R7: one in the pPDG4 plasmid distant 12.4 kb from *nar* gene cluster; the other in the pPDG1 plasmid, but lacking of *genL* gene. Comparison of this cluster with genome sequence of BCP1 revealed that some genes involved in gentisate oxidation were found: *genH* and *genI* genes were found in the chromosome with high protein identity (around 80%), followed by *genL* gene that showed a protein identity of 48% with the homologous genes of R7. Instead, genes involved in salicylate degradation, *genA*, *B*, *C* were found in different regions of the BCP1 chromosome showing a lower amino acid identity as reported in S12 Table. This could be the reason of the low activity level shown by BCP1 during the growth on salicylic acid.

A well-known microorganism characterized for its ability to degrade biphenyls is *Rhodococcus jostii* RHA1. Genes involved in this pathway were identified in two different plasmids: pRHL1 and pRHL2 [46, 47]. In the biphenyl metabolic pathway, biphenyl is transformed to 2,3-dihydroxy-1-phenylcyclohexa-4,6-diene (dihydrodiol) by a multicomponent biphenyl dioxygenase (BphA). Dihydrodiol is converted to 2,3-dihydroxybiphenyl (2,3DHBP) by dihydrodiol dehydrogenase (BphB). 2,3DHBP is cleaved at the 1,2 position (*meta*-ring cleavage) by 2,3DHBP dioxygenase (BphC). The ring cleavage product (2-hydroxy-6-oxo-6-phenylhexa-2,4-dienoate [HPDA]) is hydrolyzed to benzoate and 2-hydroxypenta-2,4-dienoate by HPDA hydrolase (BphD), and the resulting 2-hydroxypenta-2,4-dienoate is further converted to tricarboxylic acid cycle intermediates by 2-hydroxypenta-2,4-dienoate hydratase, 4-hydroxy-2-oxovalerate aldolase, and acetaldehyde dehydrogenase (BphE, BphF, and BphG, respectively). Thus, the products of a set of catabolic genes, *bphAa*,*Ab*,*Ac*,*Ad*,*B*,*C*,*D*,*E*,*F*,*G* are responsible for the aerobic metabolism of biphenyl. The *bph* gene cluster of RHA1 was compared with the genome sequences of R7 and BCP1 (Fig 9 Panel C). We found different genes of this cluster in R7 chromosome and in pPDG2 and pPDG5 plasmids. In particular, R7 showed genes encoding biphenyl large and small subunits and dihydrobiphenyldiol dehydrogenase, involved in the first two steps of dioxygenation of biphenyl, on the pPDG5 plasmid; whereas genes encoding the ring-cleavage were found on the pPDG2 plasmid. Some homologous sequences of *bph* cluster were also found in R7 chromosome (S13 Table).

RAST analysis of BCP1 genome identified CDSs homologous to *bph* genes in pBMC2. In particular, only large and small subunits of a dioxygenase (*bphAa* and *bphAb* genes) and a dihydrobiphenyldiol dehydrogenase (*bphB* gene) were found with an amino acid identity around 40%; this coincides with the region identified as *akb* gene cluster and *nar* gene cluster. Also in BCP1, the genes encoding the ring-cleavage enzymes were not found near the cluster within the plasmid pBMC2; however, an homologous coding sequence was found in the chromosome with low protein identity (around 30%) (S13 Table). Results allowed to hypothesize that the same BCP1 cluster was involved in the first oxidation steps of several aromatic and polycyclic aromatic hydrocarbons, such as BTEX compounds, naphthalene and biphenyls. Indeed, we can attribute the same sequence to the *akb*, *nar* and *bph* gene clusters.

#### Naphthenic acids degradation

**Fig 10:**
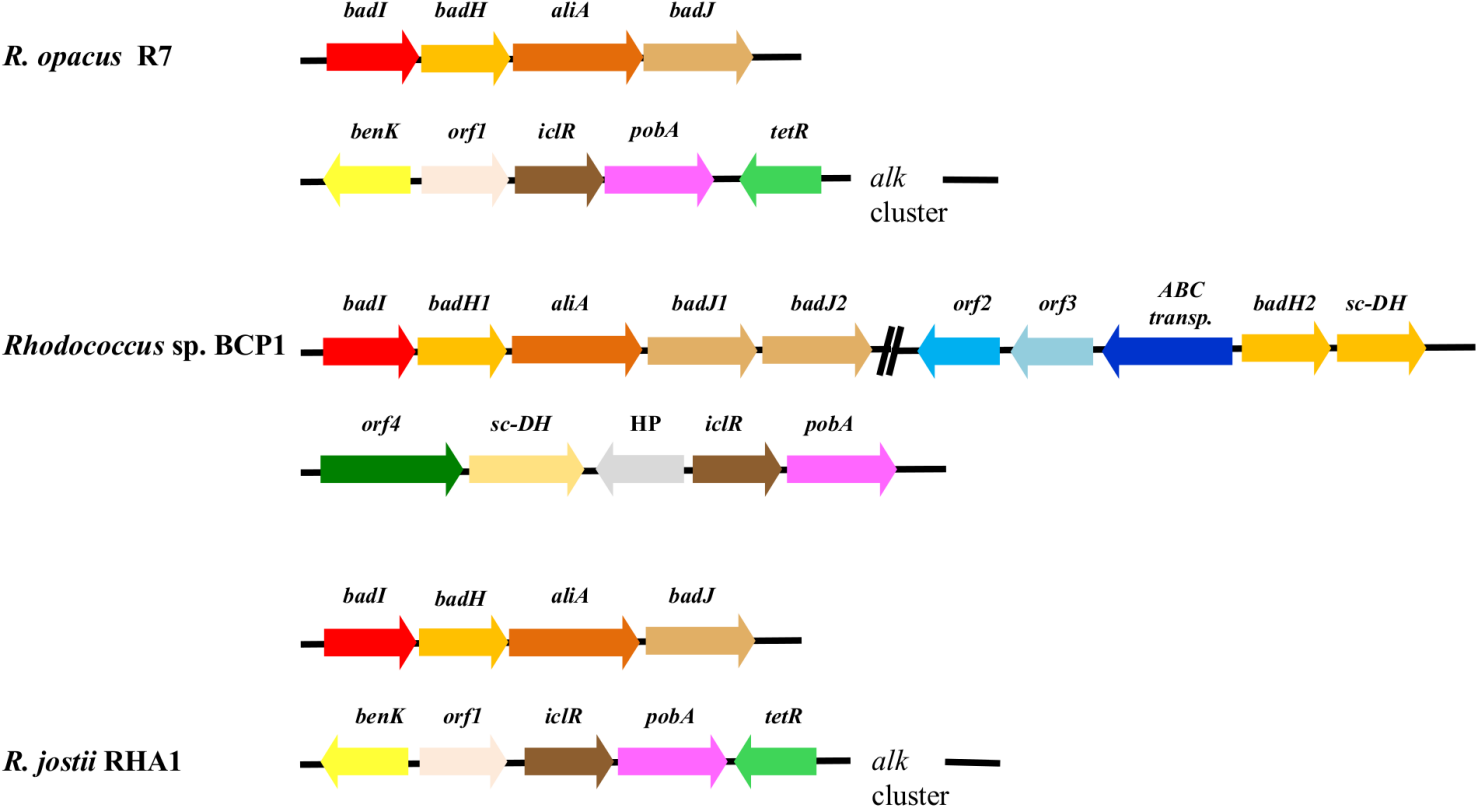
Comparison of *gene clusters* from R7 and BCP1 genomes correlated to carboxylated hydrocarbon degradations. Comparative organization of genetic determinants for naphthenic acids (as reference compounds of carboxylated hydrocarbons and putative intermediates) in *R*. *opacus* R7 and *Rhodococcus* sp. BCP1 with *R*. *jostii* RHA1 as reference strain. Predicted genes (listed in **S14 Table**) and their orientation are shown by arrow. The following genes encode for: *benK*, benzoate transporter; *orf1*, O-antigen acetylase; *iclR*, transcriptional regulator IclR family; *tetR*, transcriptional regulator, TetR family; *orf 2* and *orf 3*, permease; *ABC transp*, ABC transporter; *sc-DH*, probable short-chain dehydrogenase; *orf4*, permease. When not specified, it means that genes were located on chromosome. Genes with unknown or hypothetical functions were reported as HP. Double slash indicates a distances between two genes more than 1 kb within the same plasmid or chromosome.

Considering the ability of the two strains to grow on these contaminants, the putative gene clusters involved in this degradation were investigated. In literature, few metabolic studies are available on the biodegradation of naphthenic acids (NAs) based on cyclohexane ring i.e. cyclohexane carboxylic acid (CHCA); while none has been focused on cyclopentane ring i.e. cyclopentane carboxylic acid (CPCA). Although no genetic information was provided in these studies [48, 49], the metabolism of CHCA was described to follow two main routes: (i) aromatization of the cycloalkane ring to produce hydroxybenzoate before ring opening [48], or (ii) activation of cycloalkane ring as CoA thioester-derivative that is further degraded through β-oxidation steps [49]. Iwaki and co-workers identified the *pobA* gene to have an essential role for the growth on CHCA by *Corynebacterium cyclohexanicum*. This gene codes for 4-hydroxybenzoate (4-HBA) 3-hydroxylase generally described to be responsible for the conversion of 4-hydroxybenzoate to protocatechuate, a common intermediate in the degradation of various aromatic compounds. Iwaki and co-workers [48] demonstrated the involvement of *pobA* product in CHCA catabolism by *Corynebacterium* strain downstream of the formation of 4-hydroxybenzoate from CHCA through several oxidation steps. One gene homologous to *pob*A was identified in both BCP1 and R7 genomes and its product was annotated as *p*-hydroxybenzoate hydroxylase in RAST server. Similarly to what found in *Corynebacterium* strain, *pob*A gene was flanked by a gene coding for an IclR-type transcriptional regulator. The genomic region including *pob*A in R7 included also the *alkB* gene cluster, TetR-like regulator and a BenK transporter and the same organization was maintained between RHA1 and R7. On the contrary, the genomic organization of BCP1 region with *pobA* was different from those of R7 and RHA1 and included a permease coding gene and several oxidoreductases (Fig 10).

The activation of the cycloalkane ring as Co-A thioester-derivative was firstly described during the anaerobic degradation of benzoic acid by *Rhodopseudomonas palustris* [50]. CHCA is produced as metabolic intermediate during this pathway and it has been reported to be metabolized through β-oxidation. The enzymes responsible for CHCA degradation in *R*. *palustris* are encoded by the *bad* genes [51]. These enzymes degrade CHCA by catalyzing the following reactions: (i) activation of cyclohexanecarboxylate as cyclohexanecarboxylate-CoA by a CoA-ligase (AliA); (ii) cyclohexanecarboxylate-CoA is dehydrogenated to the corresponding aldehyde cyclohex-1-ene-1-carboxylate by the dehydrogenase AliB; (iii) the hydratase BadK converts the aldehyde in the secondary alcohol 2-hydroxycyclohexane-1-carboxyl-CoA; (iv) the dehydrogenase BadH is responsible for the formation of 2-ketocyclohexane-1-carboxyl-CoA from the secondary alcohol; (v) the hydratase BadI catalyses the the cyclohexane ring opening with the formation of pimelyl-CoA. On the basis of the amino acid identity percentage as compared with the 2-ketocyclohexane-carboxyl-CoA dehydrogenase (BadH) of *R*. *palustris*, two genes *(badH1 and badH2)* were found in BCP1 genome encoding two enzymes annotated as 2-hydroxycyclohexanecarboxyl-CoA dehydrogenase. Only one gene homologous to *badH* was found in R7 and, compared to BCP1, it possesses conserved flanking regions including: a long-chain-fatty-acid-CoA-ligase, two dehydrogenases and a naphthoate synthase. This region is also maintained in RHA1 (Fig 10) (S14 Table).

### Genetic aspects related to aromatic peripheral pathways in *Rhodococcus opacus* R7 and *Rhodococcus* sp. BCP1

**Fig 11:**
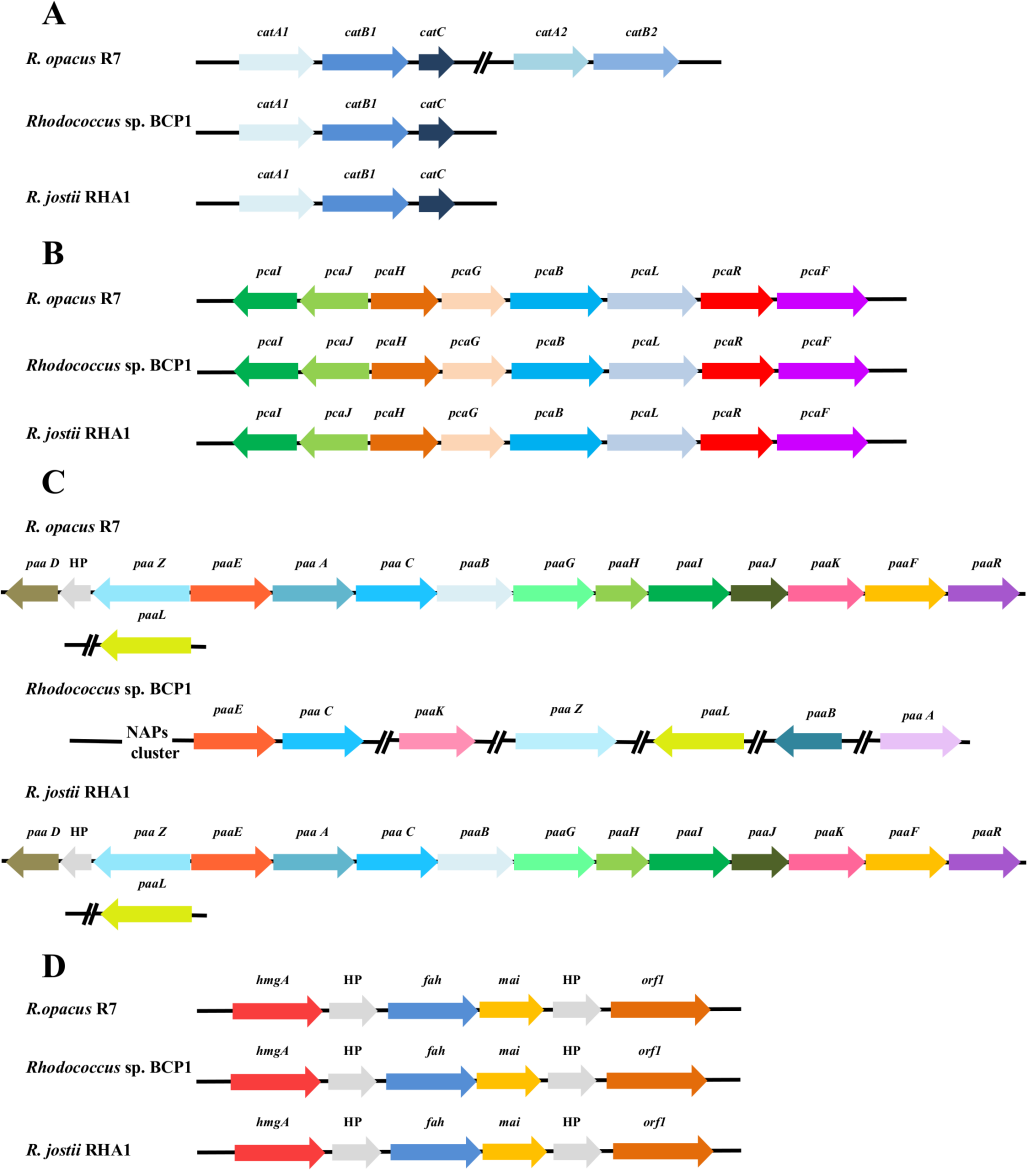
Comparison of *gene clusters* from R7 and BCP1 genomes correlated to xenobiotic peripheral pathways. Comparative organization of genetic determinants for xenobiotic peripheral pathways in *R*. *opacus* R7 and *Rhodococcus* sp. BCP1 with *R*. *jostii* RHA1 as reference strain. Predicted genes (listed in **S15, S16, S17 and S18 Tables**) and their orientation are shown by arrow. (A) *cat* gene cluster; (B) *pca* gene cluster; (C) *paa* gene cluster; (D) *hmg* gene cluster. When not specified, it means that genes were located on chromosome. Genes with unknown or hypothetical functions were reported as HP. Double slash indicates a distances between two genes more than 1 kb within the same plasmid or chromosome.

Considering the aromatic compounds that R7 and BCP1 can metabolize, four different peripheral pathways for the catabolism of several xenobiotics can be predicted, which include catechol (*cat* genes), protocatechuate (*pca* genes), phenylacetate (*paa* genes) and homogentisate (*hmg* genes) pathways. Genes responsable for such catabolic pathways have been reported in several bacteria [52], in particular in *R*. *jostii* RHA1. We performed a sequence comparison analysis to identify the genes predicted to be involved in these pathways in *R*. *opacus* R7 and in *R*. sp. BCP1. R7 genome contains several genes potentially involved in catechol catabolism. It shows six catechol 1,2-dioxygenases (five on the chromosome and one on pPDG2 plasmid), and three catechol 2,3-dioxygenases (one on the chromosome, one on pPDG2 and one on pPDG5 plasmid). BCP1 genome presents only two catechol 1,2-dioxygenases and one catechol 2,3-dioxygenase on the chromosome. These aspects might be explained on the basis of the significant difference of their genome size. Two catechol dioxygenase genes, amongst those identified on R7 chromosome, were organized in cluster (Fig 11 Panel A). The first *cat* gene cluster presented *catA (catA1)*, coding for a catechol 1,2-dioxygenase, *catB (catB1)* coding for a muconate cycloisomerase and *catC* coding for a muconolactone isomerase. The same gene cluster was identified in RHA1 and, compared to R7, it showed high protein identity (96–99%). The second *cat* gene cluster identified in R7 lacked of *catC* gene; moreover, CatA2 (*catA2*) and CatB2 (*catB2*) were not found homologous to RHA1 genes (S15 Table). *Rhodococcus* sp. BCP1 presented only one copy of *cat* gene cluster with the same organization of RHA1 and R7; BCP1 *cat* genes showed high similarity (70–90%) with those of RHA1 and R7 strains as reported in table (S15 Table).

Both R7 and BCP1 genomes contain several genes potentially involved in protocatechuate catabolism (Fig 11 Panel B). The putative R7 and BCP1 *pca* clusters include genes predicted to encode the enzymes (PcaIJHGBLF and the regulator) required to convert protocatechuate to the TCA cycle intermediates. The predicted products of these R7 genes share high amino acid similarity (97–99%) with their homologous from RHA1. The predicted products of BCP1 *pca* genes share 57%-82% of similarity with the homologous genes from RHA1 (S16 Table). The organization of the *pca* genes of both strains is similar to their organization in RHA1. Indeed, they are organized in two putative divergently transcribed operons, *pcaJI* and *pcaHGBLRF*.

Phenylacetate pathway for aerobic degradation of phenylacetic acid (PAA) can proceeds through the formation of phenylacetyl-coenzyme A (Co-A) and the subsequent hydrolytic ring fission. These metabolic steps in *R*. *jostii* RHA1 are catalized by enzymes coded by the *paa* gene cluster [53]. The organization of the *paa* gene cluster differs amongst different bacteria, but some features are conserved. Genes encoding two core functional units of the pathway that are consistently clustered, include *paaGHIJK*, which encodes a ring-hydroxylating system, and *paaABCE*, which encodes a β-oxidation system. The *paa* gene cluster organization (composed by 15 genes) was conserved in *R*. *jostii* RHA1 and *R*. *opacus* R7 genomes (Fig 11 Panel C). They also showed high percentage of similarity as reported in S17 Table. On the contrary, the *paa* gene cluster was not conserved in *Rhodococcus* sp. BCP1 and few genes homologous to those of RHA1, are present without a co-localization. R7 and BCP1 genomes analysis reported one homogentisate 1,2-dioxygenase (*hmgA*) in both strains (97% and 85% identity with RHA1 *hmgA*, respectively). The genetic organization of the *hmgA* flanking regions is quite similar between R7, BCP1 and RHA1, showing genes encoding an enoyl-CoA hydratase (EC 4.2.1.17), fumarylacetoacetase (EC 3.7.1.2) and a glutaryl-CoA dehydrogenase (EC 1.3.99.7). Similarly to RHA1, R7 showed upstream and downstream of these genes, two CDSs coding for long-chain-fatty-acid-CoA ligases (EC 6.2.1.3). In the same region, BCP1 genome reported only one gene coding for the same enzyme (Fig 11 Panel D) (S18 Table).

## Discussion

This work describes in detail the genomic, phenotypic and taxonomic features of two *Rhodococcus* spp. strains whose genomes have recently been sequenced [22, 23]. In particular, phylogenetic comparison of the two strains was performed using a multi-locus sequence analysis approach with four different taxonomic markers (*16S rRNA* gene, *secY* gene, *rpoC* gene and *rpsA* gene). *Rhodococcus* sp. BCP1 was phylogenetically related to *R*. *aetherivorans* species, while *R*. *opacus* R7 was related to both *R*. *opacus* and *R*. *wratislaviensis* species. The taxonomic correlation of BCP1 strain is in line with previous phylogenetic analysis performed using other taxonomic markers (i.e. alkane 1-monooxygenase (AlkB) [42]. These studies taken together support the belonging of BCP1 to *R*. *aetherivorans* species making its genome to be the first sequenced genome available of this species.

Comparative analyses have been performed amongst BCP1, R7 strains and other *Rhodococcus* strains taxonomically related. In particular, *R*. *opacus* PD630 and *R*. *opacus* B4 were considered because closely related to R7; *R*. *pirydinivorans* SB3094 was the most closely BCP1 related strain with a complete genome sequenced; *R*. *jostii* RHA1 represents the reference strain for genomic analysis of *Rhodococcus* spp. Based on core and pan-genome analysis as well as nucleotide diversity calculation, this genus displayed a considerable diversity on the genome scale. In line with the MLSA-based phylogenetic analysis, the extension of the core genome increased when we considered the genomic regions shared amongst the *R*. *opacus* strains; however, the highest number of shared genomic regions was in R7 and RHA1. Moreover, only a limited number of genomic regions was shared by BCP1 and SB3094, despite their phylogenetic relation. These results suggest that the extension of genomic regions shared by the two strains might be related to the adaptation to specific niches (both R7 and RHA1 were isolated from soils contaminated with aromatic compounds) more than to their taxonomic correlation. As a result of the genome comparison, the regions uniquely found in BCP1 and R7 largely included genes coding for proteins with unknown functions. Other genes found in these regions coded for transcriptional regulators, membrane transporters, oxidoreductases and mobile elements. Notably, most of the shared regions identified in *Rhodococcus* sp. BCP1 and *R*. *opacus* R7 were located on the chromosome, while a high percentage of unique regions was identified on the plasmids, where a large amount of mobile elements are also present. These aspects connect the occurrence of horizontal gene transfer events to the peculiar metabolic properties that characterize each strain.

In addition to genome analysis compared to other *Rhodococcus* spp., the catabolic potentials of *R*. *opacus* R7 and *Rhodococcus* sp. BCP1 were assessed towards different carbon, nitrogen, sulphur and phosphorous sources and different xenobiotic compounds. This study was aimed at both investigating the peculiar abilities of these two strains and correlating the catabolic differences of BCP1 and R7 to the different habitats from which they have been isolated (butane-growing microbial consortium able to degrade chloride aliphatic hydrocarbons and polycyclic aromatic hydrocarbon contaminated soil, respectively). With this purpose, the metabolic features of *Rhodococcus* strains were systematically analyzed using a Biolog Phenotype Microarray (PM) system with both Biolog standard plates (PM1-4, PM9-PM20) and plates manually prepared by adding different organic/xenobiotic compounds to the wells (aliphatic, alicyclic and aromatic hydrocarbons, polycyclic aromatic hydrocarbons, and carboxylated hydrocarbons, i.e. naphthenic acids). The catabolic profiles of *Rhodococcus* sp. BCP1 and *R*. *opacus* R7 were compared to define the differences between the two strains in terms of: i) carbon, nitrogen, sulphur and phosphorous source utilization (plates PM1-4); ii) metabolic response to osmolytes and to different pH growth environments (PM9-10); iii) antibiotic resistance pattern and ability to respire in presence of toxic compounds (PM11-20); iv) ability to oxidize different organic/xenobiotic compounds (plates manually prepared). Considering results from PM1-4, we found that, despite the genomic diversity, the two strains displayed similar core carbon metabolic profiles (PM1-2 plates) (Figs 3 and 4). Both strains also showed the ability to utilize a wide range of compounds as phosphorous sources (PM4 wells A1-E12); while a great difference was shown in terms of nitrogen and sulphur compound assimilation (PM3 and PM4 wells F1-H12). In particular, R7 showed metabolic activities on a wider variety of nitrogen and sulphur sources as compared to BCP1 (Figs 5 and 6). These results are in line with the higher amount of genes predicted to be involved in sulphur and organic nitrogen metabolism in the R7 genome (S5 Table).

Previous studies described the use of Phenotype Microarray to determine the growth capacity of *R*. *opacus* PD630 and *R*. *jostii* RHA1, although the phenotypic screening was limited to the carbon sources included in PM1-2 plates and to some nitrogen sources included in PM3 [16]. Similarly to what previously reported for *R*. *jostii* RHA1, both *R*. *opacus* R7 and *Rhodococcus* sp. BCP1 strains did not show metabolic activities on galactose. By contrast, this substrate and the derived oligogalactosides (lactose, lactitol, melibionic acid, melibiose, lactulose, raffinose, and stachyose) were efficiently utilized by *R*. *opacus* PD630. This ability was connected with the identification in PD630 of a galactose-catabolic region that was not found in RHA1. In the present work, in line with the inability of BCP1 and R7 strains to grow on galactose and derivatives, the galactose-catabolic region detected in PD630 was not found in both the genomes under analysis. Like *R*. *opacus* PD630, *R*. *opacus* R7 was able to utilize D-galactonic acid-γ-lactone that was not degraded by BCP1 and RHA1, suggesting this catabolic capacity to be specific of strains belonging to *R*. *opacus* species.

The effects of different osmolites, pH ranges and toxicants on the two *Rhodococcus* spp. strains were tested by using plates PM9-PM20. Interestingly, in contrast to BCP1, R7 showed a general resistance to low pH in the presence of several amino acids (S2 and S3 Figs), probably due to the activity of decarboxylases that generate alkaline amines by the catabolism of these compounds [20]. In response to the presence of osmolytes, R7 could tolerate high concentrations of NaCl, sodium formate, and sodium lactate compared with BCP1, while BCP1 could resist to high concentrations of urea that vice versa inhibited the R7 activity. Both strains showed wide antibiotic resistance patterns including several antibiotics, metals (except for vanadate salts) along with antiseptics, detergents and anti-microbial agents (S4, S5 and S6 Figs). The sensitivity shown by R7 and BCP1 towards macrolides, rifampin, vancomycin, novobiocin is in line with what previously described for *R*. *equi* [54–56]. R7 and BCP1 strains also showed sensitivity to antibiotics known to inhibit Gram-positive microorganisms like fusidic acid, pheneticillin, and to alkaloids with anti-lipase activity in *Candida rugosa* like sanguinarine and cheleritrine [57]. BCP1 differs from R7 for its ability to grow on the anti-microbial agents ethionamide and on thallium(I) acetate.

Considering the genetic aspects related to the phenotypic features observed with plates PM9-PM20, around 140 and 180 genes were annotated in the genomes of BCP1 and R7, respectively, that are involved in various stress responses (osmotic stress, detoxification, heat/cold shock, oxidative stress) (S5 Table). The resistance to stress can also be correlated to the features of *Rhodococcus* cell wall that contains high-molecular weight α-alkyl-β-hydroxy fatty acids named as mycolic acids. Indeed, mycolic acids are known to confer resistance to chemical injury, low permeability to hydrophobic antibiotics and to hydrophilic substrates, and resistance to dehydration [58]. Phenotype Microarray system was also used to test the catabolic capacity of BCP1 and R7 strains towards different categories of xenobiotic compounds belonging to: aliphatic hydrocarbons (i.e.decanoic acid, hexanoic acid), polycyclic aromatic hydrocarbons (PAHs), BTEX and other aromatic compounds (i.e. dibenzothiophene a recalcitrant component of fossil fuels), and carboxylated compounds (i.e. naphthenic acids). Both strains showed the ability to utilize a wide range of these compounds (Fig 7). Concerning *n*-alkanes, BCP1 and R7 could grow on *n*-alkanes with aliphatic chain >C_12_. These metabolic capacities were described previously using only a few of the *n*-alkanes tested in the present work [25, 29]. Here we report a series of new findings on R7 and BCP1 *n*-alkanes metabolism, namely: i) R7 has lower activities than BCP1 on odd-carbon *n*-alkanes (C_13_ and C_17_) as compared to even-carbon *n*-alkanes; ii) BCP1 has the ability to utilize tetracontane (C_34_) and hexatriacontane (C_36_) for growth; further, unlike R7, BCP1 could grow on *n*-alkanes ranging from *n*-hexane (C_6_) to *n*-nonane (C_9_). Both strains could grow on recalcitrant alicyclic hydrocarbon, cyclohexane, and on the intermediate of the cyclohexane degradation pathway [59] as cyclohexanone, although these substrates were preferentially utilized by BCP1. Amongst the naphthenic acids, R7 and BCP1 could grow and showed high activities only on cyclohexanecarboxylic acid (CHCA) and on 1,4-cyclohexanedicarboxylic acid (1,4-CHdiCA). Unlike R7, BCP1 could also grow on cyclopentanecarboxylic acid (CPCA) confirming the limited capacity of R7 to utilize odd-number chain alkanes. R7 and BCP1 strains showed a certain level of metabolic activity on trans-1,2-cyclohexanedicarboxylic acid (trans-ChdiCA), 3-methyl-1-cyclohexanecarboxylic acid (mCHCA), 4-phenylbutyric acid; while none of them could utilize the more complex molecules such as cyclohexane butyric acid (CHBA), adamantane carboxylic acid (ACA), cyclohexane acetic acid (CHAA), 1,2,3,4-Tetrahydro-2-naphthoic acid (THNA) under these growth conditions.

Notably, R7 showed its highest metabolic activity on dibenzothiophene (DBT), a fuel contaminant naturally present in crude oil, that can be used by some bacterial species mainly from the genus *Rhodococcus*, as a sulphur source [60].

A genome analysis was performed to identify gene clusters involved in the metabolism of contaminants tested in Phenotype Microarray assays. In this work, we show that *R*. *opacus* R7 contains multiple genes for the degradation of a large set of aromatic and polyaromatic hydrocarbons, while a lower variability in terms of genes predicted to be involved in aromatic degradation was shown by BCP1 strain. These genetic features can be related to the strong genetic pressure exerted by the specific carbon sources available in the different niches from which each of the two strains was isolated. Indeed, R7 was isolated from a soil contaminated with aromatic hydrocarbons, while BCP1 was isolated from a butane-utilizing consortium enriched in short-chain aliphatic hydrocarbons. According to this, the *smo* gene cluster, previously described to code for a soluble di-iron monooxygenase involved in aliphatic chain hydrocarbon degradation [28] is included in one of the unique regions of BCP1, while it is not conserved among the *Rhodococcus* strains compared in this study. The relative dimension of the two genomes under analysis (10 Mb for R7 and 6 Mb for BCP1) can also explain the lower degree of genetic redundancy present in BCP1 compared to R7.

In conclusion, the genome analysis of *Rhodococcus* sp. BCP1 and *R*. *opacus* R7 along with their phenotypic characterization highlighted a number of interesting features underlying the peculiar capacities of these two rhodococci for biodegradation and biotransformation applications supported by both their extraordinary genetic repertoire and environmental persistence.

## Supporting Information

S1 File Supporting Information. The S1 file contains legends.
(DOC)

S1 Fig Mauve Diagram. Whole genome sequence comparison of *R*. *opacus* R7 and *Rhodococcus* sp. BCP1 with a set of four other reference genomes: *R*. *jostii* RHA1, *R*. *opacus* PD630, *R*. *opacus* B4, *R*. *pyridinivorans* SB3094. For a global alignment of all six genomes the Mauve tool (2.3 Version) was used and the relative positions of the conserved regions found in more than one genome are presented in the same colored block.
(TIF)

S2 Fig Phenotype Microarray PM in presence of different osmolytes. Resistance differences among *R*. *opacus* R7 and *Rhodococcus* sp. BCP1 in presence of osmolytes (AI, AII, AIII). Based on activity values of phenotype microarray analysis, threshold values were established for every plates. Determined thresholds were high (green), upper middle (light green), lower middle (orange) and low (red) for high, upper middle, lower middle and low activity, respectively.
(TIFF)

S3 Fig Phenotype Microarray PM in presence of different pH values. Resistance differences among *R*. *opacus* R7 and *Rhodococcus* sp. BCP1 in presence of different pH values (AIV, AV, AVI). Based on activity values of phenotype microarray analysis, threshold values were established for every plates. Determined thresholds were high (green), upper middle (light green), lower middle (orange) and low (red) for high, upper middle, lower middle and low activity, respectively.
(TIFF)

S4 Fig Phenotype Microarray PM in presence of different antibiotics. Resistance differences among *R*. *opacus* R7 and *Rhodococcus* sp. BCP1 in presence of different antibiotics that were tested at four concentration (1, 2, 3, 4) according to Biolog procedure (AI, AII, AIII). Based on activity values of phenotype microarray analysis, threshold values were established for every plates. Determined thresholds were high (green), upper middle (light green), lower middle (orange) and low (red) for high, upper middle, lower middle and low activity, respectively.
(TIFF)

S5 Fig Phenotype Microarray PM in presence of different antiseptics. Resistance differences among *R*. *opacus* R7 and *Rhodococcus* sp. BCP1 in presence of antiseptics (AI, AII). Based on activity values of phenotype microarray analysis, threshold values were established for every plates. Determined thresholds were high (green), upper middle (light green), lower middle (orange) and low (red) for high, upper middle, lower middle and low activity, respectively.
(TIFF)

S6 Fig Phenotype Microarray PM in presence of other antiseptics and metals. Resistance differences among *R*. *opacus* R7 and *Rhodococcus* sp. BCP1 in presence of antiseptics (AIII, AIV) and metals (B). Based on activity values of phenotype microarray analysis, threshold values were established for every plates. Determined thresholds were high (green), upper middle (light green), lower middle (orange) and low (red) for high, upper middle, lower middle and low activity, respectively.
(TIFF)

S1 Table *R*. *opacus* R7 unique regions deriving from the comparative genome alignments with *Rhodococcus* sp. BCP1 and *Rhodococcus* sp. BCP1 unique regions deriving from the comparative genome alignments with *R*. *opacus* R7. (XLSX)

S2 Table Enzymatic class identification for xenobiotic degradation in the unique regions of *Rhodococcus* sp. BCP1 and *R*. *opacus* R7 genomes. (PDF)

S3 Table Activity values of tested substrates in Phenotype Microarray analysis in presence of *R*. *opacus* R7 and *Rhodococcus* sp. BCP1. Activity values in presence of carbon sources: carbohydrates (AI, AII), carboxylic acids (BI, BII), alcohols, amides, amines, esters, fatty acids, polymers (C), amino acids (D); nitrogen sources (AI, AII, AIII); phosphorous sources (AI, AII); sulphur sources (B); osmolytes (AI, AII, AIII) and pH values (AIV, AV, AVI); antibiotics (AI, AII, AIII); antiseptics (AI, AII, AIII, AIV) and metals (B).
(XLS)

S4 Table Enzymatic class for fatty acids β-oxidation. (PDF)

S5 Table RAST subsystem categories of *R*. *opacus* R7 and *Rhodococcus* sp. BCP1 metabolism in presence of carbon, nitrogen, phosphorous and sulphur sources. (PDF)

S6 Table Activity values of tested organic/xenobiotic compounds in Phenotype Microarray analysis in presence of *R*. *opacus* R7 and *Rhodococcus* sp. BCP1 (see Fig 7). (XLS)

S7 Table Comparison of predicted genes and proteins of *alk* cluster of *R*. *opacus* R7 and *Rhodococcus* sp. BCP1 and comparison with *R*. *jostii* RHA1 homologous proteins (see Fig 8). (PDF)

S8 Table Comparison of predicted genes and proteins of *prm* cluster of *R*. *opacus* R7 and *Rhodococcus* sp. BCP1 and comparison with *R*. *jostii* RHA1 homologous proteins (see Fig 8). (PDF)

S9 Table Comparison of predicted genes and proteins of *akb* cluster of *R*. *opacus* R7 and *Rhodococcus* sp. BCP1 and comparison with *R*. *jostii* RHA1 homologous proteins (see Fig 8). (PDF)

S10 Table Comparison of predicted genes and proteins of *dsz* cluster of *R*. *opacus* R7 and *Rhodococcus* sp. BCP1 and comparison with *R*. *jostii* RHA1 homologous proteins (see Fig 8). (PDF)

S11 Table Comparison of predicted genes and proteins of *nar* cluster of *R*. *opacus* R7 and *Rhodococcus* sp. BCP1 and comparison with *R*. *jostii* RHA1 homologous proteins (see Fig 9). (PDF)

S12 Table Comparison of predicted genes and proteins of *gen* cluster of *R*. *opacus* R7 and *Rhodococcus* sp. BCP1 and comparison with *R*. *jostii* RHA1 homologous proteins (see Fig 9). (PDF)

S13 Table Comparison of predicted genes and proteins of *bph* cluster of *R*. *opacus* R7 and *Rhodococcus* sp. BCP1 and comparison with *R*. *jostii* RHA1 homologous proteins (see Fig 9). (PDF)

S14 Table Comparison of predicted genes and proteins of naphthenic acids cluster of *R*. *opacus* R7 and *Rhodococcus* sp. BCP1 and comparison with *R*. *jostii* RHA1 homologous proteins (see Fig 10). (PDF)

S15 Table Comparison of predicted genes and proteins of *cat* cluster of *R*. *opacus* R7 and *Rhodococcus* sp. BCP1 and comparison with *R*. *jostii* RHA1 homologous proteins (see Fig 11). (PDF)

S16 Table Comparison of predicted genes and proteins of *pca* cluster of *R*. *opacus* R7 and *Rhodococcus* sp. BCP1 and comparison with *R*. *jostii* RHA1 homologous proteins (see Fig 11). (PDF)

S17 Table Comparison of predicted genes and proteins of *paa* cluster of *R*. *opacus* R7 and *Rhodococcus* sp. BCP1 and comparison with *R*. *jostii* RHA1 homologous proteins (see Fig 11). (PDF)

S18 Table Comparison of predicted genes and proteins of *hmg* cluster of *R*. *opacus* R7 and *Rhodococcus* sp. BCP1 and comparison with *R*. *jostii* RHA1 homologous proteins (see Fig 11). (PDF)

All the files of supporting informations can be downloaded from the database at www.itb.cnr.it/rhodococcus
