# Supplementary material for: Genome and Phenotype Microarray Analyses of Rhodococcus sp. BCP1 and Rhodococcus opacus R7: Genetic Determinants and Metabolic Abilities with Environmental Relevance
Source: PLoS One. 2015 Oct 1;10(10):e0139467. doi: 10.1371/journal.pone.0139467 (PMC4591350; doi:10.1371/journal.pone.0139467)
Supplement: S1 File — The S1 file contains legends. (DOC) [file pone.0139467.s001.doc]

**SUPPORTING INFORMATION**

**S1 Fig. Mauve Diagram.** Whole genome sequence comparison of *R. opacus* R7 and *Rhodococcus* sp. BCP1 with a set of four other reference genomes: *R*. *jostii* RHA1, *R*. *opacus* PD630, *R*. *opacus* B4, *R*. *pyridinivorans* SB3094. For a global alignment of all six genomes the Mauve tool (2.3 Version) was used and the relative positions of the conserved regions found in more than one genome are presented in the same colored block.

**S2 Fig. Phenotype Microarray PM in presence of different osmolytes.** Resistance differences among *R*. *opacus* R7 and *Rhodococcus* sp. BCP1 in presence of osmolytes (AI, AII, AIII). Based on activity values of phenotype microarray analysis, threshold values were established for every plates. Determined thresholds were high (green), upper middle (light green), lower middle (orange) and low (red) for high, upper middle, lower middle and low activity, respectively.

**S3 Fig. Phenotype Microarray PM in presence of different pH values.** Resistance differences among *R*. *opacus* R7 and *Rhodococcus* sp. BCP1 in presence of different pH values (AIV, AV, AVI). Based on activity values of phenotype microarray analysis, threshold values were established for every plates. Determined thresholds were high (green), upper middle (light green), lower middle (orange) and low (red) for high, upper middle, lower middle and low activity, respectively.

**S4 Fig. Phenotype Microarray PM in presence of different antibiotics.** Resistance differences among *R*. *opacus* R7 and *Rhodococcus* sp. BCP1 in presence of different antibiotics that were tested at four concentration (1, 2, 3, 4) according to Biolog procedure (AI, AII, AIII). Based on activity values of phenotype microarray analysis, threshold values were established for every plates. Determined thresholds were high (green), upper middle (light green), lower middle (orange) and low (red) for high, upper middle, lower middle and low activity, respectively.

**S5 Fig. Phenotype Microarray PM in presence of different antiseptics.** Resistance differences among *R*. *opacus* R7 and *Rhodococcus* sp. BCP1 in presence of antiseptics (AI, AII). Based on activity values of phenotype microarray analysis, threshold values were established for every plates. Determined thresholds were high (green), upper middle (light green), lower middle (orange) and low (red) for high, upper middle, lower middle and low activity, respectively.

**S6 Fig. Phenotype Microarray PM in presence of other antiseptics and metals.** Resistance differences among *R*. *opacus* R7 and *Rhodococcus* sp. BCP1 in presence of antiseptics (AIII, AIV) and metals (B). Based on activity values of phenotype microarray analysis, threshold values were established for every plates. Determined thresholds were high (green), upper middle (light green), lower middle (orange) and low (red) for high, upper middle, lower middle and low activity, respectively.

**S1 Table.** *R. opacus* R7 unique regions deriving from the comparative genome alignments with *Rhodococcus* sp. BCP1 and *Rhodococcus* sp. BCP1 unique regions deriving from the comparative genome alignments with *R. opacus* R7**.**

**S2 Table.** Enzymatic class identification for xenobiotic degradation in the unique regions of *Rhodococcus* sp. BCP1 and *R. opacus* R7 genomes.

**S3 Table.** Activity values of tested substrates in Phenotype Microarray analysis in presence of *R*. *opacus* R7 and *Rhodococcus* sp. BCP1. Activity values in presence of carbon sources: carbohydrates (AI, AII), carboxylic acids (BI, BII), alcohols, amides, amines, esters, fatty acids, polymers (C), amino acids (D); nitrogen sources (AI, AII, AIII); phosphorous sources (AI, AII); sulphur sources (B); osmolytes (AI, AII, AIII) and pH values (AIV, AV, AVI); antibiotics (AI, AII, AIII); antiseptics (AI, AII, AIII, AIV) and metals (B).

**S4 Table.** Enzymatic class for fatty acids β-oxidation.

**S5 Table.** RAST subsystem categories of *R*. *opacus* R7 and *Rhodococcus* sp. BCP1 metabolism in presence of carbon, nitrogen, phosphorous and sulphur sources.

**S6 Table.** Activity values of tested organic/xenobiotic compounds in Phenotype Microarray analysis in presence of *R*. *opacus* R7 and *Rhodococcus* sp. BCP1 (see Fig. 7).

**S7 Table.** Comparison of predicted genes and proteins of *alk* cluster of *R*. *opacus* R7 and *Rhodococcus* sp. BCP1 and comparison with *R*. *jostii* RHA1 homologous proteins (see Fig. 8).

**S8 Table.** Comparison of predicted genes and proteins of *prm* cluster of *R*. *opacus* R7 and *Rhodococcus* sp. BCP1 and comparison with *R*. *jostii* RHA1 homologous proteins (see Fig. 8).

**S9 Table.** Comparison of predicted genes and proteins of *akb* cluster of *R*. *opacus* R7 and *Rhodococcus* sp. BCP1 and comparison with *R*. *jostii* RHA1 homologous proteins (see Fig. 8).

**S10 Table.** Comparison of predicted genes and proteins of *dsz* cluster of *R*. *opacus* R7 and *Rhodococcus* sp. BCP1 and comparison with *R*. *jostii* RHA1 homologous proteins (see Fig. 8).

**S11 Table.** Comparison of predicted genes and proteins of *nar* cluster of *R*. *opacus* R7 and *Rhodococcus* sp. BCP1 and comparison with *R*. *jostii* RHA1 homologous proteins (see Fig. 9).

**S12 Table.** Comparison of predicted genes and proteins of *gen* cluster of *R*. *opacus* R7 and *Rhodococcus* sp. BCP1 and comparison with *R*. *jostii* RHA1 homologous proteins (see Fig. 9).

**S13 Table.** Comparison of predicted genes and proteins of *bph* cluster of *R*. *opacus* R7 and *Rhodococcus* sp. BCP1 and comparison with *R*. *jostii* RHA1 homologous proteins (see Fig. 9).

**S14 Table.** Comparison of predicted genes and proteins of naphthenic acids cluster of *R*. *opacus* R7 and *Rhodococcus* sp. BCP1 and comparison with *R*. *jostii* RHA1 homologous proteins (see Fig. 10).

**S15 Table.** Comparison of predicted genes and proteins of *cat* cluster of *R*. *opacus* R7 and *Rhodococcus* sp. BCP1 and comparison with *R*. *jostii* RHA1 homologous proteins (see Fig. 11).

**S16 Table.** Comparison of predicted genes and proteins of *pca* cluster of *R*. *opacus* R7 and *Rhodococcus* sp. BCP1 and comparison with *R*. *jostii* RHA1 homologous proteins (see Fig. 11).

**S17 Table.** Comparison of predicted genes and proteins of *paa* cluster of *R*. *opacus* R7 and *Rhodococcus* sp. BCP1 and comparison with *R*. *jostii* RHA1 homologous proteins (see Fig. 11).

**S18 Table.** Comparison of predicted genes and proteins of *hmg* cluster of *R*. *opacus* R7 and *Rhodococcus* sp. BCP1 and comparison with *R*. *jostii* RHA1 homologous proteins (see Fig. 11).
