# Supplementary figures and images for: Genome and Phenotype Microarray Analyses of Rhodococcus sp. BCP1 and Rhodococcus opacus R7: Genetic Determinants and Metabolic Abilities with Environmental Relevance
Source: PLoS One. 2015 Oct 1;10(10):e0139467. doi: 10.1371/journal.pone.0139467 (PMC4591350; doi:10.1371/journal.pone.0139467)

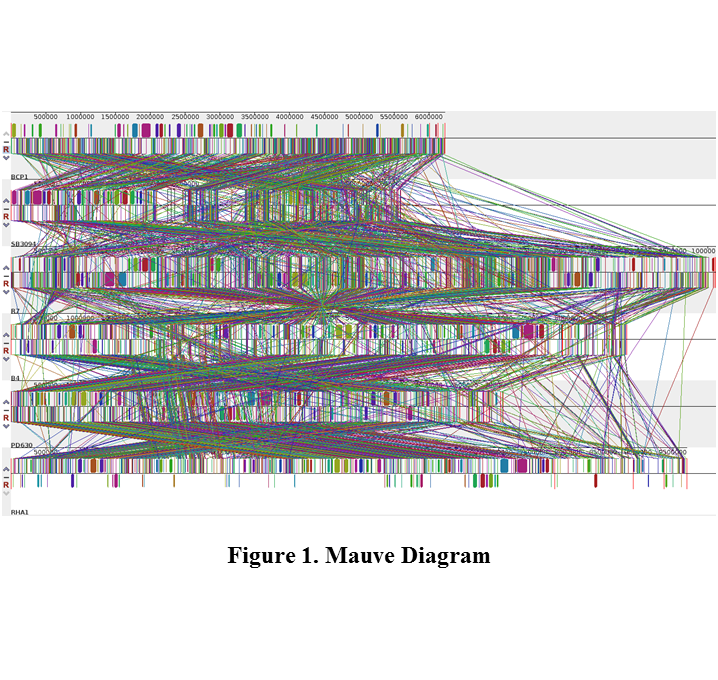

Supplement: S1 Fig — Whole genome sequence comparison of R. opacus R7 and Rhodococcus sp. BCP1 with a set of four other reference genomes: R. jostii RHA1, R. opacus PD630, R. opacus B4, R. pyridinivorans SB3094. For a global alignment of all six genomes the Mauve tool (2.3 Version) was used and the relative positions of the conserved regions found in more than one genome are presented in the same colored block. (TIF) [file pone.0139467.s002.tif]

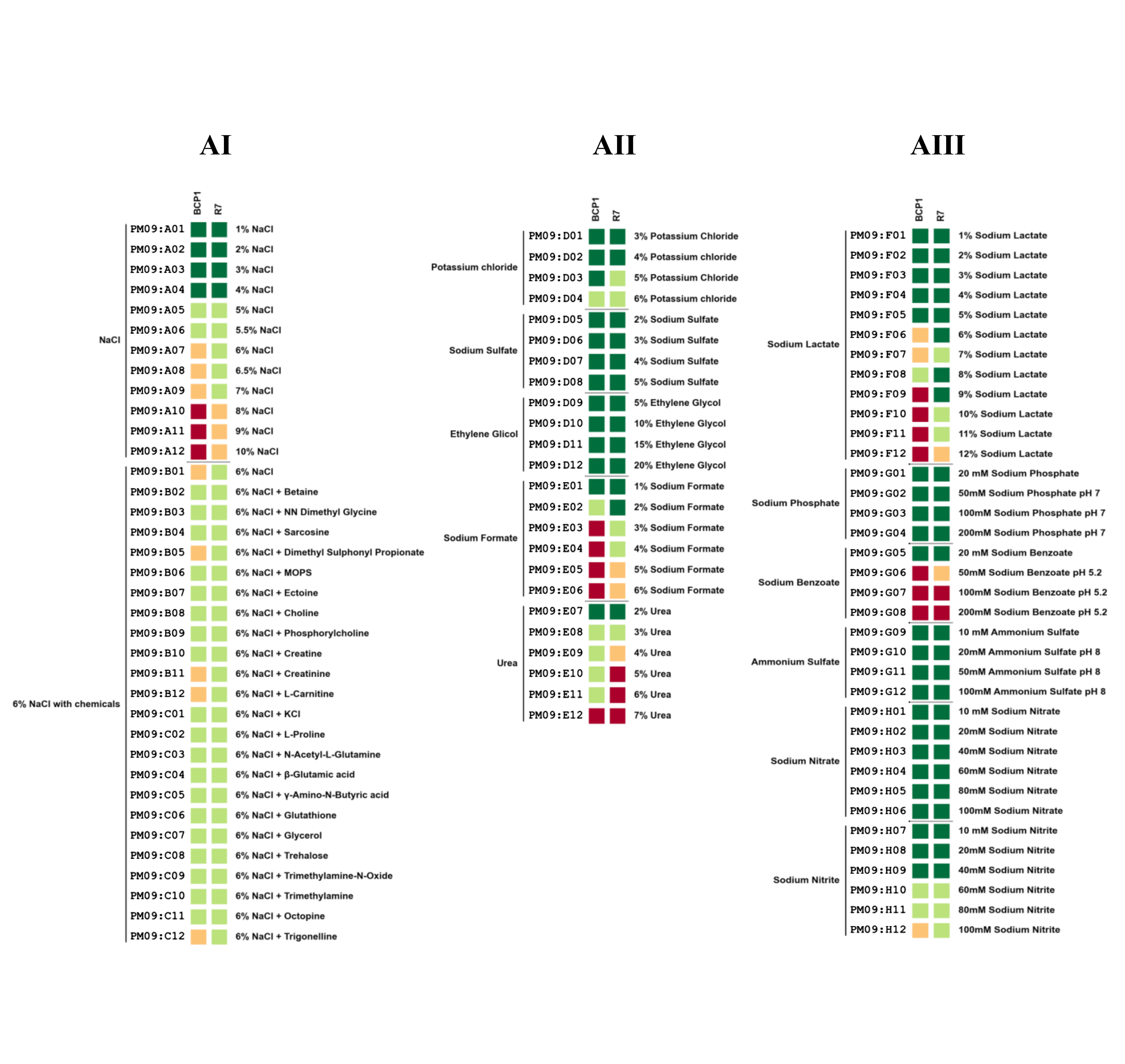

Supplement: S2 Fig — Resistance differences among R. opacus R7 and Rhodococcus sp. BCP1 in presence of osmolytes (AI, AII, AIII). Based on activity values of phenotype microarray analysis, threshold values were established for every plates. Determined thresholds were high (green), upper middle (light green), lower middle (orange) and low (red) for high, upper middle, lower middle and low activity, respectively. (TIFF) [file pone.0139467.s003.tiff]

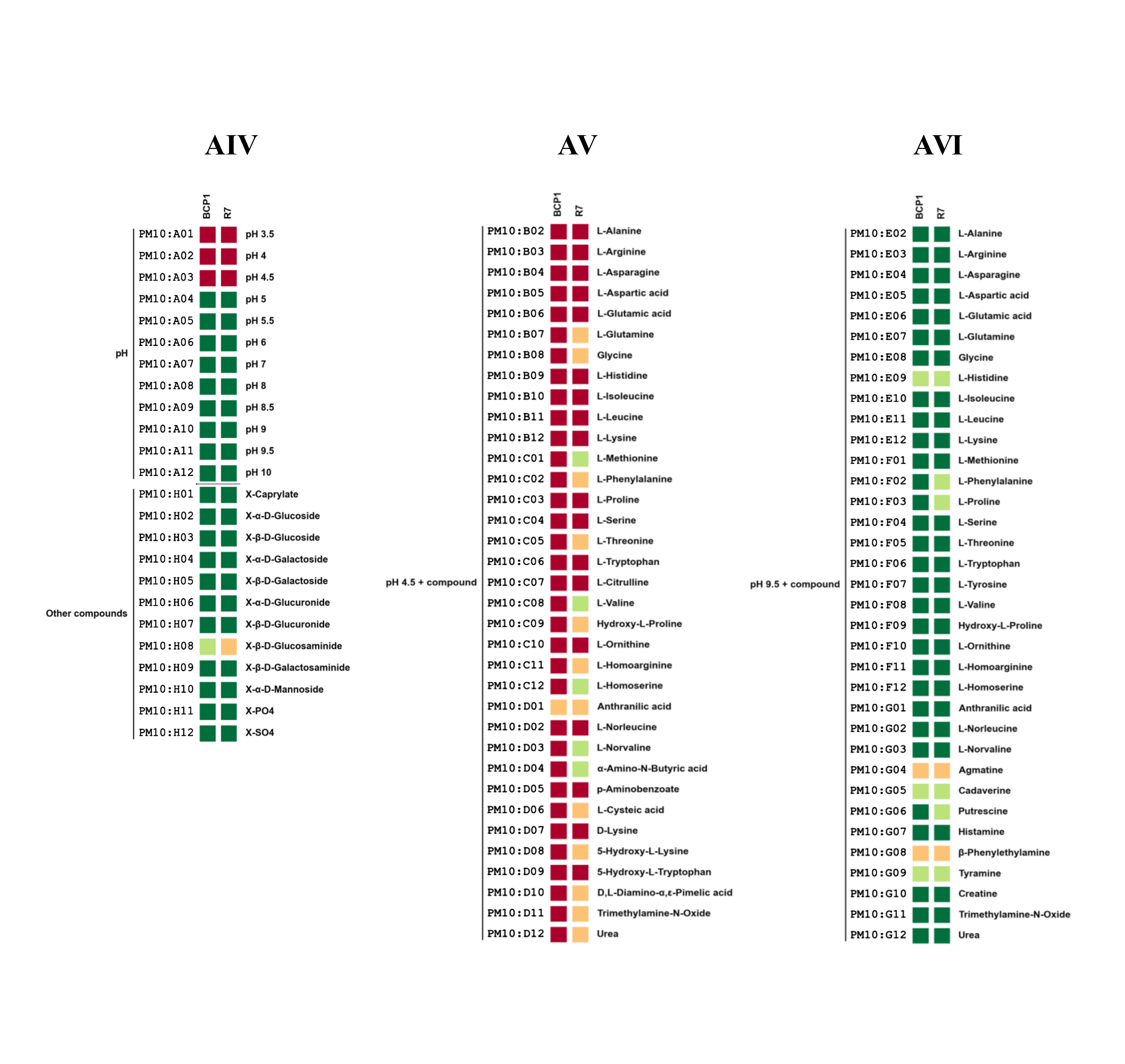

Supplement: S3 Fig — Resistance differences among R. opacus R7 and Rhodococcus sp. BCP1 in presence of different pH values (AIV, AV, AVI). Based on activity values of phenotype microarray analysis, threshold values were established for every plates. Determined thresholds were high (green), upper middle (light green), lower middle (orange) and low (red) for high, upper middle, lower middle and low activity, respectively. (TIFF) [file pone.0139467.s004.tiff]

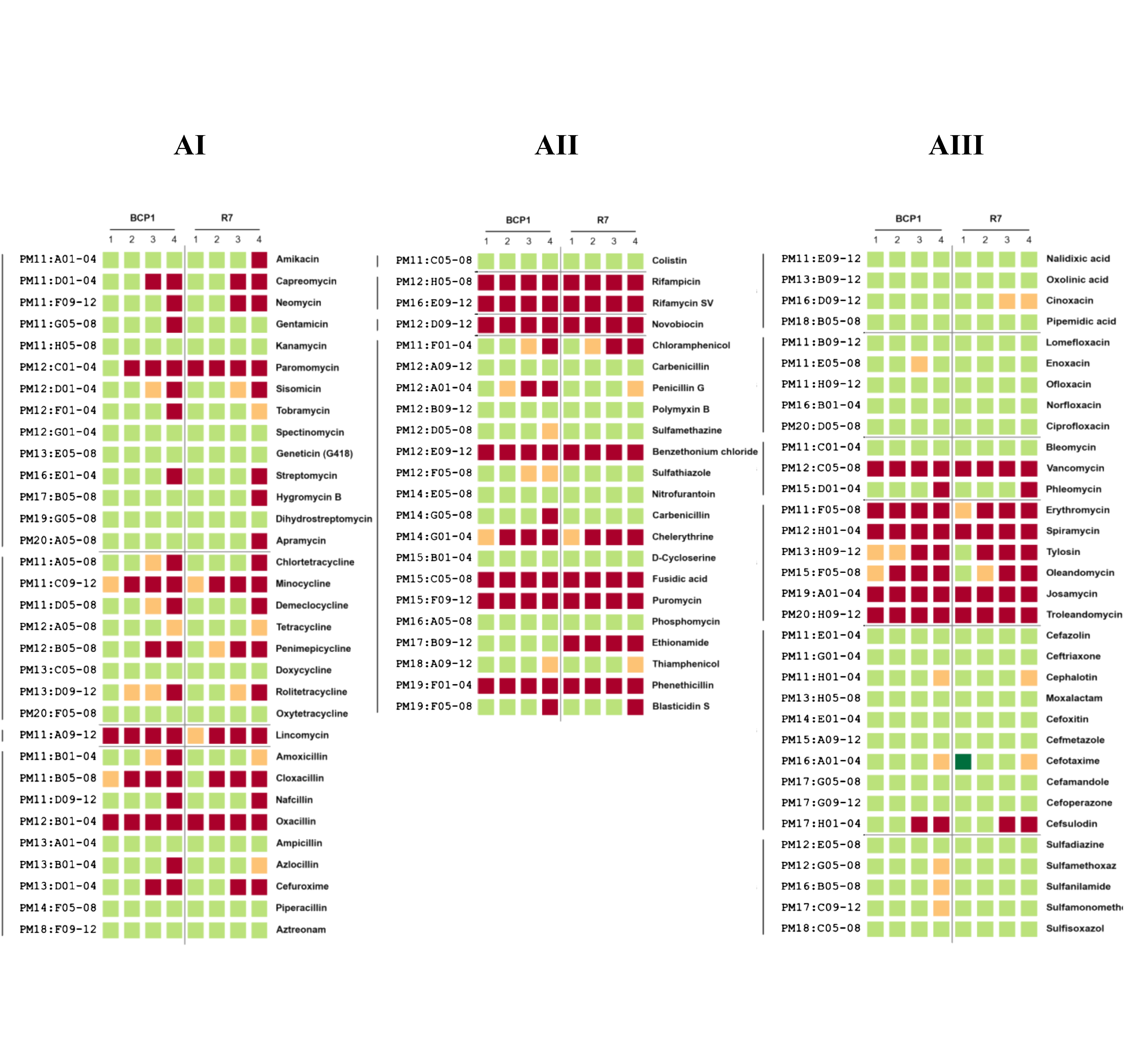

Supplement: S4 Fig — Resistance differences among R. opacus R7 and Rhodococcus sp. BCP1 in presence of different antibiotics that were tested at four concentration (1, 2, 3, 4) according to Biolog procedure (AI, AII, AIII). Based on activity values of phenotype microarray analysis, threshold values were established for every plates. Determined thresholds were high (green), upper middle (light green), lower middle (orange) and low (red) for high, upper middle, lower middle and low activity, respectively. (TIFF) [file pone.0139467.s005.tiff]

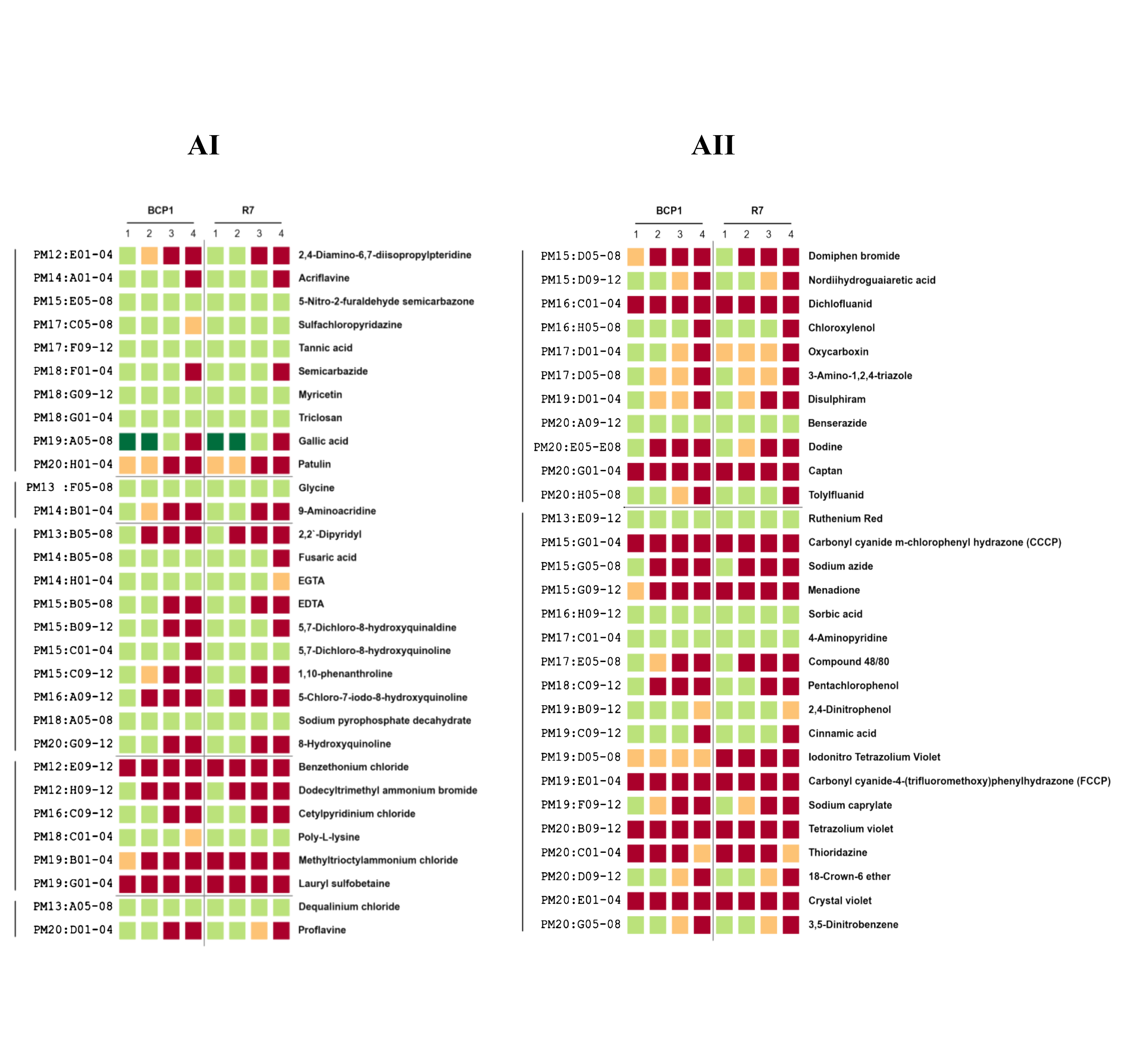

Supplement: S5 Fig — Resistance differences among R. opacus R7 and Rhodococcus sp. BCP1 in presence of antiseptics (AI, AII). Based on activity values of phenotype microarray analysis, threshold values were established for every plates. Determined thresholds were high (green), upper middle (light green), lower middle (orange) and low (red) for high, upper middle, lower middle and low activity, respectively. (TIFF) [file pone.0139467.s006.tiff]

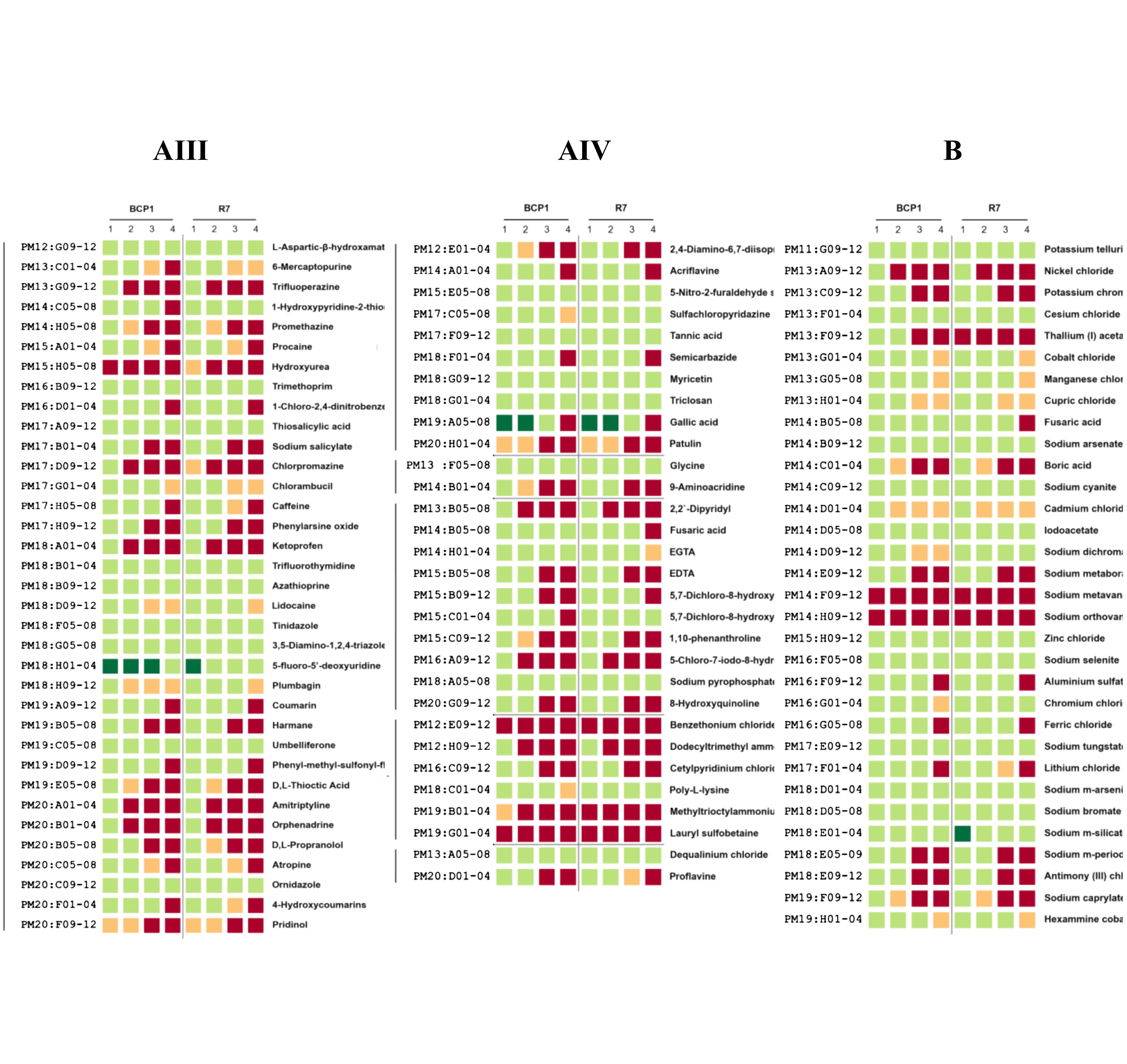

Supplement: S6 Fig — Resistance differences among R. opacus R7 and Rhodococcus sp. BCP1 in presence of antiseptics (AIII, AIV) and metals (B). Based on activity values of phenotype microarray analysis, threshold values were established for every plates. Determined thresholds were high (green), upper middle (light green), lower middle (orange) and low (red) for high, upper middle, lower middle and low activity, respectively. (TIFF) [file pone.0139467.s007.tiff]
