# Supplementary material for: Genome and Phenotype Microarray Analyses of Rhodococcus sp. BCP1 and Rhodococcus opacus R7: Genetic Determinants and Metabolic Abilities with Environmental Relevance
Source: PLoS One. 2015 Oct 1;10(10):e0139467. doi: 10.1371/journal.pone.0139467 (PMC4591350; doi:10.1371/journal.pone.0139467)
Supplement: S2 Table — (PDF) [file pone.0139467.s009.pdf]

**Key functions**	***R. opacus* R7**	***R.* sp. BCP1**
**oxygenase**	10	12
**hydroxylase**	2	3
**dehydrogenase**	20	46
**hydrolase**	26	10
**oxidoreductase**	12	14
**ligase**	4	13
**isomerase**	4	3
**aldolase**	1	2
**ion-transporting ATPase**	/	4
**P450 cytochrome**	2	7
