# Supplementary material for: Genome and Phenotype Microarray Analyses of Rhodococcus sp. BCP1 and Rhodococcus opacus R7: Genetic Determinants and Metabolic Abilities with Environmental Relevance
Source: PLoS One. 2015 Oct 1;10(10):e0139467. doi: 10.1371/journal.pone.0139467 (PMC4591350; doi:10.1371/journal.pone.0139467)
Supplement: S4 Table — (PDF) [file pone.0139467.s011.pdf]

Gene products for b-oxidation	Number of genes		Reactions
	BCP1	R7	
3-hydroxyacyl-CoA dehydrogenase (EC 1.1.1.35)	7	20	(S)-3-hydroxyacyl-CoA -----> oxoacyl-CoA +
3-ketoacyl-CoA thiolase (EC 2.3.1.16)	20	33	Acyl-CoA + acetyl-CoA ----> CoA + 3-oxoacyl-CoA.
Enoyl-CoA hydratase (EC 4.2.1.17)	30	61	(3S)-3-hydroxyacyl-CoA ----> trans-2(or 3)-enoyl-CoA + H2O
Long-chain-fatty-acid--CoA ligase (EC 6.2.1.3)	75	58	ATP + a long-chain fatty acid + CoA ----> AMP + diphosphate + acyl-CoA
3-hydroxybutyryl-CoA epimerase (EC 5.1.2.3)	3	5	(S)-3-hydroxybutanoyl-CoA ---> (R)-3-hydroxybutanoyl-CoA
