# Supplementary material for: Genome and Phenotype Microarray Analyses of Rhodococcus sp. BCP1 and Rhodococcus opacus R7: Genetic Determinants and Metabolic Abilities with Environmental Relevance
Source: PLoS One. 2015 Oct 1;10(10):e0139467. doi: 10.1371/journal.pone.0139467 (PMC4591350; doi:10.1371/journal.pone.0139467)
Supplement: S5 Table — (PDF) [file pone.0139467.s012.pdf]

**Subsystem Category Distribution**	R7	Bcp1
**Carbohydrates**	1354	623
Central carbohydratemetabolism	530	206
MethylglyoxalMetabolism	(165)	(49)
Pyruvate:ferredoxinoxidoreductase	-	(1)
Ethylmalonyl-CoA pathway of C2 assimilation	(8)	
Pyruvate Alanine Serine Interconversions	(17)	(4)
Glyoxylate bypass	(14)	(6)
Dihydroxyacetone kinases	(1)	-
Pyruvate metabolism II: acetyl-CoA, acetogenesis from pyruvate	(125)	(41)
Glycolysis and Gluconeogenesis	(33)	(17)
Entner-Doudoroff Pathway	(31)	(15)
Dehydrogenase complexes	(37)	(18)
TCA Cycle	(32)	(19)
Pentose phosphate pathway	(32)	(14)
Pyruvate metabolism I: anaplerotic reactions, PEP	(25)	(9)
Glycolate, glyoxylateinterconversions	(6)	(2)
Soluble methane monooxygenase (sMMO)	(4)	(8)
Ethylmalonyl-CoA pathway of C2 assimilation	-	(3)
Aminosugars	8	-
Chitin and N-acetylglucosamine utilization	(8)	-
Di- and oligosaccharides	50	49
Lactose and Galactose Uptake and Utilization	(10)	(8)
Trehalose Biosynthesis	(15)	(19)
Maltose and Maltodextrin Utilization	(18)	(18)
Trehalose Uptake and Utilization	(5)	-
Lactose utilization	(2)	(2)
Glycoside hydrolases	-	-
One-carbon Metabolism	144	90
Formaldehyde assimilation: Ribulose monophosphate pathway	(4)	-
Serine-glyoxylate cycle	(129)	(83)
One-carbon metabolism by tetrahydropterines	(10)	(5)
Methanogenesis	(1)	(2)
Organic acids	38	13
Methylcitrate cycle	(5)	-
Propionate-CoA to Succinate Module	(7)	-
Glycerate metabolism	(18)	(5)
Lactate utilization	(8)	(8)
Fermentation	362	193
Butanol Biosynthesis	(146)	(77)
Fermentations: Mixed acid	(44)	(24)
Acetolactate synthase subunits	(9)	(4)
Fermentations: Lactate	(8)	(6)

Acetyl-CoA fermentation to Butyrate	(139)	(71)
Acetoin, butanediol metabolism	(16)	(11)
Sugar alcohols	53	20
Glycerol and Glycerol-3-phosphate Uptake and Utilization	(18)	(11)
Inositol catabolism	(35)	(9)
Polysaccharides	27	26
Glycogen metabolism	(8)	(8)
Alpha-Amylase locus in Streptocococcus	(19)	(18)
Monosaccharides	142	26
Mannose Metabolism	(6)	(4)
Deoxyribose and Deoxynucleoside Catabolism	(6)	(4)
D-gluconate and ketogluconates metabolism	(20)	(2)
D-galactarate, D-glucarate and D-glycerate catabolism	(14)	-
Fructose utilization	(10)	(10)
L-rhamnose utilization	(15)	-
D-Galacturonate and D-Glucuronate Utilization	(20)	-
D-galactonate catabolism	(7)	-
D-ribose utilization	(22)	(4)
Xylose utilization	(9)	(2)
D-galactarate, D-glucarate and D-glycerate catabolism	(13)	-
**Amino Acids and Derivatives**	1108	565
Glutamine, glutamate, aspartate, asparagine; ammoniaassimilation	76	33
Glutamine, Glutamate, Aspartate and Asparagine Biosynthesis	(52)	(26)
Glutamatedehydrogenases	(14)	(4)
Glutaminesynthetases	(5)	(3)
Glutamate and Aspartate uptake in Bacteria	(5)	-
Histidine Metabolism	26	15
Histidine Degradation	(13)	(4)
Histidine Biosynthesis	(13)	(11)
Arginine; urea cycle, polyamines	123	58
Putrescine utilization pathways	(13)	(4)
Polyamine Metabolism	(19)	(6)
Arginine and Ornithine Degradation	(18)	(9)
Arginine Biosynthesis	(12)	(10)
Urea carboxylase and Allophanate hydrolase cluster	(18)	(5)
Arginine Deiminase Pathway	(5)	(3)
Urease subunits	(6)	-
Arginine Biosynthesis extended	(12)	(10)
Urea decomposition	(20)	(11)
Lysine, threonine, methionine, and cysteine	181	107
Methionine Biosynthesis	(46)	(27)
Threonine degradation	(17)	(7)
Lysine Biosynthesis DAP Pathway, GJO scratch	(18)	(13)

Methionine Degradation	(34)	(15)
Threonine and Homoserine Biosynthesis	(17)	(10)
Cysteine Biosynthesis	(25)	(21)
Lysine Biosynthesis DAP Pathway	(18)	(13)
Lysine degradation	(6)	(1)
Amino Acids and Derivatives - no subcategory	21	7
Creatine and Creatinine Degradation	(21)	(7)
Branched-chain amino acids	435	205
Isoleucine degradation	(157)	(77)
Leucine Degradation and Hydroxymethylglutaryl-CoA Metabolism	(75)	(34)
Branched-Chain Amino Acid Biosynthesis	(32)	(17)
Hydroxymethylglutaryl CoA Synthesis	(42)	(17)
Valine degradation	(117)	(54)
Leucine Biosynthesis	(12)	(6)
Aromatic amino acids and derivatives	116	81
Common Pathway For Synthesis of Aromatic Compounds (DAHP synthase to chorismate)	(11)	(11)
Aromatic amino acid interconversions with aryl acids	(3)	(1)
Chorismate Synthesis	(18)	(20)
Chorismate: Intermediate for synthesis of Tryptophan, PAPA antibiotics, PABA, 3-hydroxyanthranilate and more	(20)	(19)
Phenylalanine and Tyrosine Branches from Chorismate	(6)	(10)
Tryptophan synthesis	(15)	(11)
Aromatic amino acid degradation	(25)	(9)
Proline and 4-hydroxyproline	41	11
Proline Synthesis	(8)	(4)
Hypothetical Proteins Related to Proline Metabolism	(2)	(2)
Proline, 4-hydroxyproline uptake and utilization	(31)	(5)
Alanine, serine, and glycine	107	48
Glycine Biosynthesis	(4)	(1)
Alanine biosynthesis	(15)	(15)
Serine Biosynthesis	(29)	(8)
Glycine cleavage system	(9)	(6)
Glycine and Serine Utilization	(50)	(18)
**Nitrogen Metabolism**	73	38
Nitrogen Metabolism - no subcategory	73	34
Allantoin Utilization	(11)	(4)
Nitric oxide synthase	(26)	-
Nitrosative stress	(1)	(1)
Amidase clustered with urea and nitrile hydratase functions	(2)	
Nitrate and nitrite ammonification	(10)	(11)
Ammonia assimilation	(23)	(18)
**SulfurMetabolism**	120	58

Inorganicsulfurassimilation	40	27
InorganicSulfurAssimilation	(40)	(27)
SulfurMetabolism - no subcategory	23	16
Thioredoxin-disulfide reductase	(18)	(14)
Galactosylceramide and Sulfatide metabolism	(5)	(2)
Organic sulfur assimilation	57	15
Sulfate assimilation related cluster	(6)	-
Utilization of glutathione as a sulphur source	(4)	(3)
Alkanesulfonate assimilation	(33)	(8)
Alkanesulfonates Utilization	(14)	(4)
**Phosphorus Metabolism**	43	32
Phosphorus Metabolism - no subcategory	43	32
High affinity phosphate transporter and control of PHO regulon	(13)	(9)
Phosphate metabolism	(26)	(19)
Polyphosphate	(4)	(4)
**Nucleosides and Nucleotides**	166	102
Pyrimidines	47	35
pyrimidine conversions	(32)	(25)
De Novo Pyrimidine Synthesis	(15)	(10)
Purines	79	44
De Novo Purine Biosynthesis	(16)	(15)
Xanthine dehydrogenase subunits	(10)	-
Xanthine Metabolism in Bacteria	(1)	(1)
A hypothetical coupled to de Novo Purine Biosynthesis	(4)	-
Purine Utilization	(21)	(7)
Purine conversions	(27)	(21)
Nucleosides and Nucleotides - no subcategory	27	14
Ribonucleotide reduction	(6)	(6)
Hydantoin metabolism	(18)	(5)
Adenosylnucleosidases	(3)	(3)
Detoxification	13	9
Nucleoside triphosphate pyrophosphohydrolaseMazG	(1)	(1)
Nudix proteins (nucleoside triphosphate hydrolases)	(9)	(5)
Housecleaning nucleoside triphosphate pyrophosphatases	(3)	(3)
**Aminosugars Fatty Acids, Lipids, and Isoprenoids**	712	415
Phospholipids	152	63
Glycerolipid and Glycerophospholipid Metabolism in Bacteria	(152)	(63)
Triacylglycerols	(5)	(6)
Triacylglycerol metabolism	(5)	(6)
Fatty acids	307	209

Fatty Acid Biosynthesis FASII	(121)	(63)
Fatty acid metabolism cluster	(180)	(137)
Acyl-CoA thioesterase II	(6)	(9)
Fatty Acids, Lipids, and Isoprenoids - no subcategory	187	101
Polyhydroxybutyrate metabolism	(187)	101)
Isoprenoids	61	36
Isoprenoinds for Quinones	(5)	-
Isoprenoid Biosynthesis	(44)	(29)
PolyprenylDiphosphate Biosynthesis	(4)	-
Nonmevalonate Branch of Isoprenoid Biosynthesis	(7)	(7)
Isoprenoid Biosynthesis: Interconversions	(1)	-
**Metabolism of Aromatic Compounds**	268	128
Peripheral pathways for catabolism of aromatic compounds	71	17
Salicylate ester degradation	(8)	(2)
Quinate degradation	(1)	(1)
Biphenyl Degradation	(30)	-
Benzoate degradation	(21)	(12)
p-Hydroxybenzoate degradation	(3)	(2)
Chloroaromatic degradation pathway	(8)	-
Anaerobic degradation of aromatic compounds	-	4
Acetophenone carboxylase 1	-	(4
)Metabolism of central aromatic intermediates	153	91
Catechol branch of beta-ketoadipate pathway	(25)	(17)
Salicylate and gentisate catabolism	(29)	(10)
Protocatechuate branch of beta-ketoadipate pathway	(30)	(24)
4-Hydroxyphenylacetic acid catabolic pathway	(16)	-
Homogentisate pathway of aromatic compound degradation	(53)	(23)
Central meta-cleavage pathway of aromatic compound degradation	-	(17)
Metabolism of Aromatic Compounds - no subcategory	44	16
Aromatic Amin Catabolism	(25)	(16)
Gentisate degradation	(19)	-
