# Supplementary material for: Genome and Phenotype Microarray Analyses of Rhodococcus sp. BCP1 and Rhodococcus opacus R7: Genetic Determinants and Metabolic Abilities with Environmental Relevance
Source: PLoS One. 2015 Oct 1;10(10):e0139467. doi: 10.1371/journal.pone.0139467 (PMC4591350; doi:10.1371/journal.pone.0139467)
Supplement: S7 Table — (PDF) [file pone.0139467.s014.pdf]

				R. opacus R7			*Rhodococcus* sp. BCP1		
**Gene**	**Homologous protein**	**Function**	**R7 vs BPC1 (aa identity)**	**R7 vs RHA1 (aa identity)**	**Position in genome**	**Accession Number**	**BCP1 vs RHA1 (aa identity)**	**Position in genome**	**Accession Number**
alkB*/*alkB1	**AlkB**	Alkane monooxygenase	80%	91%	chromosome	AIA09965.1	83%	chromosome	ADR72654.1
alkB2	**AlkB2**	Alkane monooxygenase	/	/	chromosome	/	56%	chromosome	KDE11615.1
rubA	**RubA**	Rubredoxin	79%	98%	chromosome	AIA09966.1	79%	chromosome	ADR72655.1
rubB	**RubB**	Rubredoxin	84%	97%	chromosome	AIA09967.1	81%	chromosome	ADR72656.1
rubred	**RubRed**	Rubredoxin reductase	60%	91%	chromosome	AIA09968.1	58%	chromosome	ADR72657.1
