# Supplementary material for: Genome and Phenotype Microarray Analyses of Rhodococcus sp. BCP1 and Rhodococcus opacus R7: Genetic Determinants and Metabolic Abilities with Environmental Relevance
Source: PLoS One. 2015 Oct 1;10(10):e0139467. doi: 10.1371/journal.pone.0139467 (PMC4591350; doi:10.1371/journal.pone.0139467)
Supplement: S8 Table — (PDF) [file pone.0139467.s015.pdf]

| | | | | *R. opacus* R7 | | | *Rhodococcus* sp. BCP1 | | |
|---|---|---|---|---|---|---|---|---|---|
| **Gene** | **Homologous protein** | **Function** | **R7 vs BCP1 (aa identity)** | **R7 vs RHA1 (aa identity)** | **Position in genome** | **Accession Number** | **BCP1 vs RHA1 (aa identity)** | **Position in genome** | **Accession Number** |
| ***prmA*** | PrmA | Methane monooxygenase component A alpha chain | 98% | 97% | chromosome | AII03499.1 | 98% | chromosome | KDE11344.1 |
| ***prmC*** | PrmC | Methane monooxygenase component C | 85% | 94% | chromosome | AII03498.1 | 87% | chromosome | KDE11343.1 |
| ***prmB*** | PrmB | Methane monooxygenase component A beta chain | 88% | 97% | chromosome | AII03497.1 | 89% | chromosome | KDE11342.1 |
| ***prmD*** | PrmD | Methane monooxygenase regulatory protein | 89% | 98% | chromosome | AII03496.1 | 89% | chromosome | KDE11341.1 |
