# Supplementary material for: Genome and Phenotype Microarray Analyses of Rhodococcus sp. BCP1 and Rhodococcus opacus R7: Genetic Determinants and Metabolic Abilities with Environmental Relevance
Source: PLoS One. 2015 Oct 1;10(10):e0139467. doi: 10.1371/journal.pone.0139467 (PMC4591350; doi:10.1371/journal.pone.0139467)
Supplement: S9 Table — (PDF) [file pone.0139467.s016.pdf]

				R. opacus R7			*Rhodococcus* sp. BCP1		
**Gene**	**Homologous protein**	**Function**	**R7 vs BCP1 (aa identity)**	**R7 vs RHA1 (aa identity)**	**Position in genome**	**Accession Number**	**BCP1 vs RHA1 (aa identity)**	**Position in genome**	**Accession Number**
akbA1a	**AkbA1a**	Ethylbenzene dioxygenase large subunit	36%	92%	pPDG5	AII11493.1	36%	pBMC2	KDE09919.1
akbA2a	**AkbA2a**	Ethylbenzene dioxygenase small subunit	43%	84%	pPDG5	AII11492.1	36%	pBMC2	KDE09920.1
akbA3	**AkbA3**	Ethylbenzene dioxygenase ferredoxin	/	69%	pPDG5	CP008952.1	/	/	/
akbA4	**AkbA4**	Ferredoxin reductase	39%	81%	pPDG5	AII11490.1	40%	chromosome	KDE12339.1
akbB	**AkbB**	Dihydrodiol dehydrogenase	47%	85%	pPDG5	AII11489.1	47%	pBMC2	KDE09922.1
akbC	**AkbC**	2,3-Dihydroxybiphenyl 1,2-dioxygenase	36%	87%	pPDG2	AII11058.1	35%	chromosome	KDE14642.1
akbD	**AkbD**	2-Hydroxy-6-oxo-6-phenylhexa-2,4-dienoate hydrolase	35%	67%	pPDG2	AII11051.1	33%	chromosome	KDE14641.1
akbE	**AkbE**	2-Hydroxypenta-2,4-dienoate hydratase	56%	60%	chromosome	AII11050.1	55%	chromosome	KDE14625.1
akbF	**AkbF**	4-Hydroxy-2-oxovalerate aldolase	/	63%	pPDG2	AII11049.1	/	/	/
