# Supplementary material for: Genome and Phenotype Microarray Analyses of Rhodococcus sp. BCP1 and Rhodococcus opacus R7: Genetic Determinants and Metabolic Abilities with Environmental Relevance
Source: PLoS One. 2015 Oct 1;10(10):e0139467. doi: 10.1371/journal.pone.0139467 (PMC4591350; doi:10.1371/journal.pone.0139467)
Supplement: S10 Table — (PDF) [file pone.0139467.s017.pdf]

				R. opacus R7			*Rhodococcus* sp. BCP1		
**Gene**	**Homologous protein**	**Function**	**R7 vs BCP1 (aa identity)**	**R7 vs RHA1 (aa identity)**	**Position in genome**	**Accession Number**	**BCP1 vs RHA1 (aa identity)**	**Position in genome**	**Accession Number**
dszA1	**DszA1**	Dibenzothiophene desulfurization enzyme	44%	98%	chromosome	AII08556.1	44%	chromosome	KDE15059.1
dszA2	**DszA2**	Dibenzothiophene desulfurization enzyme	44%	97%	chromosome	AII03608.1	44%	chromosome	KDE15059.1
dszB	**DszB**	Possible ABC sulfonate transporter	76%	98%	chromosome	AII06125.1	76%	chromosome	KDE15056.1
dszC1	**DszC1**	Probable dibenzothiophene desulfurization enzyme	67%	97%	chromosome	AII08748.1	66%	chromosome	KDE11236.1
dszC2	**DszC2**	Probable dibenzothiophene desulfurization enzyme	84%	96%	chromosome	AII08273.1	83%	chromosome	KDE11237.1
