# Supplementary material for: Genome and Phenotype Microarray Analyses of Rhodococcus sp. BCP1 and Rhodococcus opacus R7: Genetic Determinants and Metabolic Abilities with Environmental Relevance
Source: PLoS One. 2015 Oct 1;10(10):e0139467. doi: 10.1371/journal.pone.0139467 (PMC4591350; doi:10.1371/journal.pone.0139467)
Supplement: S11 Table — (PDF) [file pone.0139467.s018.pdf]

				R. opacus R7		*Rhodococcus* sp. BCP1	
**Gene**	**Homologous protein**	**Function**	**R7 vs BCP1 (aa identity)**	**Position in genome**	**Accession Number**	**Position in genome**	**Accession Number**
rub1bis	**Rub1bis**	Rubredoxin	/	pPDG4	DQ846881	/	/
narR1	**NarR1**	Regulator of GntR family	87%	pPDG4	ABH01023.1	pBMC2	KDE09916.1
narR2	**NarR2**	XylR-like regulator protein	94%	pPDG4	ABH01024.1	pBMC2	KDE09917.1
rub1/rub	**Rub1/Rub**	Rubredoxin	88%	pPDG4	ABH01026.1	pBMC2	KDE09915.1
rub2	**Rub2**	Rubredoxin	/	pPDG4	ABH01027.1	/	/
orf7	**Orf7**	Sterol-binding domain protein/ unknown	93%	pPDG4	ABH01028.1	pBMC2	KDE09918.1
narAa	**NarAa**	Naphthalene dioxygenase large subunit	90%	pPDG4	ABH01029.1	pBMC2	KDE09919.1
narAb	**NarAb**	Naphthalene dioxygenase small subunit	88%	pPDG4	ABH01030.1	pBMC2	KDE09920.1
narB	**NarB**	Cis-naphthalene dihydrodiol dehydrogenase	96%	pPDG4	ABH01031.1	pBMC2	KDE09922.1
