# Supplementary material for: Genome and Phenotype Microarray Analyses of Rhodococcus sp. BCP1 and Rhodococcus opacus R7: Genetic Determinants and Metabolic Abilities with Environmental Relevance
Source: PLoS One. 2015 Oct 1;10(10):e0139467. doi: 10.1371/journal.pone.0139467 (PMC4591350; doi:10.1371/journal.pone.0139467)
Supplement: S12 Table — (PDF) [file pone.0139467.s019.pdf]

				R. opacus R7		*Rhodococcus* sp. BCP1	
**Gene**	**Homologous protein**	**Function**	**R7 vs BCP1 (aa identity)**	**Position in genome**	**Accession number**	**Position in genome**	**Accession number**
genC	GenC	Salicylate hydroxylase	/	pPDG1<br>pPDG4	AII11448.1<br>AII10777.1	/	/
genB	GenB	Salicylate CoA syntethase	/	pPDG1<br>pPDG4	AII11449.1<br>AII10778.1	/	/
genA	GenA	Salicylate CoA ligase	/	pPDG1<br>pPDG4	AII11450.1<br>AII10779.1	/	/
genH	GenH	Gentisate dioxygenase	96%	pPDG1<br>pPDG4	AII11451.1<br>AII10780.1	chromosome	KDE14391.1
genI	GenI	3-Maleylpyruvate Isomerase	92%	pPDG1<br>pPDG4	AII11452.1<br>AII10781.1	chromosome	KDE14392.1
genL	GenL	Unknown function	89%	pPDG4	AII11453.1	chromosome	KDE14393.1
