# Supplementary material for: Genome and Phenotype Microarray Analyses of Rhodococcus sp. BCP1 and Rhodococcus opacus R7: Genetic Determinants and Metabolic Abilities with Environmental Relevance
Source: PLoS One. 2015 Oct 1;10(10):e0139467. doi: 10.1371/journal.pone.0139467 (PMC4591350; doi:10.1371/journal.pone.0139467)
Supplement: S13 Table — (PDF) [file pone.0139467.s020.pdf]

				***R. opacus* R7**			***Rhodococcus* sp. BCP1**		
**Gene**	**Homologous protein**	**Function**	**R7 vs BCP1 (aa identity)**	**R7 vs RHA1 (aa identity)**	**Position in genome**	**Accession Number**	**BCP1vs RHA1 (aa identity)**	**Position in genome**	**Accession Number**
bphAa	**BphAa**	Biphenyl-2,3-dioxygenase α subunit	36%	37%	pPDG5	AII11493.1	37%	pBMC2	KDE09919.1
bphAb	**BphAb**	Biphenyl-2,3-dioxygenase β subunit	36%	35%	pPDG5	AII11492.1	39%	pBMC2	KDE09920.1
bphAc	**BphAc**	Biphenyl-2,3-dioxygenase, ferredoxin component	33%	31%	chromosome	AII08472.1	31%	chromosome	KDE10172.1
bphAd	**BphAd**	Biphenyl-2,3-dioxygenase, reductase	33%	33%	pPDG5	AII11490.1	37%	chromosome	KDE10578.1
bphB	**BphB**	Cis-2,3-dihydrobiphenyl-2,3-diol dehydrogenase	47%	78%	pPDG5	AII11489.1	45%	pBMC2	KDE09922.1
bphC	**BphC**	2,3-Dihydroxybiphenyl-1,2-dioxygenase	36%	35%	pPDG2	AII11058.1	35%	chromosome	KDE14642.1
akbD	**AkbD**	2-Hydroxy-6-oxohepta-2,4-dienoate hydrolase	35%	38%	pPDG2	AII11051.1	53%	chromosome	KDE11753.1
bphE	**BphE**	2-Oxopent-4-enoate hydratase	59%	66%	chromosome	AII03622.1	74%	chromosome	KDE14453.1
bphF	**BphF**	4-Hydroxy-2-oxovalerate aldolase	87%	89%	chromosome	AII03620.1	91%	chromosome	KDE14451.1
bphG	**BphG**	Acetaldehyde dehydrogenase	76%	74%	chromosome	AII03621.1	83%	chromosome	KDE14452.1
