# Supplementary material for: Genome and Phenotype Microarray Analyses of Rhodococcus sp. BCP1 and Rhodococcus opacus R7: Genetic Determinants and Metabolic Abilities with Environmental Relevance
Source: PLoS One. 2015 Oct 1;10(10):e0139467. doi: 10.1371/journal.pone.0139467 (PMC4591350; doi:10.1371/journal.pone.0139467)
Supplement: S14 Table — (PDF) [file pone.0139467.s021.pdf]

				***R. opacus* R7**			***Rhodococcus* sp. BCP1**		
**Gene**	**Homologous protein**	**Function**	**R7 vs BCP1 (aa identity)**	**R7 vs RHA1 (aa identity)**	**Position in genome**	**Accession Number**	**BCP1 vs RHA (aa identity)**	**Position in genome**	**Accession Number**
badI	**BadI**	Naphthoate synthase	89%	99%	chromosome	AII08541.1	87%	chromosome	KDE15145.1
badH1	**BadH1**	2-Hydroxycyclohexanecarboxyl-CoA dehydrogenase	83%	99%	chromosome	AII08542.1	83%	chromosome	KDE15144.1
badH2	**BadH2**	2-Hydroxycyclohexanecarboxyl-CoA dehydrogenase	/	/	/	/	/	chromosome	KDE12227.1
aliA	**AliA**	Long-chain-fatty-acid-CoA ligase	71%	95%	chromosome	AII08543.1	71%	chromosome	KDE15143.1
badJ	**BadJ**	Acyl-CoA dehydrogenase	87%	99%	chromosome	AII08544.1	87%	chromosome	KDE15141.1
pobA	**PobA**	*p*-Hydroxybezoate hydroxylase	77%	98%	chromosome	AII08627.1	78%	chromosome	KDE11135.1
