# Supplementary material for: Genome and Phenotype Microarray Analyses of Rhodococcus sp. BCP1 and Rhodococcus opacus R7: Genetic Determinants and Metabolic Abilities with Environmental Relevance
Source: PLoS One. 2015 Oct 1;10(10):e0139467. doi: 10.1371/journal.pone.0139467 (PMC4591350; doi:10.1371/journal.pone.0139467)
Supplement: S15 Table — (PDF) [file pone.0139467.s022.pdf]

				R. opacus **R7**			***Rhodococcus*** sp. **BCP1**		
**Gene**	**Homologous protein**	**Function**	**R7 vs BCP1 (aa identity)**	**R7 vs RHA1 (aa identity)**	**Position in genome**	**Accession Number**	**BCP1 vs RHA1 (aa identity)**	**Position in genome**	**Accession Number**
catA1	**CatA1**	Catechol 1,2 dioxygenase	68%	99%	chromosome	AII08813.1	68%	chromosome	KDE10959.1
catB1	**CatB1**	Muconate cycloisomerase	78%	98%	chromosome	AII08814.1	78%	chromosome	KDE10958.1
catC	**CatC**	Muconolactone isomerase	89%	96%	chromosome	AII08815.1	87%	chromosome	KDE10957.1
catA2	**CatA2**	Catechol 1,2 dioxygenase	/	/	chromosome	CP008947.1	/	/	/
catB2	**CatB2**	Muconate cycloisomerase	/	/	chromosome	AII05696.1	/	/	/
