# Supplementary material for: Genome and Phenotype Microarray Analyses of Rhodococcus sp. BCP1 and Rhodococcus opacus R7: Genetic Determinants and Metabolic Abilities with Environmental Relevance
Source: PLoS One. 2015 Oct 1;10(10):e0139467. doi: 10.1371/journal.pone.0139467 (PMC4591350; doi:10.1371/journal.pone.0139467)
Supplement: S16 Table — (PDF) [file pone.0139467.s023.pdf]

				R. opacus R7			*Rhodococcus* sp. BCP1		
**Gene**	**Homologous Protein**	**Function**	**R7 vs BCP1 (aa identity)**	**R7 vs RHA1 (aa identity)**	**Position in genome**	**Accession Number**	**BCP1 vs RHA1 (aa identity)**	**Position in genome**	**Accession Number**
pcaI	**PcaI**	Succinyl-CoA 3-ketoacid-coenzyme A transferase subunit B	77%	99%	chromosome	AII09804.1	76%	chromosome	KDE10925.1
pcaJ	**PcaJ**	Succinyl-CoA 3-ketoacid-coenzyme A transferase subunit A	80%	100%	chromosome	AII09803.1	80%	chromosome	KDE10926.1
pcaH	**PcaH**	Protocatechuate 3,4-dioxygenase beta chain	71%	97%	chromosome	AII09802.1	71%	chromosome	KDE10927.1
pcaG	**PcaG**	Protocatechuate 3,4-dioxygenase alpha chain	57%	99%	chromosome	AII09801.1	57%	chromosome	KDE10928.1
pcaB	**PcaB**	3-Carboxy-cis,cis-muconate cycloisomerase	61%	98%	chromosome	AII09800.1	61%	chromosome	KDE10929.1
pcaL	**PcaL**	3-Oxoadipate enol-lactone hydrolase	63%	97%	chromosome	AII09799.1	63%	chromosome	KDE10930.1
pcaF	**PcaF**	Acetyl-CoA acetyltransferase	82%	98%	chromosome	AII09797.1	82%	chromosome	KDE10932.1
