# Supplementary material for: Genome and Phenotype Microarray Analyses of Rhodococcus sp. BCP1 and Rhodococcus opacus R7: Genetic Determinants and Metabolic Abilities with Environmental Relevance
Source: PLoS One. 2015 Oct 1;10(10):e0139467. doi: 10.1371/journal.pone.0139467 (PMC4591350; doi:10.1371/journal.pone.0139467)
Supplement: S17 Table — (PDF) [file pone.0139467.s024.pdf]

				R. opacus R7			*Rhodococcus* sp. BCP1		
**Gene**	**Homologous protein**	**Function**	**R7 vs BCP1 (aa identity)**	**R7 vs RHA1 (aa identity)**	**Position in genome**	**Accession Number**	**Position in genome**	**BCP1 vs RHA1 (aa identity)**	**Accession Number**
paaG	**PaaG**	Phenylacetate-CoA oxygenase, PaaG subunit	/	99%	chromosome	AII08339.1	/	/	/
paaH	**PaaH**	Phenylacetate-CoA oxygenase, PaaH subunit	/	100%	chromosome	AII08340.1	/	/	/
paaI	**PaaI**	Phenylacetate-CoA oxygenase, PaaI subunit	/	96%	chromosome	AII08341.1	/	/	/
paaJ	**PaaJ**	Phenylacetate-CoA oxygenase, PaaJ subunit	/	98%	chromosome	AII08342.1	/	/	/
paaK	**PaaK**	Phenylacetate-CoA oxygenase/reductase, PaaK subunit	35%	96%	chromosome	AII08343.1	chromosome	34%	KDE12334.1
paaF	**PaaF**	Phenylacetate-coenzyme A ligase PaaF	/	98%	chromosome	AII08344.1	/	/	/
paaE	**PaaE**	Acetyl-CoA acetyltransferase	74%	98%	chromosome	AII08335.1	chromosome	74%	KDE15146.1
paaA	**PaaA**	Enoyl-CoA hydratase	32%	97%	chromosome	AII08336.1	chromosome	< 30%	/
paaC	**PaaC**	3-Hydroxyacyl-CoA dehydrogenase	66%	95%	chromosome	AII08337.1	chromosome	66%	KDE15147.1
paaB	**PaaB**	Enoyl-CoA hydratase	35%	97%	chromosome	AII08338.1	chromosome	< 30%	/
paaZ	**PaaZ**	Aldehyde dehydrogenase	31%	97%	chromosome	AII08334.1	chromosome	< 30%	/
paaD	**PaaD**	Phenylacetic acid degradation protein, thioesterase	/	97%	chromosome	AII08332.1	/	/	/
paaL	**PaaL**	Acetate permease ActP (cation/acetate symporter)	42%	99%	chromosome	AII08348.1	chromosome	43%	
