# Supplementary material for: Genome and Phenotype Microarray Analyses of Rhodococcus sp. BCP1 and Rhodococcus opacus R7: Genetic Determinants and Metabolic Abilities with Environmental Relevance
Source: PLoS One. 2015 Oct 1;10(10):e0139467. doi: 10.1371/journal.pone.0139467 (PMC4591350; doi:10.1371/journal.pone.0139467)
Supplement: S18 Table — (PDF) [file pone.0139467.s025.pdf]

				***R. opacus* R7**			***Rhodococcus* sp. BCP1**		
**Gene**	**Homologous protein**	**Function**	**R7 vs BCP1 (aa identity)**	**R7 vs RHA1 (aa identity)**	**Position in genome**	**Accession Number**	**BCP1vs RHA1 (aa identity)**	**Position in genome**	**Accession Number**
hmgA	**HmgA**	Homogentisate 1,2-dioxygenase	85%	97%	chromosome	AII08874.1	85%	chromosome	KDE10817.1
fahA	**FahA**	Fumarylacetoacetase	79%	97%	chromosome	AII08876.1	80%	chromosome	KDE10815.1
mai	**Mai**	Enoyl-CoA hydratase/isomerase	79%	97%	chromosome	AII08877.1	80%	chromosome	KDE11332.1
orf1	**Orf1**	Long-chain-fatty-acid-CoA ligase	78%	97%	chromosome	AII08879.1	78%	chromosome	KDE10810.1
